# Pyruvate Kinase M2 Activates mTORC1 by Phosphorylating AKT1S1

**DOI:** 10.1038/srep21524

**Published:** 2016-02-15

**Authors:** Chang-Liang He, Yang-Yang Bian, Yu Xue, Ze-Xian Liu, Kai-Qiang Zhou, Cui-Fang Yao, Yan Lin, Han-Fa Zou, Fang-Xiu Luo, Yuan-Yuan Qu, Jian-Yuan Zhao, Ming-Liang Ye, Shi-Min Zhao, Wei Xu

**Affiliations:** 1 State Key Lab of Genetic Engineering, Obstetrics & Gynecology Hospital of Fudan University and School of Life Sciences, Shanghai 200090, P.R. China; 2 Institutes of Biomedical Sciences and Collaborative Innovation Center for Genetics and Development Biology, Fudan University, Shanghai 200032, P.R. China; 3 Chinese Academy of Sciences, Dalian Institute Chemical Physics, National Chromatography R&A Center, Key Lab Separation Science Analytic Chemistry, Dalian 116023, P.R. China; 4 Department of Medical Engineering, College of Life Sciences and Technology, Huazhong University of Science and Technology, Wuhan 430074, P.R. China; 5 Collaborative Innovation Center for Biotherapy, West China Hospital, Sichuan University, Chengdu, 610041, P.R. China; 6 Department of Pathology, Affiliated Ruijin Hospital of Shanghai Jiaotong University, Shanghai, 201821 P.R. China; 7 Department of Urology, Fudan University Shanghai Cancer Center, Shanghai 200032, P.R. China; 8 Department of Oncology, Shanghai Medical College, Fudan University, Shanghai 200032, P.R. China

## Abstract

In cancer cells, the mammalian target of rapamycin complex 1 (mTORC1) that requires hormonal and nutrient signals for its activation, is constitutively activated. We found that overexpression of pyruvate kinase M2 (PKM2) activates mTORC1 signaling through phosphorylating mTORC1 inhibitor AKT1 substrate 1 (AKT1S1). An unbiased quantitative phosphoproteomic survey identified 974 PKM2 substrates, including serine202 and serine203 (S202/203) of AKT1S1, in the proteome of renal cell carcinoma (RCC). Phosphorylation of S202/203 of AKT1S1 by PKM2 released AKT1S1 from raptor and facilitated its binding to 14-3-3, resulted in hormonal- and nutrient-signals independent activation of mTORC1 signaling and led accelerated oncogenic growth and autophagy inhibition in cancer cells. Decreasing S202/203 phosphorylation by TEPP-46 treatment reversed these effects. In RCCs and breast cancers, PKM2 overexpression was correlated with elevated S202/203 phosphorylation, activated mTORC1 and inhibited autophagy. Our results provided the first phosphorylome of PKM2 and revealed a constitutive mTORC1 activating mechanism in cancer cells.

The mTORC1 complex integrates growth factor signals with the nutrients signals to control cell growth and proliferation^1^. The availability of growth factors, free essential amino acids and glucose determines cell growth and proliferation. In cancer cells, mTORC1 is constitutively activated regardless the fluctuation of growth factors and nutrients^2^,^3^. This suggests that cancer cells may employ unique mechanism to activate mTORC1 and provide survival, and growth and proliferation advantages over normal cells.

The mTORC1 is a major anabolic regulator that controls an array of macromolecule biosynthetic processes, such as protein translation, mRNA transcription, ribosome biogenesis, lipid biogenesis, autophagy, mitochondrial function and the immune response^4^. The activity of mTORC1 is sensitive to rapamycin, insulin, insulin like growth factor 1 (IGF1), oxygen and amino acids signals and is suppressed by AKT1 substrate 1 (AKT1S1) through binding to regulatory-associated protein of mTOR (raptor), a component of mTORC1^5^,^6^. Constitutively activation of mTORC1 in cancer cells not only ensure their switches from catabolic metabolism to anabolic metabolism that is required to sustain their unconstrained growth^7^, but also acquire other cancer-promoting consequences such as autophagy inhibition^8^. Oncogene mutations, such as in PI3K, Ras, Raf, growth factor receptor kinases and autocrine growth factors^9^,^10^,^11^, or inactivation of tumor suppressors such as PTEN, AMPK, TSC2, LKB1, NF1^12^,^13^ are all found to be able to activate mTORC1. However, unique common mTORC1 activating mechanisms may exist since these mutations may not always exist in one type of cancers.

Pyruvate kinase (E.C. 2.7.1.40) is a rate-limiting glycolysis enzyme that catalyzes the transfer of a phosphate group from phosphoenolpyruvate (PEP) to ADP, resulting in the formation of pyruvate and ATP^14^. Among the four pyruvate kinase isoforms expressed in mammals is the M1 isoform (PKM1), which is expressed in most adult tissues; the L and R isoforms, which are specifically expressed in liver and red blood cells^15^,^16^, respectively; and the M2 isoform (PKM2), which is expressed during embryonic development and in most adult cells, except in adult muscle, brain and liver cells^17^. The amino acid sequence of PKM2 is identical to PKM1, except for a 23 amino acid stretch (a.a. 378–434) at its C-terminus. The c-Myc-heterogeneous nuclear ribonucleoprotein-dependent alternative splicing of exon 9 and exon 10 of the transcript of the PKM gene result in PKM1 and PKM2, respectively^18^. Exon 9-containing PKM1 exists as a glycolytically active stable tetramer, and exon 10-containing PKM2 exists in a dynamic equilibrium between a glycolytically inactive dimer and a glycolytically active tetramer.

Proposed underlying tumorigenic mechanisms of PKM2 include facilitating anabolic metabolism by diverting glycolytic intermediary metabolites to anabolic pathways^17^. The introduction of PKM2, but not glycolytic active PKM1, into PKM2 knockdown cancer cells restored their ability to form tumor xenografts^17^, showing that the non-glycolytic functions of PKM2 are needed to sustain cancer growth. Moreover, switch PKM2 from dimer to tetramer by small molecule TEPP-46 inhibited oncogenic growth of xenograft tumors^19^, highlighting tumorigenic importance of dimeric PKM2. Upon stimulation by epidermal growth factor (EGF), interleukin-3 or apoptotic signals, dimeric PKM2 translocate into the nucleus and display various functions^20^,^21^. For example, nuclear PKM2 associates with chromatin^20^,^22^, binds to the C-terminus of Oct-4 and enhances Oct-4-mediated transcription^23^, binds to HIF1 to recruit the p300 transcriptional co-activator to enhance the hypoxic transcriptional response^24^ and contributes to the transactivation of cyclin D and c-Myc^25^,^26^. Remarkably, the kinase activity of PKM2 is required for its nucleic actions, implying that PKM2 is either a protein kinase or that metabolites associated with PKM2 are required for these functions.

Dimeric PKM2 was initially found to phosphorylate histone H3 at T11 upon EGF receptor activation^27^, exhibit cysteine-dependent histone H1 phosphorylation activity^28^ and activate the transcription of MEK5 by phosphorylating stat3 at Y705^29^. Later, a protein array assay showed that SAICAR-bound PKM2 phosphorylates an array of substrates and, in particular, activates Erk1/2 signaling to induce cell proliferation^30^. These findings suggested that PKM2 is a multi-specific protein kinase that regulates a number of substrates. Notably, the protein kinase activity of PKM2 was also challenged by some experiments^31^ while results from yeast protein equivalent to PKM2 reassure protein kinase of PKM2^32^. A quantitative phosphoproteomic approach employs a fixed proteome as the substrates^33^,^34^ made cell-wide screening of PKM2 substrates possible and may provide answers for whether PKM2 has protein kinase activity.

## Results

### PKM2 Substrates Survey by Quantitative Phosphoproteomic Approach

**Figure 1:**
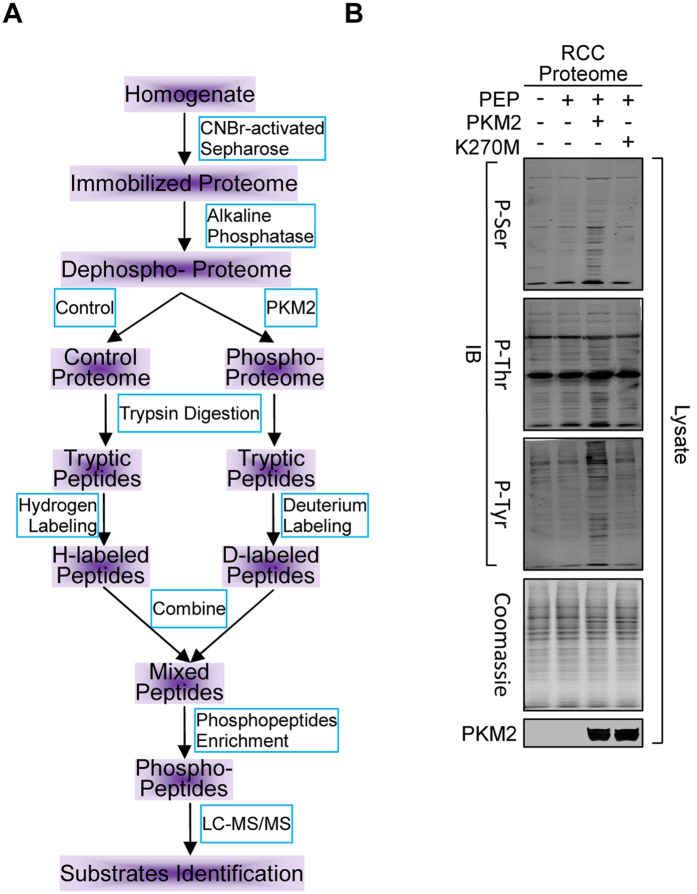
Quantitative Phosphoproteomic Approach to Survey PKM2 Substrates. (**A**) Schematic diagram of the PKM2 substrate survey strategy. Proteins in RCC lysate were treated as indicated in the blue brackets, the resulted peptides were labeled, mixed and enriched for phosphopeptides, followed by LC-MS/MS analysis to identify PKM2 substrates. (**B**) Proteins in a lysate of RCC were de-phosphorylated with alkaline phosphatase and precipitated with acetone. The redissolved proteins were treated with recombinant PKM2 or PKM2^K270M^ (K270M), employing PEP as the phosphor-donor. The levels of P-Ser, P-Thr and P-Tyr in proteins were determined after treatments.

To identify substrates of PKM2, we adopted a quantitative phosphoproteomic approach^34^,^35^. Briefly, proteome of RCC were cross-linked onto Sephoraose-4B resin to prevent endogenous kinases from accessing to their substrates. Alkaline phosphatase was used to remove existing phosphorylation in the proteome. Following removal of alkaline phosphatase by washing, the phosphorylation free proteome was re-phosphorylated by PKM2 with PEP as the phospho-donor. PKM2-untreated and -treated tryptic peptides library were each labeled with hydrogen and deuterium formaldehyde and sodium cyanoborohydride (NaBH_3_CN), respectively. Phospho-peptides were enriched by Ti4^+^ -IMAC microspheres and the relative abundance between PKM2-untreated and -treated phosphor-peptides were compared by liquid chromatography followed by tandem mass spectrometry (LC-MS/MS) (Fig. 1A).

Consistent with that wild-type PKM2, not its inactive PKM2^K270M^ mutant^36^ increased the levels of the phosphoserine (P-Ser), phosphothreonine (P-Thr) and phosphotyrosine (P-Tyr) of RCC proteome in a PEP dependent manner (Fig. 1B), a total of 974 residues in 405 proteins, including 13 of the PKM2 substrates previously identified by the protein array assay^30^, were identified as potential PKM2 substrates (Table S1). A twofold increase in the phosphorylation levels of treated over untreated peptides was employed as cutoff for high-confidence (>99%) *in vitro* substrates identification, as this ratio was well beyond the range of inherent variability.

### Bioinformatic Characterization of PKM2 Substrates

**Figure 2:**
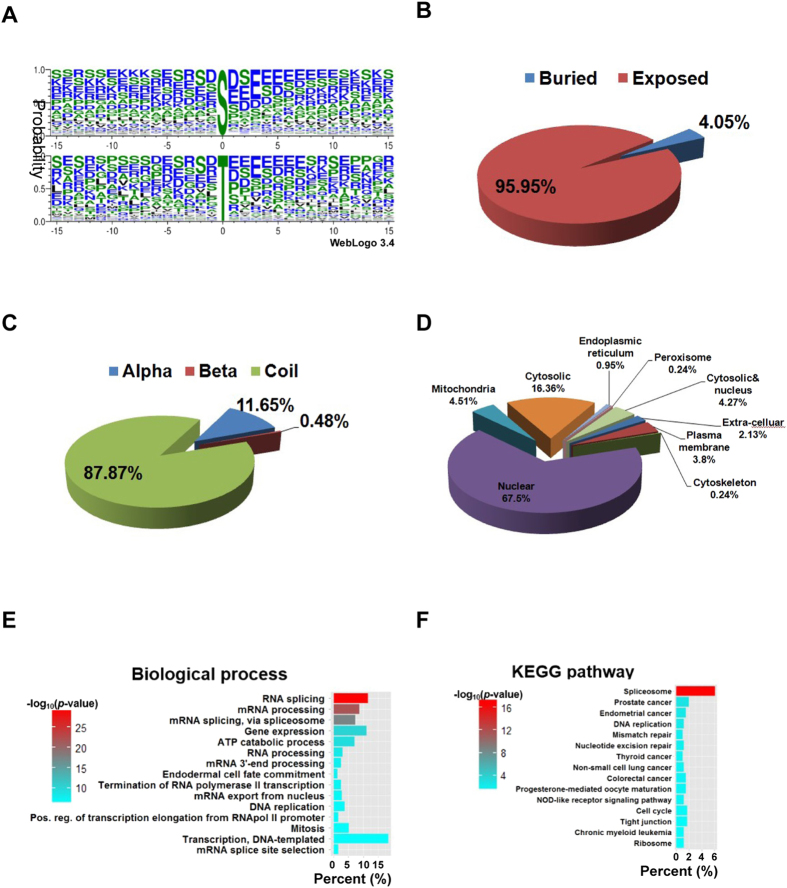
Bioinformatics analysis of the substrates of PKM2. (**A**) The PKM2 phosphorylated serine (upper) and threonine (lower) sites identified were aligned and their spanning amino acid residues were visualized with WebLogo. (**B–D**) The solvent accessibility (**B**), secondary structures distributions (**C**) and sub-cellular localizations (**D**) of PKM2 substrates were predicted with NetSurfP 1.1, ESpritz and WoLF PSORT, respectively. (**E,F**) The enriched biological processes (**E**) and the enriched KEGG pathways (**F**) of identified substrates are demonstrated with heatmaps.

Among the positively identified substrates of PKM2, 876 are serines, 81 are threonines and 17 are tyrosines, confirmed that PKM2 is a multi-specific protein kinase. WebLogo analysis^37^ showed that PKM2 prefers to phosphorylate serine or threonine residues spanned by acidic amino acids (Fig. 2A). Motif-x analysis^38^ revealed no strong consensus sequences in the substrates of PKM2 (Figure S1A). Moreover, as estimated by NetSurfP 1.1^39^, 95.95% of the phosphorylation sites of PKM2 are solvent exposed (Fig. 2B), and the vast majority (96.36%) of the resides phosphorylated by PKM2 are predicted^40^ to be within the disordered regions of proteins (Figure S1B). These results were consistent with the fact that PKM2 predominantly phosphorylates sites in coils (87.87%) rather than in more ordered regions, such as α-helices (11.65%) and β-sheets (0.48%)(Fig. 2C).

Cellular compartment analysis by WoLF PSORT^41^ revealed that 67.5% of PKM2 substrates are nuclear proteins (Fig. 2D), consistent with the fact that dimeric PKM2 translocates into the nucleus and phosphorylates proteins in there^27^,^28^,^29^. This result also suggested that PKM2 regulates the functions of large number of nuclear proteins, albeit PKM2 also exerts its regulatory function in multiple compartments, as substrates of PKM2 were found in the cytosol (16.36%), cytosol and nucleus (4.27%), mitochondria (4.51%), plasma membrane (3.80%), endoplasmic reticulum (0.95%) and peroxisome (0.24%) (Fig. 2D).

Gene ontology annotations^42^ revealed that the substrates of PKM2 are involved in diverse biological functions, ranging from RNA processing to mitosis (Fig. 2E). The mechanisms of actions of PKM2 substrates include protein binding, DNA binding, RNA binding, helicases, histone demethylases and transcription factors (Figure S1C). Remarkably, KEGG GO enrichment analysis revealed that PKM2 substrates are enriched in prostate, endometrial thyroid, colorectal, chronic myeloid leukemia and non-small cell lung cancer pathways, as well as in cancer associated pathways, such as DNA repair and cell cycle pathways (Fig. 2F), in line with the facts that PKM2 is preferentially expressed in most cancers. Collectively, these results suggest that PKM2 phosphorylates an array of substrates and regulates a plethora of cancer associated cellular functions.

Tyrosine substrates of PKM2 were not subject to bioinformatics analysis because the number of identifications was insufficient for statistical analysis, due to that insufficient extraction of membrane fraction, where most phospho-tyrosine proteins are located. In line with this expectation, we did see increase of phosphor-tyrosine in the membrane fraction by PKM2 overexpression (Figure S1D).

### PKM2 Phosphorylates S202 and S203 of AKT1S1

**Figure 3:**
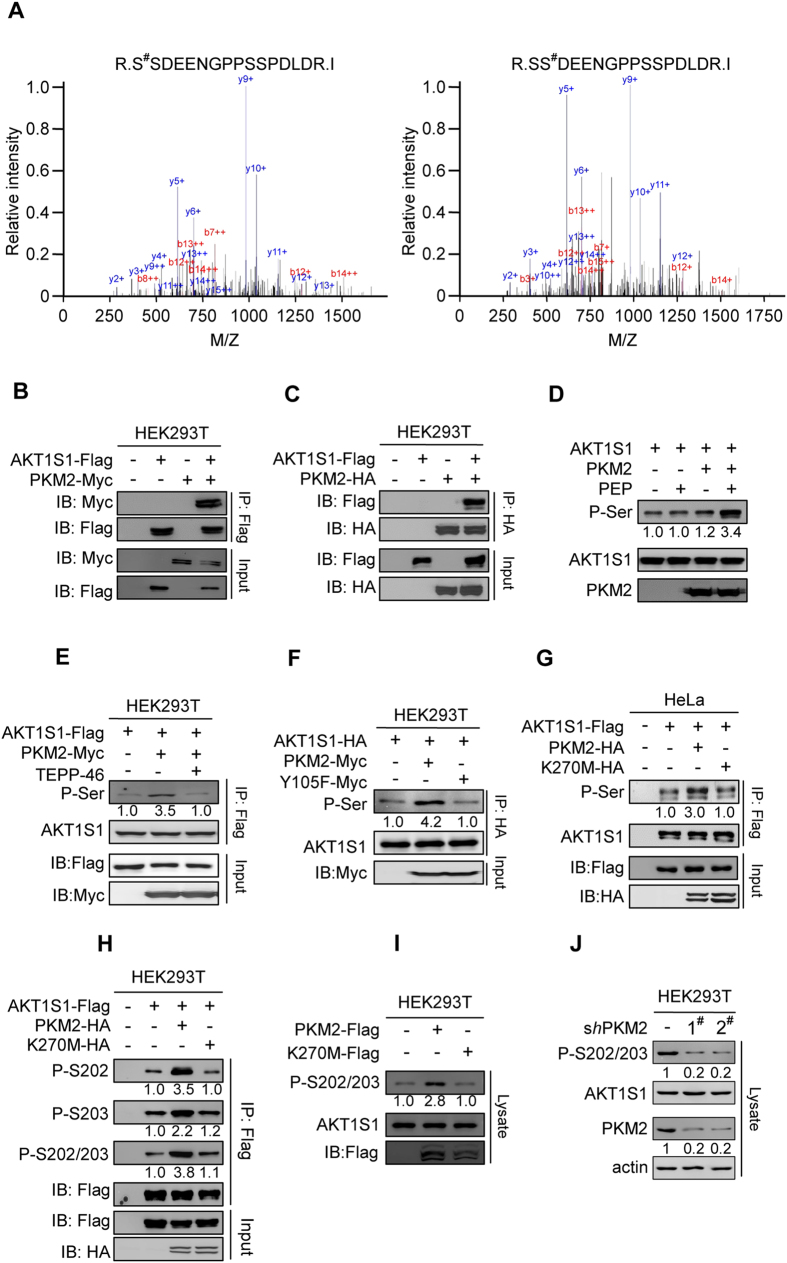
Serine202 and serine203 are phosphorylated by PKM2. (**A**) MS/MS spectra of tryptic peptides from the PKM2-treated HEK293T proteome that led to the identification of phosphorylation of serine202 (left) or serine 203 (right) of AKT1S1. (**B**) Flag-tagged AKT1S1 and Myc-tagged PKM2 were co-expressed in HEK293T cells. The PKM2 co-purified with the AKT1S1 was detected by Myc antibody. (**C**) Flag-tagged AKT1S1 and HA-tagged PKM2 were co-expressed in HEK293T cells. The AKT1S1 co-purified with the PKM2 was detected by Flag antibody. (**D**)Purified AKT1S1 was treated with recombinant PKM2 in the presence and absence of PEP, and the levels of P-Ser of AKT1S1 in the reaction mixture after treatment were determined. Numerical values below the gels indicate quantification of the bands relative to untreated AKT1S1 (hereinafter). (**E**) Flag-tagged AKT1S1 was co-expressed with PKM2. P-ser levels of AKT1S1 from cells cultured with and without TEPP-46 (100 nM) supplementation were determined. (**F**) Flag-tagged AKT1S1 was co-expressed with PKM2 or PKM2^Y105F^ (Y105F), P-ser levels of AKT1S1 purified from different cells were determined. (**G**) Flag-tagged AKT1S1 was co-expressed with either HA-tagged PKM2 or HA-tagged PKM2^K270^ mutant (K270M) in HeLa cells. The P-Ser levels of Flag bead-purified AKT1S1 from each culture were determined and quantified. (**I**) Purified AKT1S1 was treated with either purified PKM2 or purified K270M. The P-S202, P-S203 and P-S202/203 levels of each treated AKT1S1 were determined by site-specific antibodies and quantified. The relative intensities of phosphorylation signals were normalized to those of untreated AKT1S1. (**J**) Flag-tagged PKM2 or Flag-tagged K270M was overexpressed in HEK293T cells. The endogenous P-S202/203 levels of AKT1S1 of each culture were determined and the relative intensities of P-S202/203 signals were normalized to that of HEK293T cells. (**K**) The endogenous P-S202/203 levels of HEK293T cells before and after *PKM2* knockdown by independent *shRNAs* were compared. The PKM2 knockdown efficiency was confirmed by western blot.

The phosphorylation levels of serine 202 (S202) and serine 203 (S203) of mTORC1 inhibitor AKT1S1 were increased by 177.0 fold and 137.7 fold, respectively, by PKM2 treatment (Fig. 3A, Table S1), suggested that functions of AKT1S1 and activity of mTORC1 may be regulated by PKM2. This hypothesis was further confirmed. When they are co-expressed in HEK293T cells, PKM2 was co-purified with AKT1S1 (Fig. 3B) and AKT1S1 was also co-purified with PKM2 (Fig. 3C), showed that PKM2 and AKT1S1 interact with each other. Moreover, incubation of purified AKT1S1 with recombinant PKM2 resulted in an elevation of the P-Ser levels of AKT1S1 in a PEP dependent manner (Fig. 3D), confirmed that PKM2 phosphorylates AKT1S1 directly. However, supplementation TEPP-46 to the culture media to promote PKM2 tetramer formation in HEK293T cells^43^ diminished PKM2’s ability to phosphorylate AKT1S1 (Fig. 3E). This, together with that overexpressing constitutive glycolytic active PKM2^Y105F^ 44 mutant failed to phosphorylate AKT1S1 (Fig. 3F), and only co-expression of wild-type PKM2, but not an inactive PKM2^K270M^ mutant with AKT1S1 augmented the P-Ser level of AKT1S1 (Fig. 3G), confirmed that protein kinase active dimeric PKM2, but not tetrameric glycolytic active PKM2, phosphorylates AKT1S1.

To verify that S202 and S203 of AKT1S1 are substrates of PKM2, we generated site-specific antibodies against phospho-S202 (P-S202), phospho-S203 (P-S203) and phospho-S202/203 (P-S202/203) of AKT1S1 by employing synthetic phosphorylated AKT1S1 peptides as antigens (Figure S2). The levels of P-S202, P-S203 and P-S202/203 of affinity purified AKT1S1 were increased by recombinant PKM2, but not PKM2^K270M^, treatment (Fig. 3H), confirmed that both S202 and S203 of AKT1S1 are substrates of PKM2. Taking the advantage of that the P-S202/203 antibody was able to detect changes of endogenous AKT1S1 phosphorylation, we traced the P-S202/203 changes when PKM2 was over- or under-expressed in cells. Overexpression of PKM2, but not the PKM2^K270M^ mutant, in HEK293T cells increased the levels of endogenous P-S202/203 (Fig. 3I). Conversely, depletion of PKM2 with independent *shRNAs* decreased the levels of endogenous P-S202/S203 in HEK293T cells (Fig. 3J). These results collectively support that PKM2 phosphorylates S202/S203 of AKT1S1.

### Phosphorylation of S202/S203 of AKT1S1 Dissociates AKT1S1 from Raptor

**Figure 4:**
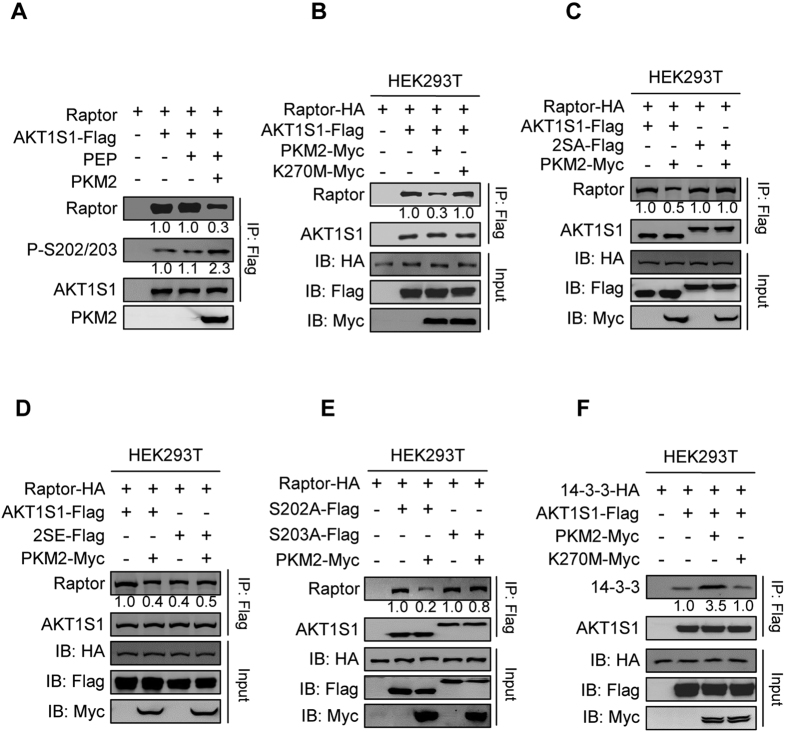
PKM2 releases AKT1S1 from raptor and promotes AKT1S1-14-3-3 interaction by phosphorylating S202/203 of AKT1S1. (**A**) Purified raptor was incubated with AKT1S1, AKT1S1 + PEP or AKT1S1 + PEP + PKM2. After incubation, AKT1S1 was purified by Flag beads. The amount of raptor co-purified with AKT1S1 and the PS202/203 level of AKT1S1 were determined and normalized to that of AKT1S1 incubated with raptor without other components. (**B**) Raptor and AKT1S1 were co-expressed with either PKM2 or K270M in HEK293T cells. The amount of raptor co-precipitated with AKT1S1 from different cells was determined. (**C**) Raptor was co-expressed with either AKT1S1 or 2SA in HEK293T cells. The amount of raptor co-immunoprecipitated with AKT1S1 or 2SA in the presence and absence of PKM2 was compared. (**D**) Raptor was co-expressed with either AKT1S1 or 2SE in HEK293T cells. The amount of raptor co-immunoprecipitated with AKT1S1 or 2SE in the presence and absence of PKM2 was determined and raptor signals were normalized to that co-expressed with AKT1S1 alone. (**E**) Raptor was co-expressed with either S202A or S203A in HEK293T cells. The amount of raptor co-immunoprecipitated with S202A or S203A in the presence and absence of PKM2 was determined and compared. (**F**) 14-3-3 and AKT1S1 were co-expressed with either PKM2 or K270M in HEK293T cells. The amount of 14-3-3 co-immunoprecipitated with AKT1S1 was compared under different co-expression conditions.

Phosphorylation on sites of AKT1S1, such as on threonine 246, releases AKT1S1 from raptor and activates mTORC1^45^. We thus tested whether the phosphorylation on S202/S203 of AKT1S1 by PKM2 also affects the AKT1S1-raptor interaction. Purified AKT1S1 treated by recombinant PKM2 and PEP had increased P-S202/203 levels but significantly lower ability to pull down raptor (Fig. 4A), imply that phosphorylation on S202/S203 of AKT1S1 decrease interaction between AKT1S1 and raptor. Supporting this notion, overexpression of PKM2, but not kinase inactive PKM2^K270M^, decreased the interaction between AKT1S1 and raptor in HEK293T cells (Fig. 4B). Moreover, PKM2 overexpression failed to alter the interactions between raptor and AKT1S1^S202A,S203A^ (2SA) or AKT1S1^S202E,S203E^ (2SE) (Fig. 4C,D). These results collectively suggested that PKM2 regulates AKT1S1-raptor interaction by phosphorylating S202/203 of AKT1S1. Furthermore, overexpression of PKM2 in HEK293T cells greatly decreased the interaction between the S202 to alanine AKT1S1 (S202A) mutant and raptor, but only slightly decreased the interaction between the S203 to alanine AKT1S1 (S203A) mutant and raptor (Fig. 4E), suggest that PKM2 mediates AKT1S1-raptor interaction mainly through phosphorylating S203 of AKT1S1.

### Phosphorylation of S202/S203 Promotes AKT1S1 Binding to 14–3–3

A partition of phosphorylated AKT1S1 to scaffold protein 14–3–3 to decrease free AKT1S1 pool also serves as a mechanism to activate mTORC1. We therefore further tested whether the phosphorylation of S202 and S203 drives AKT1S1 from raptor to 14–3–3. *In vitro* treatment of purified AKT1S1 by recombinant PKM2 increased its interaction with 14–3–3; however, the same treatment failed to increase the interaction between the 2SA mutant and 14–3–3 (Figure S3A). In HEK293T cells, overexpression of PKM2, but not PKM2^K270M^, caused a more than threefold increase in the AKT1S1–14–3–3 interaction (Fig. 4F), confirmed that kinase activity of PKM2 regulates the interaction between AKT1S1 and 14–3–3 in cells. Moreover, PKM2 overexpression in HEK293T cells failed to increase 2SE’s ability to bind to 14–3–3 (Figure S3B). These results confirmed that PKM2 regulates AKT1S1–14–3–3 interaction through phosphorylating S202/S203 of AKT1S1.

### Phosphorylation of S203/S202 of AKT1S1 by PKM2 Activates mTORC1 Independent of Growth Factors and Amino Acids Signals

**Figure 5:**
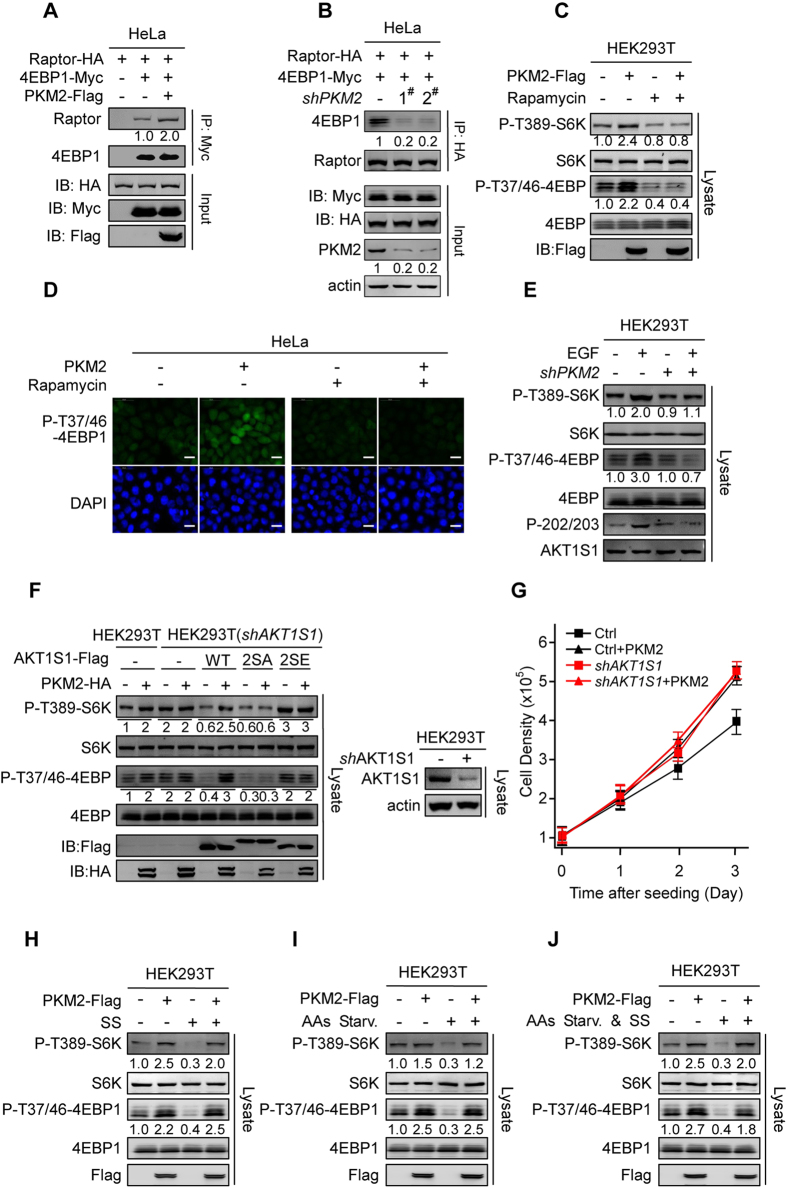
PKM2 activates mTORC1 signaling and promotes cell proliferation by phosphorylating S202/203 of AKT1S1. (**A**) Raptor was co-expressed with 4E-BP1 in HeLa cells and in PKM2 overexpressing HeLa cells. The amount of raptor co-immunoprecipitated with 4E-BP1 in different cells was determined by western blot and normalized to that of without PKM2 expression. (**B**) Raptor was co-expressed with 4E-BP1 in HeLa cells and in *PKM2* knockdown HeLa cells. The amount of 4E-BP1 co-immunoprecipitated with raptor in different cells and was compared. (**C**) The levels of P-T389-S6K and P-T37/46-4EBP in HEK293T cells and in PKM2 overexpressing HEK293T cells were detected and quantified under with or without rapamycin supplementation in the culture media, respectively. All intensities of P-T389-S6K and P-T37/46-4EBP signals were normalized to those of untreated cells. (**D**) The levels of P-T37/46-4EBP were determined by immunofluorescence in HeLa cells and PKM2 overexpressing HeLa cells with and without rapamycin supplementation in the culture media, respectively. Bar scales are 25 μm. (**E**) EGF effects on the levels of endogenous P-T389-S6K and P-T37/46-4EBP of HEK293T cells and *PKM2* knockdown HEK293T cells were determined and quantified relative to that of non-treated cells. (**F**) *AKT1S1* was knocked down by *shRNA* in HEK293T cells (bottom). The levels of P-T389-S6K and P-T37/46-4EBP in response to PKM2 overexpression were determined in *AKT1S1* knockdown cells after *shRNA* resistant AKT1S1, 2SA and 2SE were each re-introduced into cells. P-T389-S6K and P-T37/46-4EBP signals were normalized relative to untreated cells. (**G**) Growth curves of HEK293T cells, the PKM2 overexpressing HEK293T cells, the *AKT1S1* knockdown HEK293T cells and PKM2 overexpressing *AKT1S1* knockdown HEK293T cells were determined. Shown are the average values (n = 3) with SD. Knockdown efficiency of *AKT1S1* is demonstrated in (**F**). **(H–J)** The levels of endogenous P-T389-S6K and P-T37/46-4EBP of HEK293T cells were detected under serum starvation (SS, H), amino acids starvation (AAs Starv., I) and both (**J**) were detected. Amino acids starvation was achieved by culturing cells in basal DMEM with all other ingredients except amino acids.

Because AKT1S1 competes binding sites with translation inhibitor eIF4E–binding protein (4EBP) on raptor^46^, the release of AKT1S1 from raptor by PKM2 would facilitate 4EBP binding to raptor. In line with these expectations, PKM2 overexpression in HeLa cells enhanced the raptor–4EBP1 interaction (Fig. 5A), and conversely, depletion of PKM2 in HeLa cells weakened the interaction between raptor and 4EBP1(Fig. 5B). These results are consistent with that the release of AKT1S1 from raptor activates mTORC1 signaling. Further support that PKM2 activates mTORC1 signaling, the endogenous phosphorylation levels of the eIF4E-binding protein (4EBP) on threonine 37 and threonine 46 (P-T37/46–4EBP) and the phosphorylation levels of the ribosomal protein S6 kinase (S6K) on T398 (P–T389–S6K), two well-established readouts of mTORC1 activation, were substantially elevated by PKM2 overexpression (Fig. 5C). Consistently, immunofluorescence analysis also showed that PKM2 overexpression increased P-T37/46–4EBP in HeLa cells (Fig. 5D). Moreover, although epidermal growth factor (EGF) is known to stimulate the protein kinase activity of PKM2^25^,^47^, knockdown of PKM2 abrogated EGF’s ability to activate mTORC1 signaling (Fig. 5E). This, together with that the activation of mTORC1 by PKM2 was abrogated by rapamycin, an mTORC1 inhibitor (Fig. 5C,D), further established an activator role of PKM2 to mTORC1. Furthermore, *shRNA* knockdown of AKT1S1 in HEK293T cells not only increased P-T389–S6K and P-T37/46–4EBP but also abrogated PKM2’s ability to further increase P-T389–S6K and P-T37/46–4EBP (Fig. 5F). Interestingly, compared with that reintroducing the phosphorylation mimetic 2SE mutant had negligible effect on the P-T389–S6K and P-T37/46–4EBP levels, reintroducing *shRNA*-resistant AKT1S1 and 2SA both decreased the P-T389–S6K and P-T37/46–4EBP levels, and remarkably, only reintroducing AKT1S1, but not 2SA, to *AKT1S1* knockdown cells restored PKM2’s ability to increase P-T389–S6K and P-T37/46–4EBP (Fig. 5F). These results further confirmed that PKM2 activates mTORC1 by phosphorylating S202/203 of AKT1S1. Lastly, although PKM2 overexpression and *AKT1S1* knockdown both accelerate the proliferation of HEK293T cells, *AKT1S1* knockdown abrogated the growth-promoting effects of PKM2 (Fig. 5G). Moreover, reintroducing *shRNA* resistant *AKT1S1* (Figure S4A), but not *shRNA* resistant *2SE* (Figure S4B), into the *AKT1S1* knockdown HEK293T cells restored PKM2’s ability to promote cell growth. These results further substantiate that phosphorylation on S202/203 of AKT1S1 by PKM2 activates mTORC1.

Activation of mTOR signaling usually requires nutritional and hormonal signals^7^. However, PKM2 overexpression alone activated mTOR signaling under serum starvation (Fig. 5H), amino acids starvation (Fig. 5I) or both (Fig. 5J). These results showed that PKM2 overexpression is sufficient to activate mTOR signaling, regardless the existence of growth factors and amino acids signals.

### PKM2 Inhibits Autophagy by Activating mTORC1

**Figure 6:**
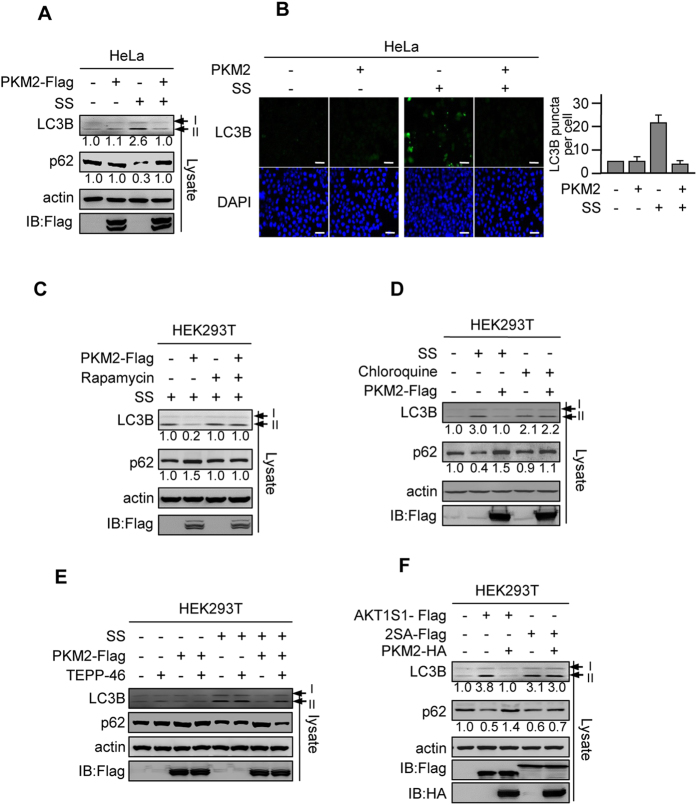
PKM2 inhibits autophagy by activating mTORC1. (**A**) Cells were cultured under either normal DMEM or serum starvation (SS) conditions. The levels of LC3B II and p62 in HeLa cells and HeLa cells expressing PKM2 were determined and normalized to those of untreated cells, respectively. (**B**) Cells were cultured under either normal DMEM or SS conditions. The LC3B levels in response to PKM2 overexpression were detected by immunofluorescence. Shown are representative immunofluorescence results (left) and average value of quantization of triplicate experiments with S.D.(right). Bar scales are 100 μm. (**C**) Under SS, the levels of LC3B and p62 in HEK293T cells expressing or not expressing PKM2 were compared in both the absence and presence of rapamycin. Signals of LC3B and p62 were quantified relative to those of neither PKM2 nor rapamycin was treated cells. (**D**) PKM2 was overexpressed in HEK293T cells cultured in SS and chloroquine (100 nM)-supplemented media. The PKM2 effects on the levels of LC3B and levels of p62 were determined and compared in each cells. Signals of LC3B and p62 were quantified relative to those of untreated cells. (**E**) Autophagy was induced by serum starvation in HEK293T cells, the effects of TEPP-46 on levels of LC3B and p62 were detected in HEK293T cells and in HEK293T cells overexpressing PKM2. TEPP-46 effects on autophagy markers were also examed in HEK293T cells without serum starvation as control. (**F**) The levels of LC3B and p62 of HEK293T cells were determined in the overexpressing of AKT1S1 or 2SA and co-expression of AKT1S1 and PKM2 or 2SA and PKM2. Signals of LC3B and p62 were quantified relative to those of untreated cells.

The activation of mTORC1 is known to prevent autophagy^48^,^49^, a catabolic pathway induced by harmful stimuli, including nutrient deprivation. Changes of markers of autophagosome formation, including a decrease of p62 and an increase in the conversion of LC3B-I to LC3B-II^50^,^51^, were prevented by PKM2 overexpression in HEK293T, HeLa, HCT116 ^*p53*+/+^ and HCT116 ^*p53*−/−^ cells cultured under serum starvation (Fig. 6A, S5A–C). These results were echoed by an immunofluorescence assay that revealed PKM2 overexpression in HeLa cells reversed serum starvation-induced LC3B overexpression (Fig. 6B), and showed that PKM2 overexpression prevents serum starvation-induced autophagy. Moreover, the autophagy-relieving effect of PKM2 was abolished by shutting down mTORC1 signaling with rapamycin (Fig. 6C), and chloroquine-induced mTORC1-independent autophagy was not responsive to PKM2 overexpression (Fig. 6D). These results suggested that PKM2 inhibits autophagy by mediating mTORC1 activity. Furthermore, consistent with that TEPP-46 abrogates PKM2’s protein kinase activity (see Fig. 3E), the autophagy preventive effects of PKM2 were abolished by TEPP-46 (Fig. 6E), confirmed that PKM2 prevents autophagy through its protein kinase activity. Lastly, the overexpression of AKT1S1, but not 2SA, induced autophagy in HeLa cells, and overexpression of PKM2 only rescued autophagy induced by AKT1S1 overexpression (Fig. 6F), showed that PKM2 inhibits autophagy through phosphorylating S202/203 of AKT1S1.

### Correlation of PKM2 Expression with AKT1S1 S202/203 Phosphorylation, mTOR Activation and Autophagy Inhibition in Cancers

**Figure 7:**
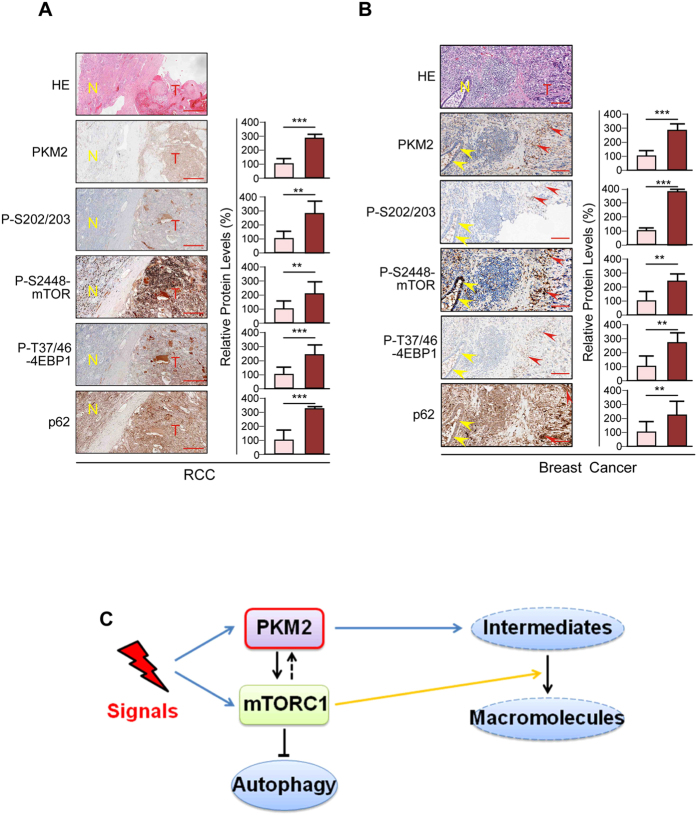
Correlation among PKM2 overexpression, S202/203 phosphorylation and mTORC1 activation in RCC and breast cancers. (**A,B**) PKM2, P-S202/203, P- S2448-mTOR, p-T37/46-4EBP and LC3B levels of the same patient were detected in RCC (**A**) and breast cancer (**B**) tissues and adjacent normal tissues. Representative IHC (left) and statistic (right, n = 10) results are shown. For RCC samples, normal and tumor tissues are marked by N and T, respectively. For breast cancer, tumor and normal tissues are marked by red and yellow arrows, respectively. Pathologic results were confirmed by experienced pathologists. Bar scales were 100 μm, heights of breast cancer samples were compressed to 1/2. (**C**) Schematic diagram of the PKM2-mTORC1 regulatory loop. Overexpression of PKM2 leads accumulation of anabolic intermediates and activation of mTOR signaling that promotes utilization of anabolic intermediates and inhibits autophagy. Meanwhile, mTOR activates PKM2 to form a positive loop to enhance the anabolic processes. Signals that activate either PKM2 or mTORC1 can result in both anabolic functions.

To confirm that PKM2 expression activates mTOR signaling and inhibits autophagy, we analyzed markers of each molecular events in renal cell carcinoma (RCC) and breast cancer samples. Immunohistochemistry (IHC) analysis was performed to 10 samples of each tumor that contained both normal and cancer tissues to show differential expression of each marker in normal and cancer tissues. In both RCC (Fig. 7A**, S6A**) and breast cancer (Fig. 7B**, S6B**), PKM2 was overexpressed in cancer tissues, alongside with elevated phosphorylation of S202/203, phosphorylation of mTOR and phosphorylation of 4EBP that signaled activation of mTOR signaling, and elevated p62 that signaled inhibited autophagy. These results provided evidence that PKM2 activates mTOR signaling and inhibits autophagy *in vivo*.

## Discussion

In the current study, we report as far as we acknowledged the first phosphorylome of PKM2, shed lights on the plethora functions of PKM2^52^. Our proteomic approach was validated by that substrates we identified overlapped some of the substrates previously identified by protein microarray assays^30^ (Table S1). However, by no mean our identifications were complete since some of the documented PKM2 substrates, such as T11 of H3^27^ and Y705 of stat3^29^, were not identified by the current survey. Nevertheless, close to 70% of the PKM2 substrates are located in the nucleus (Fig. 2D) and PKM2 substrates are enriched in various cancer pathways (Fig. 2F), consistent with the fact that the protein kinase activity of PKM2 plays critical roles in cancer biology. Interestingly, although no consensus sequence was identified for PKM2, the phosphorylation site preference of PKM2 is similar to that of Casein Kinase II (CK2), a conserved protein serine/threonine kinase that preferentially phosphorylates serine and threonine sites spanned by acidic amino acids^33^,^35^, suggested that PKM2 may, like CK2, may play a plethora of roles in proliferation, apoptosis, differentiation, transformation and carbohydrate metabolism regulation^53^,^54^.

The finding that PKM2 phosphorylates S202/203 of AKT1S1 revealed a linkage between PKM2 overexpression and mTOR activation in cancer cells. Previous phosphorylomic studies had repeatedly found that Ser202 and Ser203 are phosphorylated by unidentified upstream kinases^55^,^56^,^57^,^58^. The biologic significance of S202/203 phosphorylation was therefore uncharacterized. Our study showed that S202/203 of AKT1S1 are phosphorylated by PKM2 and their phosphorylation activates mTORC1 by relieving AKT1S1 from raptor. Importantly, mTOR signaling activation by PKM2 is independent of nutritional signals (Fig. 5H–J). This relieved cancer cells from restrains of nutrients and hormonal signals that are subject to fluctuate, ensures constant growth/proliferation advantages for them over normal cells. Moreover, activation of mTORC1 by PKM2 highlights the how PKM2 overexpression promotes cancerous metabolism. PKM2 promotes accumulation of glycolytic intermediates for biosynthesis^17^ as well as activates the mTOR signaling, which utilizes intermediates for macromolecules biosynthesis^4^. PKM2 thus simulates those of mutations of tumor suppressor p53 or oncogene c-Myc, which not only promote aerobic glycolysis to accumulate glycolytic intermediates^59^,^60^ but also activate anabolic processes^61^,^62^. This, together with that mTOR activation was reportedly to increase PKM2 expression^63^, suggests that there exist a positive regulatory loop between mTORC1 and PKM2. When either PKM2 is overexpressed or mTOR signaling is activated, PKM2 expression and mTOR signaling are concomitantly activated through this loop. Therefore, when either PKM2 or mTORC1 are activated, both anabolic intermediates accumulation and utilization and autophagy inhibition will be resulted (Fig. 7C). All these effects facilitate the onset of cancers.

In RCC and breast cancer samples, strong correlations had been found among PKM2 overexpression, S202/203 phosphorylation, mTOR activation and autophagy inhibition (Fig. 7), consistent with that PKM2 overexpression and mTOR activation are both exist in RCC and breast cancers^5^,^64^,^65^ and support that PKM2 activates mTOR signaling *in vivo*. Given that PKM2 expression and its protein kinase activity is involved in cell cycle regulation^66^, inhibiting protein kinase activity of PKM2 may represent a mean to curb anabolic metabolism and cancer progression.

## Materials and Methods

### Cell culture, materials and antibodies

HEK293T and HeLa cells were cultured in DMEM containing 10% NCS. HCT116^+/+^ and HCT116^−/−^ were cultured in McCoy′5A containing 10% FBS. Cells were transfected with plasmids using polyethylenimine (PEI, linear, 25KDa). AKT1S1 S202A, S202E, S203A and S203E were cloned into PRK7-Flag, PKM2 and K270M was cloned into pcDNA3.1-Flag, pcDNA3.1-Myc and pcDNA3.1-HA, respectively.

Chloroquine diphosphate salt (c6628-25 g) was from Sigma-aldrich, TEPP46 was from Cayman. Antibodies were either home-made or commercially purchased.

### *In Vitro* phosphorylation and dephosphorylation

The recombinant Flag-PKM2, His-PKM2 and its mutant were affinity purified from HEK293T cells overexpressing pcDNA3.1-Flag-PKM2 and from *E. Coli* BL21(DE3)plysS overexpressing pET22b-His-PKM2, respectively. Proteins or proteome of HEK293T were incubated with 1 mg/ml PKM2 in kinase buffer contains 100 mM KCl, 50 mM MgCl_2_, 1 mM DTT, 1 mM NaVO_4_, 5% glycerol, 30 mM HEPES (pH 7.6) at 30 °C for 30 minutes. For *in vitro* dephosphorylation, proteins or proteome were incubated in dephosphorylation buffer that contains 50U alkaline phosphatase (Sigma), 1 mM MgCl2, 1 mM ZnCl2, 50 mM HEPES (pH 7.5) at 30 °C for 120 minutes.

### Quantitative phosphorylomic analysis

Tryptic peptides of PKM2 treated and untreated proteome of HEK293T cells were heavy and light labeled, respectively, as described in text. The Ti^4+^ -IMAC microspheres were used to enrich the phosphopeptides following the reported protocol. For mass spectrometric analysis, a quaternary surveyor MS pump (Thermo, San Jose, CA) coupled with a LTQ Orbitrap XL mass spectrometer (Thermo, CA) was used. Data analysis and detailed parameters are described in the supplementary information. Phosphorylation levels increased more than twofold by PKM2 are selected as candidate substrates of PKM2.

### Western blot, dot blot and immunofluorescence analysis

Standard procedures were followed for western blot, dot blot and Immunofluorescence analysis. Western blot signals, dot blot signals were obtained by detecting chemiluminescence on Typhoon FLA 9500 (GE Healthcare). Scoring of Immunofluorescence of LC3B was analyzed using ANOVA with Turkey’s post-test (One-way ANOVA for comparisons between groups, Two-way ANOVA for comparisons of magnitude of changes between different groups from different cell lines).

### Bioinformatics analysis

All analysis was performed using published software. The gene ontology annotations were downloaded from QuickGO database, while the enrichment analyses were performed with a hypergeometric distribution. The enrichment analyses of KEGG pathways were carried out using DAVID. All the heatmaps were visualized with the ggplot2 program (http://had.co.nz/ggplot2/) in the R package (http://www.r-project.org/). The amino acid preferences were visualized with WebLogo, while the comparisons of amino acid preferences were visualized with Two Sample Logo.

### Human Samples and Mutation Screening

Human samples are acquired from Ruijing Hospital, Shanghai Jiaotong University. Informed consents from the patients were obtained. The procedures related to human subjects were carried out in accordance with the approved guidelines approved by Ethic Committees of Shanghai Jiaotong University.

### Immunohistochemistry

Tissue sections were prepared form the formalin-fixed paraffin embedded specimens. Antigen retrieval of renal cell carcinoma or breast cancer specimens was performed by incubating the slides in Tris-EDTA buffer (pH 8.4) at 99 °C for 60 minutes. The endogenous peroxidase activity was inactivated in solution of methanol with 3% H2O2. The slides were incubated with primary antibody for 60 minutes and secondary antibody for 8 minutes, followed by DAB Chromagen stainning for 8 minutes. All procedures were performed using stainer (BenchMark XT, Ventana) and the slides were scanned by scanner (Ventana iScan Coreo). The quantification of IHC results were performed by an experienced pathologist. The intensity was calculated according to positive areas and positive degree. Sections were staining with PKM2 (1:100), P-S202/203-AKT1S1 (1:30), P-S2448-mTOR (1:100), P-T37/46-4EBP1 (1:500), p62 (1:200) and LC3B (1:100) antibody using an Ultraview Detection Kit.

### Statistical Analysis

Significant differences between groups were determined using Student’s T test. The significance level for statistical testing was set at p < 0.05.

## Additional Information

**How to cite this article**: He, C.-L. *et al.* Pyruvate Kinase M2 Activates mTORC1 by Phosphorylating AKT1S1. *Sci. Rep.* **6**, 21524; doi: 10.1038/srep21524 (2016).

## Supplementary Material

Figure S1-6 Table S1-2
