# Supplementary material for: Pyruvate Kinase M2 Activates mTORC1 by Phosphorylating AKT1S1
Source: Sci Rep. 2016 Feb 15;6:21524. doi: 10.1038/srep21524 (PMC4753445; doi:10.1038/srep21524)
Supplement: Figure S1-6 Table S1-2 [file srep21524-s1.pdf]

Supplementary Information

Pyruvate Kinase M2 Activates mTORC1 by Phosphorylating AKT1S1

Chang-Liang He, Yang-Yang Bian, Yu Xue, Ze-Xian Liu, Kai-Qiang Zhou, Cui-Fang Yao, Yan Lin, Han-Fa Zou, Fang-Xiu Luo, Yuan-Yuan Qu, Jian-Yuan Zhao, Ming-Liang Ye, Shi-Min Zhao and Wei Xu

Supplementary Data

Supplementary Figures S1-S6

Figure S1

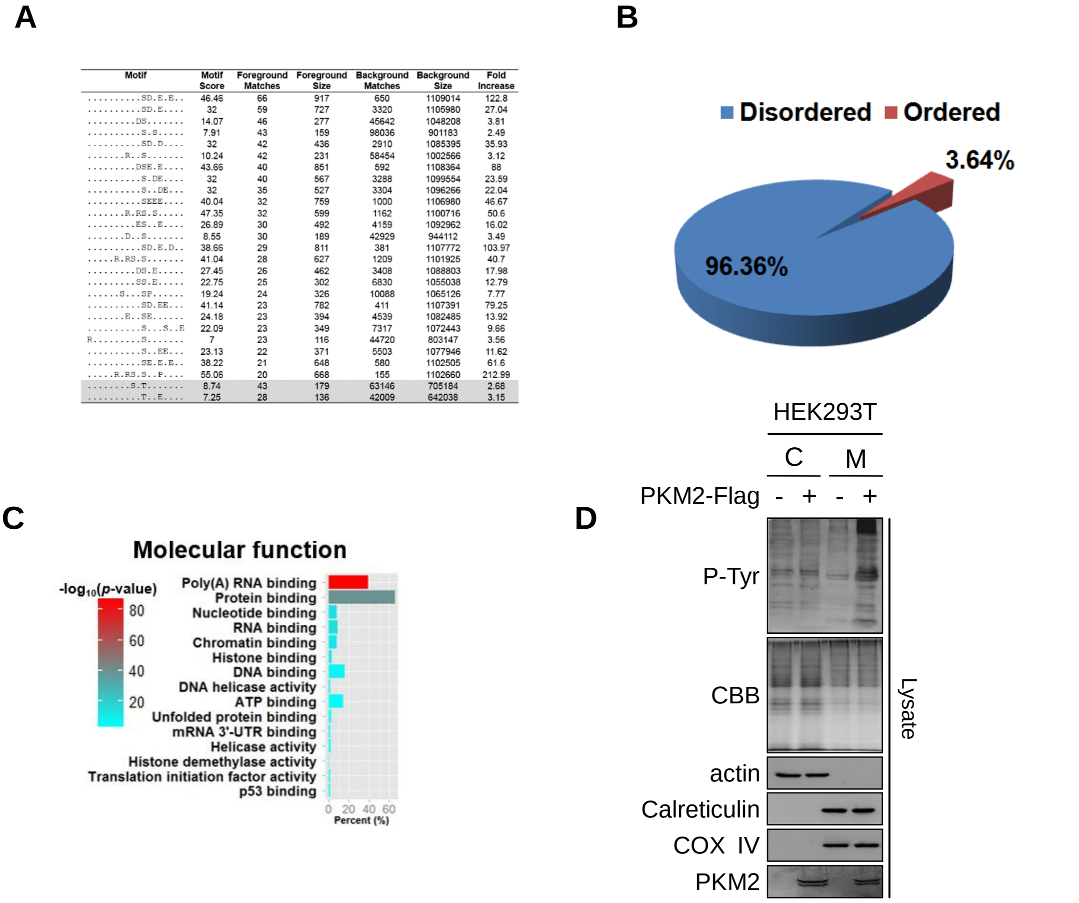

Motif	Motif Score	Foreground Matches	Foreground Size	Background Matches	Background Size	Fold Increase
..........SD.E.E..	46.46	66	917	650	1109014	122.8
..........SD.E....	32	59	727	3320	1105980	27.04
.........DS.......	14.07	46	277	45642	1048208	3.81
..........S.S.....	7.91	43	159	98036	901183	2.49
..........SD.D....	32	42	436	2910	1085395	35.93
.......R..S.......	10.24	42	231	58454	1002566	3.12
.........DSE.E....	43.66	40	851	592	1108364	88
..........S.DE....	32	40	567	3288	1099554	23.59
..........S..DE...	32	35	527	3304	1096266	22.04
..........SEEE....	40.04	32	759	1000	1106980	46.67
.......R.RS.S.....	47.35	32	599	1162	1100716	50.6
.........ES..E....	26.89	30	492	4159	1092962	16.02
.......D..S.......	8.55	30	189	42929	944112	3.49
..........SD.E.D..	38.66	29	811	381	1107772	103.97
.....R.RS.S.......	41.04	28	627	1209	1101925	40.7
.........DS.E.....	27.45	26	462	3408	1088803	17.98
.........SS.E.....	22.75	25	302	6830	1055038	12.79
......S...SP......	19.24	24	326	10088	1065126	7.77
..........SD.EE...	41.14	23	782	411	1107391	79.25
.......E..SE......	24.18	23	394	4539	1082485	13.92
..........S...S..E	22.09	23	349	7317	1072443	9.66
R.........S.......	7	23	116	44720	803147	3.56
..........S..EE...	23.13	22	371	5503	1077946	11.62
..........SE.E.E..	38.22	21	648	580	1102505	61.6
.....R.RS.S..P....	55.06	20	668	155	1102660	212.99
........S.T.......	8.74	43	179	63146	705184	2.68
..........T..E....	7.25	28	136	42009	642038	3.15

Supplementary Figure S1, Bioinformatics Analysis of Substrates of PKM2 (Related to Figure 2). Bioinformatics analysis results are shown. **A**, Motif-x analysis was employed to identify consensus sequences in substrates (S and T) of PKM2. **B**, The distribution of phosphorylation sites of PKM2 in ordered and disordered regions of proteins. **C**, KEGG molecular functions enrichment of substrates of PKM2. **D**, PKM2 increases membrane-bound P-Tyr. Flag-tagged PKM2 was overexpressed in HEK293T cells. The levels of P-Tyr in cytosolic (C) and membrane (M) fractions were determined.

Figure S2

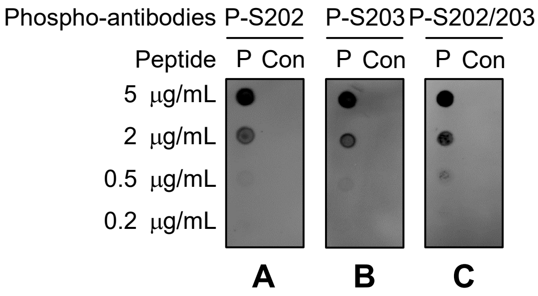

Supplementary Figure S2, Validation of P-202, P-203 and P-202/203 antibodies (Related to Figure 3). Dot blot assays were carried out to determine the specificities of AKT1S1 P-S202 (**A**), P-S203 (**B**) and P-S202/203 (**C**) antibodies. Anti-sera were used at 1:1000 dilutions. Synthetic phosphor-serine containing peptides (P) that was used in making each antibody and the control peptides (Con) that have the same amino acids sequence but devoid of phosphor-serines were fixed onto nitrocellulose membrane at the concentrations as indicated, the signal of antibodies reactive to each antigen and to each control peptide was detected by chemiluminescence on Typhoon (GE).

Figure S3

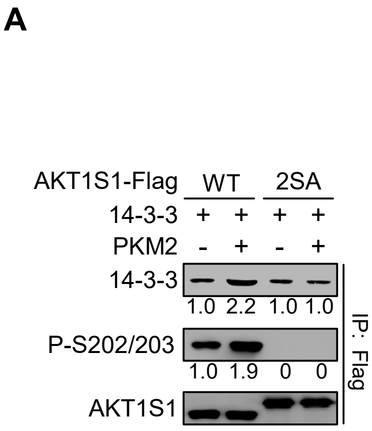

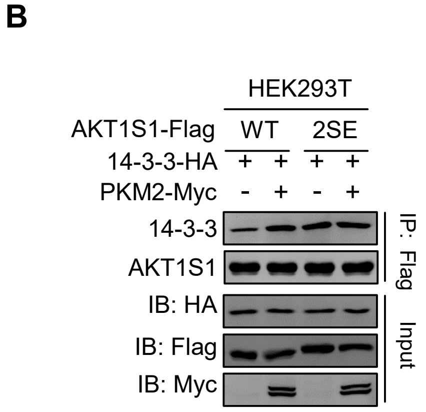

Supplementary Figure S3, S202/203 phosphorylation increases AKT1S1-14-3-3 interaction (Related to Figure 4). A, Purified 14-3-3 was incubated with AKT1S1 or 2SA with or without PKM2 *in vitro*. After reaction, the levels of P-S202/203 of Flag beads purified AKT1S1 or 2SA and the amount of 14-3-3 pulled down by purified AKT1S1 or 2SA was determined (western blot) and quantified (numeric). **B**, 14-3-3 was co-expressed with either AKT1S1 or 2SE in HEK293T cells, the amount of 14-3-3 co-immunoprecipitated with either AKT1S1 or 2SE with expressing PKM2 or without expressing PKM2 were determined.

Figure S4

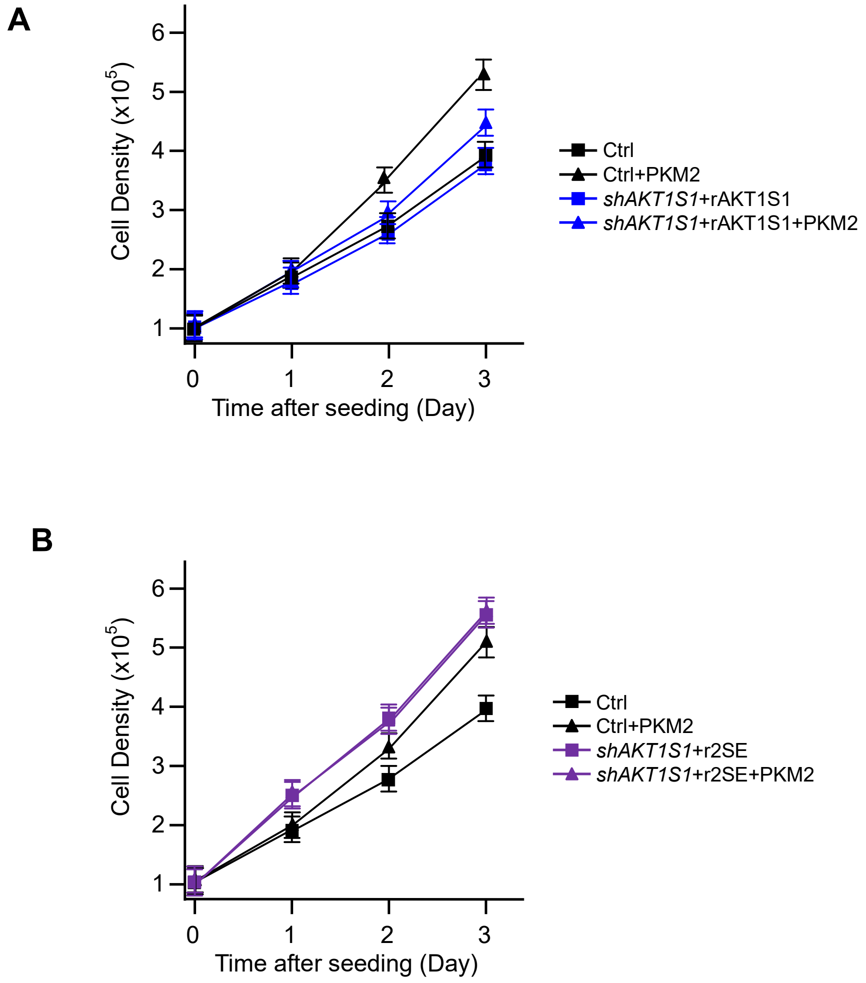

Supplementary Figure S4, PKM2 activates mTORC1 (Related to Figure 5).

Growth curves of AKT1S1 knockdown HEK293T cells that stably overexpressing *shRNA* resistant AKT1S1 (rAKT1S1) (**A**) or 2SE (r2SE) (**B**) were detected with and without overexpressing PKM2. Growth curves of HEK293T cells with and without overexpressing PKM2 were determined in each experiment as controls.

Figure S5

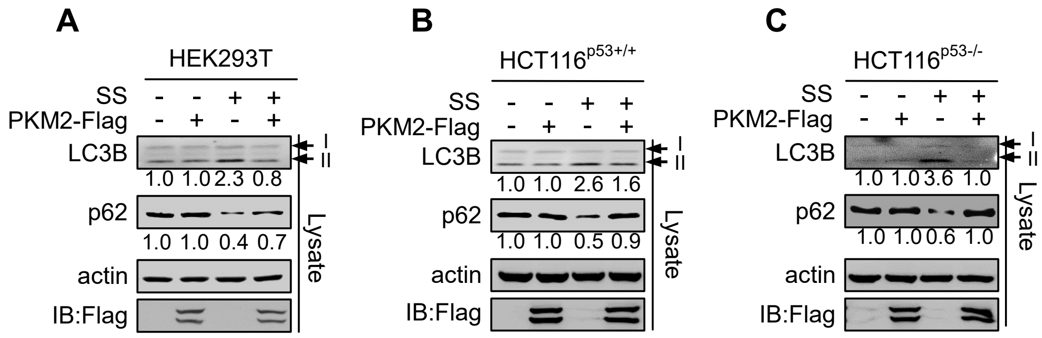

Supplementary Figure S5, PKM2 overexpression prevented serum starvation induced autophagy in HEK293T, HCT116$^{p53+/+}$ and HCT116$^{p53-/-}$ cells (Related to Figure 6). Cells were cultured under either normal DMEM or serum starvation (SS) conditions. The levels of LC3B and p62 in HEK293T cells and HEK293T cells expressing PKM2 **(A)**, in HCT116$^{P53+/+}$ cells and HCT116$^{P53+/+}$ cells expressing PKM2 **(B)**, in HCT116$^{P53-/-}$ cells and HCT116$^{P53-/-}$ cells expressing PKM2 **(C)**, were determined and quantified.

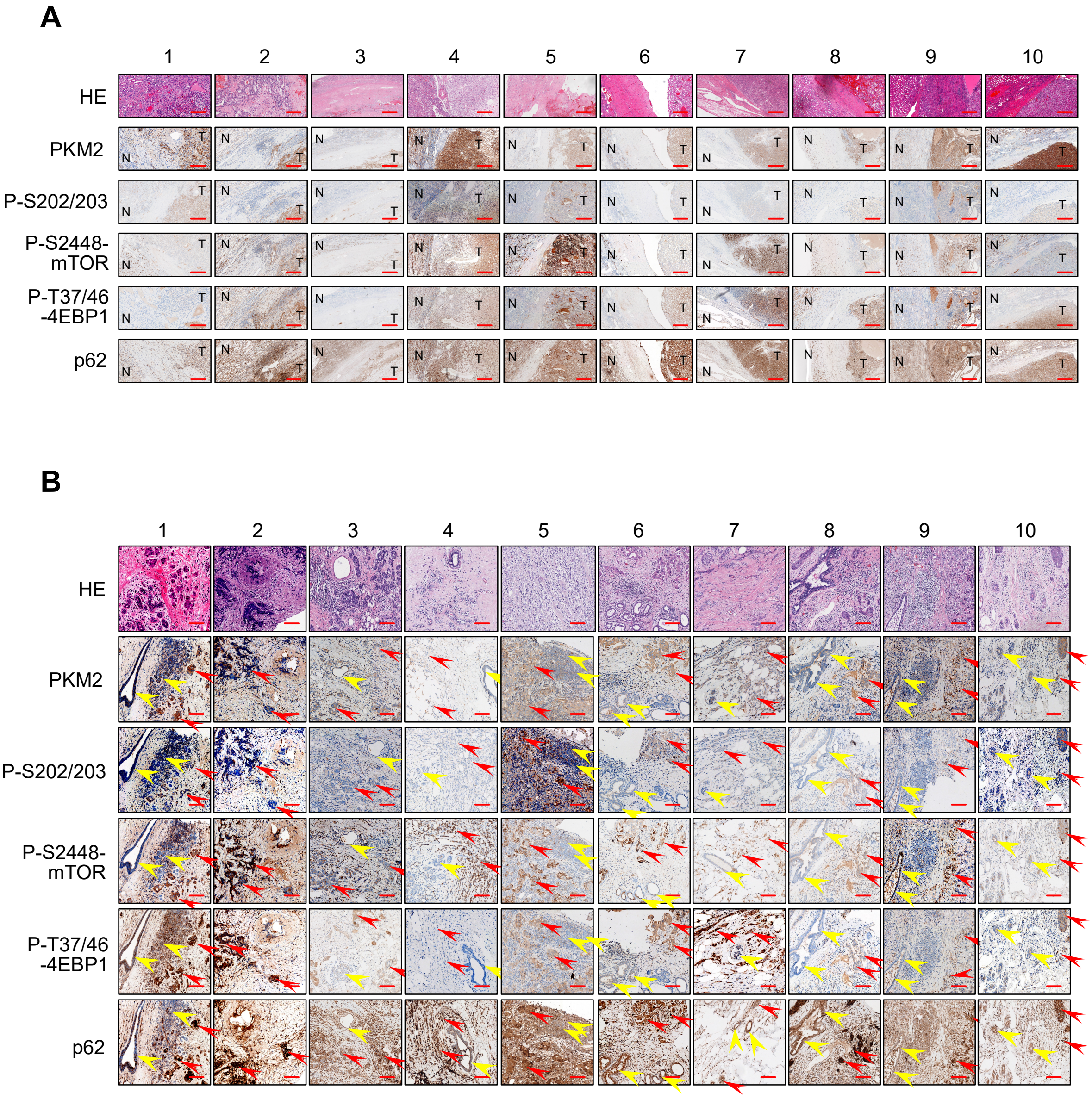

Supplementary Figure S6, IHC analysis of RCC and breast cancer samples (Related to Figure 7). PKM2, P-S202/203, P- S2448-mTOR, p-T37/46-4EBP and p62 levels of the same patient were detected in RCC (A) and breast cancer (B) tissues and adjacent normal tissues. For RCC samples, normal and tumor tissues are marked by N and T, respectively. For breast cancer, tumor and normal tissues are marked by red and yellow arrows, respectively. Pathologic results were confirmed by experienced pathologists. Bar scales were 100 μm.

Supplemental Table S1

Table S1: Identified PKM2 Substrates

	UniProt Acces	Gene name	Position	Code	Peptide Sequence	Fold Change
1	O75643	SNRNP200	225	S	EEGDEDVYGEVREEASDDDMEGDEAVVRCTL	3166.18
2	Q96EV2	RBM33	205	S	GGMETLELQKDIKEESDEEEEDDEESGRLRF	2576.20
3	O95400	CD2BP2	49	S	SGGPGSRFKGKHSLDSDEEEDDDDGGSSKYD	1828.91
4	Q8WVC0	LEO1	197	S	DEKMQNTDDEERPQLSDDERQQLSEEEKANS	1769.91
5	Q96ST2	IWS1	196	S	QISDSESEEPPRHQASDSENEEPPKPRMSDS	1769.83
6	P25205	MCM3	711	S	QPDAKDGDSYDPYDFSDTEEEMPQVHTPKTA	1415.93
7	Q96ST2	IWS1	235	S	QVSDSESEEPPRHQASDSENEELPKPRISDS	1356.93
8	Q96ST2	IWS1	198	S	SDSESEEPPRHQASDSENEEPPKPRMSDSES	1317.60
9	Q96ST2	IWS1	237	S	SDSESEEPPRHQASDSENEELPKPRISDSES	1254.30
10	Q12872	SFSWAP	870	S	RSRSRTKSKARSQSVSPSKQAAPRPAAPAAH	1163.40
11	Q13435	SF3B2	436	S	KKGFEEEHKDSDDDSSDDEQEKKPEAPKLSK	1144.61
12	O60841	EIF5B	214	S	GQKKNQKNKPGPNIESGNEDDDASFKIKTVA	1140.61
13	Q13435	SF3B2	435	S	KKKGFEEEHKDSDDDSSDDEQEKKPEAPKLS	1061.95
14	Q96ST2	IWS1	248	S	QASDSENEELPKPRISDSESEDPPRHQASDS	983.28
15	Q4G0J3	LARP7	261	S	TSISKMKRSRPTSEGSDIESTEPQKQCSKKK	964.25
16	P52701	MSH6	227	S	TTYVTDKSEEDNEIESEEEVQPKTQGSRRSS	943.95
17	Q9UBB9	TFIP11	98	S	AGLKKGAAEEAELEDSDDEEKPVKQDDFPKD	865.29
18	Q5C9Z4	NOM1	320	S	GKRVRFAEDEEKSENSSEDGDITDKSLCGSG	814.29
19	Q9UQ35	SRRM2	1925	S	RSRSRASPVSRRRSRSRTPPVTRRRSRSRTP	806.29
20	P42166	TMPO	66	S	PPLPAGTNSKGPPDFSSDEEREPTPVLGSGA	786.95
21	Q9H0D6	XRN2	499	S	GSPSPLGGIKRKAEDSDSEPEPEDNVRLWEA	786.63
22	O95218	ZRANB2	188	S	EDEDDADLSKYNLDASEEEDSNKKKSNRRSR	781.63
23	Q13428	TCOF1	277	S	RAKKPEEESESSEEGSESEEEAPAGTRSQVK	766.96
24	P05455	SSB	366	S	GKGKVQFQGKKTKFASDDEHDEHDENGATGP	747.30
25	P48634	PRRC2A	350	S	YTEKLKFSDEEDGRDSDEEGAEGHRDSQSAS	727.63
26	Q5C9Z4	NOM1	317	S	KRRGKRVRFAEDEEKSENSSEDGDITDKSLC	707.96
27	Q13247	SRSF6	303	S	DIKSKSRSRSQSRSNSPLPVPPSKARSVSPP	688.51
28	Q9H0G5	NSRP1	248	S	ILQTDVKVEENPDADSDFDAKSSADDEIEET	688.30
29	Q9HCN4	GPN1	338	S	ILTRGTLDEEDEEADSDTDDIDHRVTEESHE	684.34
30	O43719	HTATSF1	600	S	ENVLDKELEENDSENSEFEDDGSEKVLDEEG	648.97
31	O43719	HTATSF1	624	S	KVLDEEGSEREFDEDSDEKEEEEDTYEKVFD	629.30
32	O43719	HTATSF1	597	S	ELHENVLDKELEENDSENSEFEDDGSEKVLD	625.58
33	P35659	DEK	32	S	PASEKEPEMPGPREESEEEEDEDDEEEEEEE	609.64
34	Q5VT52	RPRD2	374	S	PEPVTDNRDVEDMELSDVEDDGSKIIVEDRK	609.27
35	P25205	MCM3	672	S	VLEKEKKRKKRSEDESETEDEEEKSQEDQEQ	571.30
36	P49756	RBM25	703	S	KLPVDSVFNKFEDEDSDDVPRKRKLVPLDYG	570.83
37	P51116	FXR2	453	S	TGGPAYGPSSDVSTASETESEKREEPNRAGP	570.30
38	O60231	DHX16	103	S	RALLEKNRSYRLLEDSEESSEETVSRAGSSI	570.16
39	P52701	MSH6	261	S	KKRRVISDSESDIGGSDVEFKPDTKEEGSSD	550.71
40	P14625	HSP90B1	306	S	EPMEEEEAAKEEKEESDDEAAVEEEEEEKKP	550.67
41	Q9H3N1	TMX1	247	S	LKKVEEEQEADEEDVSEEEAESKEGTNKDFP	550.64
42	Q8WVC0	LEO1	300	S	RMKRKNAIASDSEADSDTEVPKDNSGTMDLF	550.28
43	O43719	HTATSF1	642	S	KEEEEDTYEKVFDDESDEKEDEEYADEKGLE	530.97
44	P51532	SMARCA4	695	S	PTLPVEEKKKIPDPDSDDVSEVDARHIIENA	512.30
45	Q13435	SF3B2	431	S	SAAPKKKGFEEEHKDSDDDSSDDEQEKKPEA	511.75
46	O00505	KPNA3	60	S	LLKKRNVPQEESLEDSDVDADFKAQNVTLEA	511.31
47	P24534	EEF1B2	106	S	GATDSKDDDDIDLFGSDDEEESEEAKRLREE	493.24
48	P52701	MSH6	274	S	GGSDVEFKPDTKEEGSSDEISSGVGDSESEG	491.92
49	Q8WVC0	LEO1	294	S	SEDEVLRMKRKNAIASDSEADSDTEVPKDNS	491.64
50	Q8WVC0	LEO1	296	S	DEVLRMKRKNAIASDSEADSDTEVPKDNSGT	491.28
51	Q9H0G5	NSRP1	255	S	VEENPDADSDFDAKSSADDEIEETRVNCRRE	472.21
52	O95218-2	ZRANB2	305	S	SRSSSSGDRKKRRTRSRSPESQVIGENTKQP	471.98
53	Q6QNY0	BLOC1S3	65	S	RPTGLRVAGEAAETDSEPEPEPEPTAAPRDL	471.69
54	Q99856	ARID3A	88	S	GHPASPGGSEDGPPGSEEEDAAREGTPGSPG	471.42
55	O95671	ASMTL	239	S	VKHDSIPAADTFEDLSDVEGGGSEPTQRDAG	462.51

56	P49756	RBM25	583	S	EAERRRQPQIKQEPESEEEEEEKQEKEEKRE	453.25
57	P55081	MFAP1	116	S	LARHRKIVEPEVVGESDSEVEGDAWRMERED	452.72
58	Q86UE4	MTDH	426	S	SLLKSQEPIPDDQKVSDDDKEKGEGALPTGK	452.57
59	Q9NTJ3	SMC4	28	S	RREEGPPPPSPDGASSDAEPEPPSGRTESPA	452.47
60	Q9UEG4	ZNF629	28	S	QGPEQSPNDAHRGAESENEEESPRQESSGEE	452.31
61	P67809	YBX1	313	S	QDGKETKAADPPAENSSAPEAEQGGAE****	432.83
62	Q8NHM5	KDM2B	975	S	RTENSLANENQQPIKSEPESEGEEPKRPPGI	432.79
63	Q96EV2	RBM33	41	S	AERSWRRRAADEDWDSELEDDLLGEDLLSGK	432.65
64	Q9NTJ3	SMC4	27	S	RRREEGPPPPSPDGASSDAEPEPPSGRTESP	432.57
65	P13861	PRKAR2A	78	S	PPEPGPDRVADAKGDSESEEDEDLEVPVPSR	432.14
66	P35269	GTF2F1	307	S	PQQEEGPKGVDEQSDSSEESEEEKPPEEDKE	431.73
67	P52701	MSH6	252	S	GSRRSSRQIKKRRVISDSESDIGGSDVEFKP	413.31
68	P52701	MSH6	256	S	SSRQIKKRRVISDSESDIGGSDVEFKPDTKE	413.14
69	P55209	NAP1L1	10	S	******MADIDNKEQSELDQDLDDVEEVEEE	412.98
70	Q9BW71	HIRIP3	223	S	AKKVEGNKGTKSLKESEQESEEEILAQKKEQ	412.87
71	O43719	HTATSF1	616	S	EFEDDGSEKVLDEEGSEREFDEDSDEKEEEE	393.82
72	O60293	ZFC3H1	28	S	GLSPKEEGELEDGEISDDDNNSQIRSRSSSS	393.74
73	Q6P6C2	ALKBH5	64	S	EPYPVSGAKRKYQEDSDPERSDYEEQQLQKE	393.62
74	Q8IWX8	CHERP	817	S	SKSYSPGRRRRSRSRSPTPPSSAGLGSNSAP	393.58
75	Q8N1G4	LRRC47	520	S	EMKKYTLENKEEGSLSDTEADAVSGQLPDPT	393.49
76	Q9H0G5	NSRP1	254	S	KVEENPDADSDFDAKSSADDEIEETRVNCRR	393.31
77	Q7KZ85	SUPT6H	75	S	DDDDEDEGEEDEGSDSGDSEDDVGHKKRKRT	393.28
78	P55081	MFAP1	118	S	RHRKIVEPEVVGESDSEVEGDAWRMEREDSS	373.81
79	O43719	HTATSF1	714	S	DDDSNEKLFDEEEDSSEKLFDDSDERGTLGG	373.72
80	O76031	CLPX	617	S	YIRAPTKESSEEEYDSGVEEEGWPRQADAAN	373.67
81	P05388	RPLP0	307	S	AAAPAKVEAKEESEESDEDMGFGLFD*****	373.65
82	Q13428	TCOF1	87	S	QAKKTRVSDPISTSESSEEEEEAEAETAKAT	373.58
83	Q13442	PDAP1	60	S	DGAAGDPKKEKKSLDSDESEDEEDDYQQKRK	373.47
84	Q14527	HLTF	397	S	SSRPKRRKTAVQYIESSDSEEIETSELPQKM	373.39
85	Q7KZ85	SUPT6H	78	S	DEDEGEEDEGSDSGDSEDDVGHKKRKRTSFD	367.15
86	Q8IY81	FTSJ3	335	S	AKKLKEQAKALDISLSSGEEDEGDEEDSTAG	357.40
87	Q8IY81	FTSJ3	336	S	KKLKEQAKALDISLSSGEEDEGDEEDSTAGT	354.31
88	Q8TDD1	DDX54	782	S	LYQKWKQKQKIDDRDSDEEGASDRRGPERRG	354.27
89	Q9H6Z4	RANBP3	353	S	NEVSSDANRENAAAESGSESSSQEATPEKES	354.16
90	Q9UJV9	DDX41	21	S	PERKRARTDEVPAGGSRSEAEDEDDEDYVPY	354.14
91	Q9UNS1	TIMELESS	1173	S	KEPLKAAPKKRQLLDSDEEQEEDEGRNRAPE	353.98
92	Q9BW71	HIRIP3	227	S	EGNKGTKSLKESEQESEEEILAQKKEQREEE	353.94
93	Q13428	TCOF1	88	S	AKKTRVSDPISTSESSEEEEEAEAETAKATP	353.88
94	Q13308	PTK7	1053	S	RLMQRCWALSPKDRPSFSEIASALGDSTVDS	353.86
95	O43719	HTATSF1	721	S	LFDEEEDSSEKLFDDSDERGTLGGFGSVEEG	353.82
96	O60841	EIF5B	182	S	DEDNSKKIKERSRINSSGESGDESDEFLQSR	353.74
97	P13861	PRKAR2A	80	S	EPGPDRVADAKGDSESEEDEDLEVPVPSRFN	353.69
98	Q7KZ85	SUPT6H	73	S	INDDDDEDEGEEDEGSDSGDSEDDVGHKKRK	353.57
99	Q8TF01	PNISR	290	S	EGGDGPRLPQRSKFDSDEEEEDTENVEAASS	353.48
100	Q9P2I0	CPSF2	419	S	EAAKKLEQSKEADIDSSDESDIEEDIDQPSA	351.21
101	Q9UHB7	AFF4	222	S	HQRSKSPRDPDANWDSPSRVPFSSGQHSTQS	347.61
102	Q9UJV9	DDX41	23	S	RKRARTDEVPAGGSRSEAEDEDDEDYVPYVP	335.60
103	Q9UQ35	SRRM2	497	S	GRSRSPATAKRGRSRSRTPTKRGHSRSRSPQ	334.93
104	Q9Y2W1	THRAP3	379	S	QTNTDKEKIKEKGSFSDTGLGDGKMKSDSFA	334.84
105	O14654	IRS4	775	S	KAPDTNKEDDSKDNDSESDYMFMAPGAGAIP	334.78
106	O43719	HTATSF1	676	S	KKAEEGDADEKLFEESDDKEDEDADGKEVED	334.67
107	O60524	NEMF	747	S	QSGRDELNEELIQEESSEDEGEYEEVRKDQD	334.51
108	Q8WXI9	GATAD2B	129	S	PERGRLTPSPDIIVLSDNEASSPRSSSRMEE	334.47
109	Q9BWU0	SLC4A1AP	312	S	KQQQILLEKKMLGEDSDEEEEMDTSERKINA	334.38
110	Q92733	PRCC	267	S	AALQVTKQITQEEDDSDEEVAPENFFSLPEK	334.37
111	Q9UQ35	SRRM2	1729	S	PETRSRTPPRHRRSPSVSSPEPAEKSRSSRR	334.36
112	O60841	EIF5B	186	S	SKKIKERSRINSSGESGDESDEFLQSRKGQK	334.32

113	Q8NHM5	KDM2B	979	S	SLANENQQPIKSEPESEGEEPKRPPGICERP	334.28
114	P46821	MAP1B	1016	S	EEDMDEAIEKGEAEQSEEEADEEDKAEDARE	334.15
115	Q13428	TCOF1	769	S	VKAEKQEDSESSEEESDSEEAAASPAQVKTS	314.93
116	Q5T8D3	ACBD5	196	S	TPNAKTVNGKAESSDSGAESEEEEAQEEVKG	314.88
117	Q8NAV1	PRPF38A	193	S	EPRVSALEEDMDDVESSEEEEEEDEKLERVP	314.82
118	Q9Y2W2	WBP11	353	S	AGQEIPEEGREVEEFSEDDDEDDSDDSEAEK	314.73
119	Q9P2I0	CPSF2	423	S	KLEQSKEADIDSSDESDIEEDIDQPSAHKTK	314.67
120	Q13308	PTK7	1064	S	KDRPSFSEIASALGDSTVDSKP*********	314.65
121	Q14527	HLTF	400	S	PKRRKTAVQYIESSDSEEIETSELPQKMKGK	314.63
122	P21127	CDK11B	47	S	IKRLKNSDDRDSKRDSLEEGELRDHRMEITI	314.50
123	P22059	OSBP	351	S	VGSGKDQCCSGKGDMSDEDDENEFFDAPEII	311.40
124	P34741	SDC2	115	S	QTKSPEETDKEKVHLSDSERKMDPAEEDTNV	298.34
125	Q14684	RRP1B	452	S	KALKARVAEPGAEATSSTGEESGSEHPPAVP	295.25
126	Q9H307	PNN	347	S	EEAGEEEEKEIAIVHSDAEKEQEEEEQKQEM	295.15
127	Q9NWV8	BABAM1	49	S	AEDRAVGAQASVGSRSEGEGEAASADDGSLN	294.99
128	Q9UQ35	SRRM2	1541	S	KTVARTPLGQRSRSGSSQELDVKPSASPQER	294.84
129	Q9P2I0	CPSF2	420	S	AAKKLEQSKEADIDSSDESDIEEDIDQPSAH	294.73
130	P35269	GTF2F1	308	S	QQEEGPKGVDEQSDSSEESEEEKPPEEDKEE	294.35
131	Q8IY81	FTSJ3	599	S	SPLYQDEAPKGTEASSGTEAATGLEGEEKDG	287.34
132	O95218-2	ZRANB2	307	S	SSSSGDRKKRRTRSRSPESQVIGENTKQP**	284.50
133	Q14527	HLTF	398	S	SRPKRRKTAVQYIESSDSEEIETSELPQKMK	275.72
134	O60231	DHX16	106	S	LEKNRSYRLLEDSEESSEETVSRAGSSLQKK	275.68
135	Q13308	PTK7	1055	S	MQRCWALSPKDRPSFSEIASALGDSTVDSKP	275.53
136	O15541	RNF113A	84	S	KTRDSGKQKAAYGDLSSEEEEENEPESLGVV	275.47
137	P18858	LIG1	66	S	SPVKRPGRKAARVLGSEGEEEDEALSPAKGQ	275.32
138	P51532	SMARCA4	1627	S	GRRRPSRGSRAKPVVSDDDSEEEQEEDRSGS	275.29
139	P55081	MFAP1	132	S	DSEVEGDAWRMEREDSSEEEEEEIDDEEIER	275.24
140	Q13427	PPIG	257	S	KKEKKKRKKSKKSASSESEAENLEAQPQSTV	268.34
141	Q99613	EIF3C	11	S	*****MSRFFTTGSDSESESSLSGEELVTKP	267.30
142	Q9UQ35	SRRM2	625	S	ARRGRSRSRTPARRRSRTRSPVRRRSRSRSP	264.83
143	Q9Y2W2	WBP11	361	S	GREVEEFSEDDDEDDSDDSEAEKQSQKQHKE	264.37
144	P55081	MFAP1	133	S	SEVEGDAWRMEREDSSEEEEEEIDDEEIERR	264.31
145	P49736	MCM2	139	S	AGRGLGRMRRGLLYDSDEEDEERPARKRRQV	261.90
146	Q13895	BYSL	98	S	RERTTRLGPRMPQDGSDDEDEEWPTLEKAAT	256.47
147	Q6KC79	NIPBL	1154	S	RSSGGGRYRNRSPSDSDMEDYSPPPSLSEVA	255.79
148	Q8TEU7	RAPGEF6	230	S	VGDVDLTRLPEGPVDSEDDEEEDEEIDRTDP	255.73
149	Q96S55	WRNIP1	92	S	SSALKQPATPTAAESSEGEGEEGDDGGETES	255.67
150	Q99613	EIF3C	9	S	*******MSRFFTTGSDSESESSLSGEELVT	255.65
151	Q9BW71	HIRIP3	370	S	LGSTSGEESDLEREVSDSEAGGGPQGERKNR	255.61
152	Q9Y467	SALL2	802	S	GSESGGEKAISVRGDSEEASGAEEEVGTVAA	255.59
153	O14617	AP3D1	632	S	VPEGLDLDAWINEPLSDSESEDERPRAVFHE	255.53
154	O15541	RNF113A	253	S	EGRYGVYEDENYEVGSDDEEIPFKCFICRQS	255.40
155	O43823	AKAP8	323	S	FQLYEEPDTKLARVDSEGDFSENDDAAGDFR	255.35
156	O60341	KDM1A	131	S	AKVEYREMDESLANLSEDEYYSEEERNAKAE	241.36
157	P35659	DEK	301	S	DSSTTKKNQNSSKKESESEDSSDDEPLIKKL	236.27
158	P35659	DEK	306	S	KKNQNSSKKESESEDSSDDEPLIKKLKKPPT	236.15
159	P52756	RBM5	624	S	ENPLKRGLVAAYSGDSDNEEELVERLESEEE	235.99
160	Q13428	TCOF1	764	S	PTVTQVKAEKQEDSESSEEESDSEEAAASPA	235.92
161	Q13442	PDAP1	63	S	AGDPKKEKKSLDSDESEDEEDDYQQKRKGVE	235.84
162	Q14498	RBM39	129	S	LPHSIKLSRRRSRSKSPFRKDKSPVREPIDN	235.83
163	Q8IWX8	CHERP	813	S	SRSRSKSYSPGRRRRSRSRSPTPPSSAGLGS	235.80
164	Q8N1G4	LRRC47	518	S	MAEMKKYTLENKEEGSLSDTEADAVSGQLPD	235.78
165	Q8NHQ9	DDX55	544	S	EKKKKMNEKRKREEGSDIEDEDMEELLNDTR	235.76
166	Q8WVC0	LEO1	171	S	DEKIQNSDDEERAQGSDEDKLQNSDDDEKMQ	235.72
167	Q99613	EIF3C	178	S	PEQSADEDAEKNEEDSEGSSDEDEDEDGVSA	218.27
168	Q9H501	ESF1	663	S	KQKALAEEEASEEELPSDVDLNDPYFAEEVKQ	218.12
169	Q8NAV1	PRPF38A	194	S	PRVSALEEDMDDVESSEEEEEEDEKLERVPS	217.62

170	Q5T8D3	ACBD5	200	S	KTVNGKAESSDSGAESEEEEAQEEVKGAEQS	217.58
171	O43524	FOXO3	280	S	GRAAKKKAALQTAPESADDSPSQLSKWPGSP	217.53
172	O43823	AKAP8	328	S	EPDTKLARVDSEGDFSENDDAAGDFRSGDEE	217.32
173	O60841	EIF5B	190	S	KERSRINSSGESGDESDEFLQSRKGQKKNQK	217.19
174	Q00341	HDLBP	31	S	RSGLVPQQIKVATLNSEEESDPPTYKDAFPP	216.93
175	Q12872	SFSWAP	283	S	LAENKSDEKKKSGVSSDNEDDDDEEDGNYLH	216.78
176	Q5VZL5	ZMYM4	122	S	TDDSLEVERRVTQHESDNENEIQIQNKLKKD	216.64
177	Q7Z417	NUFIP2	112	S	LNGNAGEREISLKNLSSDEATNPISRVLNGN	216.62
178	Q7Z4V5	HDGFRP2	240	S	KKKAPSASDSDSKADSDGAKPEPVAMARSAS	216.59
179	Q86VM9	ZC3H18	83	S	DEEDRASEPKSQDQDSEVNELSRGPTSSPCE	216.49
180	Q8N7H5	PAF1	456	S	EDEARAARDKEEIFGSDADSEDDADSDDEDR	216.47
181	Q93009	USP7	18	S	HQQQQQQKAGEQQLSEPEDMEMEAGDTDDP	216.36
182	Q99543	DNAJC2	63	S	ASFQELEDKKELSEESEDEELQLEEFPMLKT	216.32
183	Q9H501	ESF1	657	S	KKRLKRKQKALAEEASEEELPSDVDLNDPYF	199.25
184	Q9UKJ3	GPATCH8	890	S	DASSDQSCYSRQRSYSDDSYSDYSDRSRRHS	198.53
185	Q9UKY1	ZHX1	47	S	PPVLTPVENTRAESISSDEEVHESVDSDNQQ	198.34
186	Q9UPT8	ZC3H4	808	S	QDRENEEGDTGNWYSSDEDEGGSSVTSILKT	197.66
187	Q9UQ35	SRRM2	416	S	RDRSPPKSPEKLPQSSSSESSPPSPQPTKVS	197.38
188	Q7Z4V5	HDGFRP2	370	S	ERADRGEAERGSGGSSGDELREDDEPVKKRG	197.35
189	P51532	SMARCA4	1631	S	PSRGSRAKPVVSDDDSEEEQEEDRSGSGSEE	197.29
190	O60841	EIF5B	183	S	EDNSKKIKERSRINSSGESGDESDEFLQSRK	197.15
191	Q99613	EIF3C	182	S	ADEDAEKNEEDSEGSSDEDEDEDGVSAATFL	196.83
192	O00267	SUPT5H	19	S	SEDSNFSEEEDSERSSDGEEAEVDEERRSAA	196.81
193	O75475	PSIP1	106	S	KFSSQQAATKQSNASSDVEVEEKETSVSKED	196.76
194	O95831	AIFM1	118	S	GLTPEQKQKKAALSASEGEEVPQDKAPSHVP	196.72
195	P52701	MSH6	275	S	GSDVEFKPDTKEEGSSDEISSGVGDSESEGL	196.67
196	Q13428	TCOF1	272	S	LPPAKRAKKPEEESESSEEGSESEEEAPAGT	196.66
197	Q4G0J3	LARP7	258	S	TSNTSISKMKRSRPTSEGSDIESTEPQKQCS	196.59
198	Q5C9Z4	NOM1	280	S	GDVEKEKKAQEAEAQSEDDDEDTEEEQGEEK	196.53
199	Q5VTR2	RNF20	136	S	LTERKALVVPEPEPDSDSNQERKDDRERGEG	196.48
200	Q8N5F7	NKAP	157	S	APEVWGLSPKNPEPDSDEHTPVEDEEPKKST	196.38
201	Q96B36	AKT1S1	202	S	SVPVWGFKEKRTEARSSDEENGPPSSPDLDR	196.27
202	Q96DR7	ARHGEF26	392	S	AVLYQNYKEKALDIDSDEESEPKEQKSDEKI	195.75
203	Q9BW71	HIRIP3	196	S	APGKASVSRKQAREESEESEAEPVQRTAKKV	194.25
204	Q9NYF8	BCLAF1	385	S	RAEGEWEDQEALDYFSDKESGKQKFNDSEGD	187.34
205	Q9P1Y6	PHRF1	915	S	SAMSKLRGAVAAEGASDTEREEPTESQGLAA	186.24
206	Q9ULX3	NOB1	201	S	EEEEEENGFEDRKDDSDDDGGGWITPSNIKQ	179.34
207	Q9UQ35	SRRM2	1102	S	QSKSQTSPKGGRSRSSSPVTELASRSPIRQD	177.36
208	Q9BQG0	MYBBP1A	738	S	SEEGEDNRSSESEEESEGEESEEEERDGDVD	177.26
209	Q7Z4V5	HDGFRP2	396	S	VKKRGRKGRGRGPPSSSDSEPEAELEREAKK	177.12
210	Q7Z4V5	HDGFRP2	236	S	GGRKKKKAPSASDSDSKADSDGAKPEPVAMA	176.99
211	Q9UQ35	SRRM2	1923	S	RRRSRSRASPVSRRRSRSRTPPVTRRRSRSR	176.97
212	Q8WVC0	LEO1	279	S	EEEQDHKSESARGSDSEDEVLRMKRKNAIAS	176.96
213	Q9UKY1	ZHX1	48	S	PVLTPVENTRAESISSDEEVHESVDSDNQQN	176.93
214	Q92733	PRCC	159	S	PVKIAAPELHKGDSDSEEDEPTKKKTILQGS	176.86
215	Q9UQ35	SRRM2	1382	S	PSLDMKEQSTRSSGHSSSELSPDAVEKAGMS	176.81
216	Q9UPT8	ZC3H4	807	S	KQDRENEEGDTGNWYSSDEDEGGSSVTSILK	176.79
217	Q8N7H5	PAF1	460	S	RAARDKEEIFGSDADSEDDADSDDEDRGQAQ	176.73
218	Q9BQG0	MYBBP1A	734	S	AEDKSEEGEDNRSSESEEESEGEESEEEERD	176.56
219	Q7Z4V5	HDGFRP2	395	S	PVKKRGRKGRGRGPPSSSDSEPEAELEREAK	176.55
220	Q8WVC0	LEO1	220	S	SEEEKANSDDERPVASDNDDEKQNSDDEEQP	176.24
221	Q13428	TCOF1	762	S	TGPTVTQVKAEKQEDSESSEEESDSEEAAAS	176.17
222	Q99613	EIF3C	181	S	SADEDAEKNEEDSEGSSDEDEDEDGVSAATF	175.68
223	Q13428	TCOF1	765	S	TVTQVKAEKQEDSESSEEESDSEEAAASPAQ	175.49
224	Q9H501	ESF1	313	S	SGPDLARGKGNIETSSEDEDDTADLFPEESG	174.91
225	Q8WVC0	LEO1	179	S	DEERAQGSDEDKLQNSDDDEKMQNTDDEERP	174.85
226	Q7Z4V5	HDGFRP2	399	S	RGRKGRGRGPPSSSDSEPEAELEREAKKSAK	172.54

227	Q8N7H5	PAF1	466	S	EEIFGSDADSEDDADSDDEDRGQAQGGSDND	171.30
228	O15541	RNF113A	85	S	TRDSGKQKAAYGDLSSEEEEENEPESLGVVY	168.63
229	O60841	EIF5B	137	S	KSKKTAKPKVEMYSGSDDDDDFNKLPKKAKG	168.34
230	Q13610	PWP1	50	S	LIAEAKEKLQEEGGGSDEEETGSPSEDGMQS	167.39
231	Q7Z4V5	HDGFRP2	232	S	RGPLGGRKKKKAPSASDSDSKADSDGAKPEP	167.35
232	Q9UQ35	SRRM2	1499	S	PEPKALPQTPRPRSRSPSSPELNNKCLTPQR	166.53
233	Q8WVC0	LEO1	277	S	DDEEEQDHKSESARGSDSEDEVLRMKRKNAI	165.39
234	Q9BQG0	MYBBP1A	732	S	KGAEDKSEEGEDNRSSESEEESEGEESEEEE	165.37
235	Q9BVG4	PBDC1	197	S	GEEENTKNGGEKGADSGEEKEEGINREDKTD	165.34
236	Q9NYF8	BCLAF1	397	S	DYFSDKESGKQKFNDSEGDDTEETEDYRQFR	164.69
237	Q9UKV3	ACIN1	838	S	KISVVSATKGVPAGNSDTEGGQPGRKRRWGA	164.54
238	Q6P6C2	ALKBH5	69	S	SGAKRKYQEDSDPERSDYEEQQLQKEEEARK	164.35
239	O14617	AP3D1	634	S	EGLDLDAWINEPLSDSESEDERPRAVFHEEE	164.30
240	Q7Z4V5	HDGFRP2	234	S	PLGGRKKKKAPSASDSDSKADSDGAKPEPVA	164.20
241	Q7Z4V5	HDGFRP2	397	S	KKRGRKGRGRGPPSSSDSEPEAELEREAKKS	163.42
242	P35659	DEK	307	S	KNQNSSKKESESEDSSDDEPLIKKLKKPPTD	162.48
243	Q13428	TCOF1	279	S	KKPEEESESSEEGSESEEEAPAGTRSQVKAS	159.42
244	Q7Z4V5	HDGFRP2	366	S	ERRRERADRGEAERGSGGSSGDELREDDEPV	159.37
245	Q8IWA0	WDR75	782	S	IPEDVDMEEEKESEDSDEENDFTEKVQDTSN	158.97
246	Q99613	EIF3C	166	S	DFESHITSYKQNPEQSADEDAEKNEEDSEGS	158.75
247	Q9Y5B6	PAXBP1	154	S	YKEDLEKSKIKTELNSSAESEQPLDKTGHVK	158.64
248	Q8WVC0	LEO1	212	S	SDDERQQLSEEEKANSDDERPVASDNDDEKQ	157.88
249	Q96B36	AKT1S1	203	S	VPVWGFKEKRTEARSSDEENGPPSSPDLDRI	157.82
250	Q9BTC0	DIDO1	809	S	PRQEAIPDLEDSPPVSDSEEQQESARAVPEK	157.73
251	Q9H307	PNN	417	S	DQEVMETNRVESVEPSENEASKELEPEMEFE	157.69
252	Q9Y2U8	LEMD3	259	S	VNGSRLVPYSCRENYSDSEEEDDDDVASSRQ	157.66
253	Q9Y6E2	BZW2	414	S	KKFVEWLQNAEEESESEGEEN**********	157.52
254	O60524	NEMF	748	S	SGRDELNEELIQEESSEDEGEYEEVRKDQDS	157.47
255	Q7Z4V5	HDGFRP2	369	S	RERADRGEAERGSGGSSGDELREDDEPVKKR	157.34
256	Q9BW71	HIRIP3	199	S	KASVSRKQAREESEESEAEPVQRTAKKVEGN	157.33
257	O14617	AP3D1	636	S	LDLDAWINEPLSDSESEDERPRAVFHEEEQR	156.47
258	Q9UQ35	SRRM2	2702	S	DSRSLSYSPVERRRPSPQPSPRDQQSSSSER	154.31
259	O15357	INPPL1	132	S	VEGEREPDPPDDRDASDGEDEKPPLPPRSGS	148.32
260	Q13428	TCOF1	270	S	GALPPAKRAKKPEEESESSEEGSESEEEAPA	147.65
261	O75683	SURF6	206	S	EPREPPGLIFNKVEVSEDEPASKAQRRKEKR	145.13
262	P52701	MSH6	279	S	EFKPDTKEEGSSDEISSGVGDSESEGLNSPV	139.79
263	Q12872	SFSWAP	604	S	DLLPLEKNRVKLDDDSDDDEESKEGQESSSS	139.66
264	O95400	CD2BP2	194	S	GARGGGKGRKGPGQPSSPQRLDRLSGLADQM	138.89
265	O95232	LUC7L3	425	S	SDTKNEVNGTSEDIKSEGDTQSN********	137.91
266	P45973	CBX5	13	S	***MGKKTKRTADSSSSEDEEEYVVEKVLDR	137.83
267	P78316	NOP14	146	S	GQSLADIEKHNDIVDSDSDAEDRGTLSAELT	137.77
268	Q03468	ERCC6	1142	S	GECSNSSGTGKTSMPSGDESIDEKLGLSYKR	137.68
269	Q5VTL8	PRPF38B	529	S	DSKDQSDKHDRRRSQSIEQESQEKQHKNKDE	137.63
270	Q8IYB3	SRRM1	551	S	RQKETSPRGRRRRSPSPPPTRRRRSPSPAPP	137.55
271	Q8NC51	SERBP1	25	S	GCVVTNRFDQLFDDESDPFEVLKAAENKKKE	137.48
272	Q8NFC6	BOD1L1	2986	S	QKSVSDPVEDKKEQESDEEEEEEEEEDEPSGA	134.20
273	P35269	GTF2F1	311	S	EGPKGVDEQSDSSEESEEEKPPEEDKEEEEE	133.85
274	Q6PD62	CTR9	970	S	RKKKKRRRHPKGEEGSDDDETENGPKPKKRR	132.61
275	Q8WVC0	LEO1	630	S	EEDKAQRLLKAKKLTSDEEGEPSGKRKAEDD	131.25
276	Q99459	CDC5L	303	S	FTKKRSKLVLPAPQISDAELQEVVKVGQASE	129.34
277	Q9UQ35	SRRM2	1501	S	PKALPQTPRPRSRSPSSPELNNKCLTPQRER	128.34
278	Q9Y467	SALL2	806	S	GGEKAISVRGDSEEASGAEEEVGTVAAAATA	128.26
279	P11717	IGF2R	2409	S	KSVKALSSLHGDDQDSEDEVLTIPEVKVHSG	127.99
280	P21127	CDK11B	277	S	PAQLKEEKMEERDLLSDLQDISDSERKTSSA	127.96
281	P35611	ADD1	358	S	LLNPEKYKAKSRSPGSPVGEGTGSPPKWQIG	126.93
282	P51532	SMARCA4	1570	S	SVFTSVRQKIEKEDDSEGEESEEEEEGEEEG	126.83
283	Q9BW71	HIRIP3	291	S	CKQKSQAKRLLGDSDSEEEQKEAASSGDDSG	126.14

284	Q9BXJ9	NAA15	855	S	PPGYEEDMKITVNGDSSAEAEELANEI****	119.67
285	Q9H6S0	YTHDC2	1090	S	QEPSSFRVDGIPNDSSDSEMEDKTTANLAAL	119.47
286	Q9H1E3	NUCKS1	132	S	QEEEDEAPFQEKDSGSDEDFLMEDDDDSDYG	118.59
287	Q9H501	ESF1	312	S	DSGPDLARGKGNIETSSEDEDDTADLFPEES	118.51
288	Q9NXG2	THUMPD1	88	S	YGPEKFTDKDQQPSGSEGEDDDAEAALKKEV	118.41
289	Q9UQE7	SMC3	1067	S	KATLVMKKGDVEGSQSQDEGEGSGESERGSG	118.27
290	P78316	NOP14	148	S	SLADIEKHNDIVDSDSDAEDRGTLSAELTAA	118.15
291	P17812	CTPS1	574	S	CRLSPRDTYSDRSGSSSPDSEITELKFPSIN	117.98
292	P35251	RFC1	69	S	FKQKQPSKKKRIIYDSDSESEETLQVKNAKK	117.95
293	P51116	FXR2	410	S	PGSGRGSGGSDKAGYSTDESSSSSLHATRTY	117.88
294	Q03701	CEBPZ	629	S	KAKPGLRSQLDDHPESDDEENFIDANDDEDM	117.77
295	Q08AD1	CAMSAP2	1148	S	PPEKADVPVEKYDGESDKEQFDDDQKVCCGF	116.92
296	Q13428	TCOF1	273	S	PPAKRAKKPEEESESSEEGSESEEEAPAGTR	116.87
297	Q13428	TCOF1	347	S	AGKPEEDSESSSEESSDSEEETPAAKALLQA	116.63
298	Q3YEC7	RABL6	471	S	PLPAGPVPSQDITLSSEEEAEVAAPTKGPAP	116.59
299	Q4G0J3	LARP7	337	S	KEASEASKENRDIEISTEEEKDTGDLKDSSL	116.38
300	Q8N1G2	CMTR1	55	S	SHGAKASTTSLSGSDSETEGKQHSSDSFDDA	116.17
301	Q96G74	OTUD5	177	S	GGGSPEREEVGAGYNSEDEYEAAAARIEAMD	115.99
302	Q96ST2	IWS1	157	S	HASDSENEDVGKHPASDSEIEELQKSPASDS	115.93
303	Q9BW71	HIRIP3	330	S	RKSEDRTQLKGGKRLSGSSEDEEDSGKGEPT	115.88
304	Q9NW75	GPATCH2	115	S	SKDYRENHNNNKKDHSDSDDQMLVAKRRPSS	115.83
305	Q9NZ63	C9orf78	15	S	*MPVVRKIFRRRRGDSESEEDEQDSEEVRLK	115.79
306	Q9UQ35	SRRM2	510	S	SRSRTPTKRGHSRSRSPQWRRSRSAQRWGRS	115.71
307	Q9Y2U8	LEMD3	140	S	ASAAPAAGSKVLLGFSSDESDVEASPRDQAG	115.62
308	P51532	SMARCA4	1586	S	EGEESEEEEEGEEEGSESESRSVKVKIKLGR	109.22
309	Q13442	PDAP1	57	S	EGGDGAAGDPKKEKKSLDSDESEDEEDDYQQ	106.27
310	P51532	SMARCA4	1575	S	VRQKIEKEDDSEGEESEEEEEGEEEGSESES	106.14
311	O75475	PSIP1	273	S	ESKRKNLAKTGVTSTSDSEEEGDDQEGEKKR	105.88
312	Q9BW71	HIRIP3	332	S	SEDRTQLKGGKRLSGSSEDEEDSGKGEPTAK	105.73
313	Q13427	PPIG	744	S	ENDHVHEKNKKFDHESSPGTDEDKSG*****	105.32
314	O43847	NRD1	86	S	DLGENSRVARLGADESEEEGRRGSLSNAGDP	104.58
315	P18858	LIG1	201	S	KPLKTSKAETPTESVSEPEVATKQELQEEEE	104.54
316	Q03468	ERCC6	429	S	QKKVPVQEIDDDFFPSSGEEAEAASVGEGGG	104.34
317	P35269	GTF2F1	305	S	KAPQQEEGPKGVDEQSDSSEESEEEKPPEED	103.38
318	P51116	FXR2	533	S	PYSLLDTSEPEPPVDSEPGEPPPASARRRRS	103.36
319	Q8IY81	FTSJ3	458	S	ADTFLSDLPRDDIYVSDVEDDGDDTSLDSDL	102.85
320	Q8WVC0	LEO1	608	S	RYKGGIREERARIYSSDSDEGSEEDKAQRLL	101.68
321	P35659	DEK	303	S	STTKKNQNSSKKESESEDSSDDEPLIKKLKK	101.37
322	Q6P158	DHX57	127	S	KALLRDLQEQDADAGSERGLSGEEEDDEPDC	101.19
323	Q03468	ERCC6	430	S	KKVPVQEIDDDFFPSSGEEAEAASVGEGGGG	99.18
324	P52701	MSH6	285	S	KEEGSSDEISSGVGDSESEGLNSPVKVARKR	98.95
325	Q9Y2U8	LEMD3	261	S	GSRLVPYSCRENYSDSEEEDDDDVASSRQVL	98.83
326	P16383	GCFC2	19	S	RPKRTFRQRAADSSDSDGAEESPAEPGAPRE	98.63
327	P05388	RPLP0	304	S	PAAAAAPAKVEAKEESEESDEDMGFGLFD**	98.56
328	P27824	CANX	564	S	EEKQKSDAEEDGGTVSQEEEDRKPKAEEDEI	98.39
329	P55884	EIF3B	125	S	EQARDERSDSRAQAVSEDAGGNEGRAAEAEP	98.35
330	Q08945	SSRP1	444	S	GLKEGMNPSYDEYADSDEDQHDAYLERMKEE	98.33
331	Q13435	SF3B2	309	S	EEMETDARSSLGQSASETEEDTVSVSKKEKN	98.31
332	Q14839	CHD4	103	S	EGPEFVEEEEEVALRSDSEGSDYTPGKKKKK	98.25
333	Q7Z4V5	HDGFRP2	664	S	RPGSDRQERERARGDSEALDEES********	98.14
334	Q86VM9	ZC3H18	34	S	QPQGLSDDDILRDSGSDQDLDGAGVRASDLE	97.57
335	Q86X53	ERICH1	254	S	EEDLTRARQEEGADASEEDPTPAGEEDVKDA	94.60
336	Q8NAF0	ZNF579	194	S	PSTLAAPTSAAEPRESESEEAEAGAAELRAE	94.35
337	Q8NE71	ABCF1	105	S	DDGEEKELMERLKKLSVPTSDEEDEVPAPKP	94.34
338	Q8WVC0	LEO1	229	S	DERPVASDNDDEKQNSDDEEQPQLSDEEKMQ	93.54
339	Q92538	GBF1	1298	S	LQATARADAPDAGAQSDSELPSYHQNDVSLD	86.34
340	Q92733	PRCC	157	S	KEPVKIAAPELHKGDSDSEEDEPTKKKTILQ	84.39

341	Q92917	GPKOW	35	S	SFGFTRTSARRRLADSGDGAGPSPEEKDFLK	83.54
342	Q99613	EIF3C	18	S	RFFTTGSDSESESSLSGEELVTKPVGGNYGK	82.39
343	Q9UQ35	SRRM2	1652	S	RSGSSSKGRGPSPEGSSSTESSPEHPPKSRT	81.77
344	Q96ST2	IWS1	159	S	SDSENEDVGKHPASDSEIEELQKSPASDSET	81.74
345	Q9BW71	HIRIP3	333	S	EDRTQLKGGKRLSGSSEDEEDSGKGEPTAKG	81.71
346	Q9UQ35	SRRM2	1653	S	SGSSSKGRGPSPEGSSSTESSPEHPPKSRTA	81.68
347	P35251	RFC1	71	S	QKQPSKKKRIIYDSDSESEETLQVKNAKKPP	80.66
348	Q9NW75	GPATCH2	117	S	DYRENHNNNKKDHSDSDDQMLVAKRRPSSNI	80.64
349	Q9UQ35	SRRM2	418	S	RSPPKSPEKLPQSSSSESSPPSPQPTKVSRH	80.62
350	Q08945	SSRP1	667	S	SSRQLSESFKSKEFVSSDESSSGENKSKKKR	80.59
351	Q9UQ35	SRRM2	1762	S	SASSPRTKTTSRRGRSPSPKPRGLQRSRSRS	80.57
352	Q9UQ35	SRRM2	1542	S	TVARTPLGQRSRSGSSQELDVKPSASPQERS	80.49
353	Q9BW71	HIRIP3	555	S	PDWSHMRGIISSDGESN**************	79.42
354	Q9GZR7	DDX24	82	S	SLFSKEAPKRKAQAVSEEEEEEEGKSSSPKK	79.37
355	O94979	SEC31A	527	S	DGANVALKDSDQVAQSDGEESPAAEEQLLGE	79.37
356	Q9H0D6	XRN2	501	S	PSPLGGIKRKAEDSDSEPEPEDNVRLWEAGW	79.29
357	P19338	NCL	34	S	PPPKEVEEDSEDEEMSEDEEDDSSGEEVVIP	79.29
358	Q9HAW4	CLSPN	65	S	IFVSKKLKNRKVLQDSDSETEDTNASPEKTT	79.25
359	Q9NQ55	PPAN	359	S	RKKSLEGMKKARVGGSDEEASGIPSRTASLE	79.15
360	Q9P289	MST4	304	S	DRFKRWKAEGHSDDESDSEGSDSESTSRENN	78.93
361	Q9UQ35	SRRM2	415	S	TRDRSPPKSPEKLPQSSSSESSPPSPQPTKV	78.91
362	Q9Y5T5	USP16	415	S	NDKNLKKTVEDEDQDSEEEKDNDSYIKERSD	78.90
363	Q9Y6R4	MAP3K4	66	S	RQEGTLGDSACKSPESDLEDFSDETNTENLY	78.89
364	Q9UQ35	SRRM2	1764	S	SSPRTKTTSRRGRSPSPKPRGLQRSRSRSRR	78.85
365	Q9Y2U8	LEMD3	144	S	PAAGSKVLLGFSSDESDVEASPRDQAGGGGR	78.84
366	Q9UQ35	SRRM2	1379	S	ETDPSLDMKEQSTRSSGHSSSELSPDAVEKA	78.82
367	Q8WVC0	LEO1	238	S	DDEKQNSDDEEQPQLSDEEKMQNSDDERPQA	78.73
368	Q9UQ35	SRRM2	1657	S	SKGRGPSPEGSSSTESSPEHPPKSRTARRGS	78.69
369	Q8NE71	ABCF1	109	S	EKELMERLKKLSVPTSDEEDEVPAPKPRGGK	78.67
370	Q9HAW4	CLSPN	67	S	VSKKLKNRKVLQDSDSETEDTNASPEKTTYD	78.65
371	Q9UQ35	SRRM2	1378	S	LETDPSLDMKEQSTRSSGHSSSELSPDAVEK	78.63
372	Q9Y2U8	LEMD3	141	S	SAAPAAGSKVLLGFSSDESDVEASPRDQAGG	78.61
373	O60841	EIF5B	595	S	DKKPSKEMSSDSEYDSDDDRTKEERAYDKAK	78.60
374	Q3YEC7	RABL6	470	S	PPLPAGPVPSQDITLSSEEEAEVAAPTKGPA	78.58
375	Q8WVC0	LEO1	273	S	HRHSDDEEEQDHKSESARGSDSEDEVLRMKR	78.53
376	Q1KMD3	HNRNPUL2	161	S	EQGLGKREEDEPEERSGDETPGSEVPGDKAA	78.44
377	O75400	PRPF40A	938	S	TGKDSGNWDTSGSELSEGELEKRRRTLLEQL	78.38
378	P16383	GCFC2	17	S	AHRPKRTFRQRAADSSDSDGAEESPAEPGAP	78.35
379	P17812	CTPS1	575	S	RLSPRDTYSDRSGSSSPDSEITELKFPSINH	78.33
380	P25205	MCM3	668	S	YFKKVLEKEKKRKKRSEDESETEDEEEKSQE	78.25
381	P52701	MSH6	254	S	RRSSRQIKKRRVISDSESDIGGSDVEFKPDT	78.18
382	Q07666	KHDRBS1	20	S	DDPAARMSRSSGRSGSMDPSGAHPSVRQTPS	78.14
383	Q5C9Z4	NOM1	321	S	KRVRFAEDEEKSENSSEDGDITDKSLCGSGE	77.72
384	Q6WKZ4	RAB11FIP1	202	S	DTASAIIPSTTPSVDSDDESVVKDKKKKSKI	77.66
385	Q86VM9	ZC3H18	46	S	DSGSDQDLDGAGVRASDLEDEESAARGPSQE	77.65
386	Q8N1G2	CMTR1	53	S	SVSHGAKASTTSLSGSDSETEGKQHSSDSFD	77.50
387	Q8NAF0	ZNF579	196	S	TLAAPTSAAEPRESESEEAEAGAAELRAELA	76.59
388	Q8NFC6	BOD1L1	266	S	RTSEDMADKEKSTADSGGEGLETAPKSEEFS	76.35
389	Q8WVC0	LEO1	607	S	NRYKGGIREERARIYSSDSDEGSEEDKAQRI	76.34
390	Q96E09	FAM122A	37	S	EGGGSGGGGGLRRSNSAPLIHGLSDTSPVFQ	74.36
391	Q96NB3	ZNF830	351	S	LTIKELQKKEEENADSDDEGELQDLLSQDWR	73.59
392	Q99613	EIF3C	13	S	***MSRFFTTGSDSESESSLSGEELVTKPVG	72.31
393	Q9BRS2	RIOK1	21	S	LLMSRVVPGQFDDADSSDSENRDLKTVKEKD	72.11
394	Q9BTC0	DIDO1	811	S	QEAIPDLEDSPPVSDSEEQQESARAVPEKST	67.34
395	Q9BXJ9	NAA15	856	S	PGYEEDMKITVNGDSSAEAEELANEI*****	65.47
396	Q9H6Z4	RANBP3	355	S	VSSDANRENAAAESGSESSSQEATPEKESLA	65.38
397	Q9NXG2	THUMPD1	86	S	DMYGPEKFTDKDQQPSGSEGEDDDAEAALKK	64.55

398	Q9UKV3	ACIN1	898	S	GQEAVVDLHADDSRISEDETERNGDDGTHDK	64.49
399	O60841	EIF5B	589	S	DSGKTLDKKPSKEMSSDSEYDSDDDRTKEER	64.38
400	Q5VVJ2	MYSM1	218	S	GRADPNLNAVKIEKLSDDEEVDITDEVDELS	64.35
401	P19338	NCL	41	S	EDSEDEEMSEDEEDDSSGEEVVIPQKKGKKA	64.30
402	Q9UQ35	SRRM2	1497	S	SSPEPKALPQTPRPRSRSPSSPELNNKCLTP	64.25
403	Q8IWX8	CHERP	815	S	SRSKSYSPGRRRRSRSRSPTPPSSAGLGSNS	64.18
404	Q9BW71	HIRIP3	372	S	STSGEESDLEREVSDSEAGGGPQGERKNRSS	63.41
405	Q9UQ35	SRRM2	1539	S	DQKTVARTPLGQRSRSGSSQELDVKPSASPQ	63.34
406	Q8WVC0	LEO1	610	S	KGGIREERARIYSSDSDEGSEEDKAQRLLKA	62.38
407	Q13428	TCOF1	771	S	AEKQEDSESSEEESDSEEAAASPAQVKTSVK	62.31
408	Q9UQ35	SRRM2	2046	S	RSRTPLLPRKRSRSRSPLAIRRRSRSRTPRT	61.97
409	Q9UQ35	SRRM2	1654	S	GSSSKGRGPSPEGSSSTESSPEHPPKSRTAR	61.88
410	Q14839	CHD4	105	S	PEFVEEEEEVALRSDSEGSDYTPGKKKKKKL	61.82
411	Q9UQ35	SRRM2	1552	S	SRSGSSQELDVKPSASPQERSESDSSPDSKA	61.34
412	Q8WVX3-2	C4orf3	7	S	*********MTSLINSPINRRPLQNVEGNNR	60.81
413	O14974	PPP1R12A	509	S	LAYVAPTIPRRLASTSDIEEKENRDSSSLRT	60.67
414	P16383	GCFC2	16	S	MAHRPKRTFRQRAADSSDSDGAEESPAEPGA	60.54
415	P18858	LIG1	141	S	KQLPKRTIQEVLEEQSEDEDREAKRKKEEEE	60.37
416	P45973	CBX5	14	S	**MGKKTKRTADSSSSEDEEEYVVEKVLDRR	60.28
417	O75822	EIF3J	127	S	EQLADKLRLKKLQEESDLELAKETFGVNNAV	59.57
418	P27815-7	PDE4A	18	S	RSRSALSVAGTGDERSRETPESDRANMLGAD	59.36
419	P48634	PRRC2A	342	S	AGAHEEVDYTEKLKFSDEEDGRDSDEEGAEG	59.18
420	P51116	FXR2	447	S	RGRGRRTGGPAYGPSSDVSTASETESEKREE	59.15
421	Q13185	CBX3	95	S	QKAGKEKDGTKRKSLSDSESDDSKSKKKRDA	59.14
422	Q13427	PPIG	254	S	RKHKKEKKKRKKSKKSASSESESEAENLEAQPQ	59.09
423	Q13428	TCOF1	85	S	ALQAKKTRVSDPISTSESSEEEEEAEAETAK	59.05
424	Q13435	SF3B2	307	S	EEEEMETDARSSLGQSASETEEDTVSVSKKE	59.04
425	Q14137	BOP1	127	S	EMASARIGDEYAEDSSDEEDIRNTVGNVPLE	58.97
426	Q15054	POLD3	307	S	KVLQKEKKRGKRVALSDDETKETENMRKKRR	58.96
427	Q3YEC7	RABL6	464	S	DQPRGSPPLPAGPVPSQDITLSSEEEAEVAA	58.94
428	Q5JRA6	MIA3	288	S	LDLKTKFGSTADALVSDDETTRLVTSLEDDF	58.93
429	Q5T200	ZC3H13	1465	S	AHSLGSGAGEGYEPISDDELDEILAGDAEKR	58.88
430	Q6IN85	SMEK1	117	S	GKDPSVDITQDLVDESEEERFDDMSSPGLEL	58.67
431	Q6NSI8-2	KIAA1841	697	S	PRDGTVSKSNRKSGLSSVLLMTVLLC*****	58.64
432	Q6WKZ4	RAB11FIP1	206	S	AIIPSTTPSVDSDDESVVKDKKKKSKIKTLL	58.61
433	Q86V59	PNMAL1	335	S	QDARAEAESPGGASESDQDGGHESPPKKKAV	58.59
434	Q8IY81	FTSJ3	471	S	YVSDVEDDGDDTSLDSDLDPEELAGVRGHQG	58.46
435	Q8TEA8	DTD1	197	S	SKERNTPRKEDRSASSGAEGDVSSEREP***	58.34
436	Q8WVC0	LEO1	271	S	EEHRHSDDEEEQDHKSESARGSDSEDEVLRM	57.94
437	Q92538	GBF1	1300	S	ATARADAPDAGAQSDSELPSYHQNDVSLDRG	57.83
438	Q96MU7	YTHDC1	308	S	GSDEKKKERKRARGISPIVFDRSGSSASESY	57.75
439	Q96ST2	IWS1	183	S	PASDSETEDALKPQISDSESEEPPRHQASDS	57.68
440	Q99613	EIF3C	16	S	MSRFFTTGSDSESESSLSGEELVTKPVGGNY	57.64
441	Q9BQ52	ELAC2	212	S	WQSPERPLSRLSPERSSDSESNENEPHLPHG	57.63
442	Q9H7L9	SUDS3	45	S	LESAEDDERSCRGRESDEDTEDASETDLAKH	57.39
443	Q9NR30	DDX21	13	S	***MPGKLRSDAGLESDTAMKKGETLRKQTE	57.34
444	Q9NXX6	NSMCE4A	30	S	RGRDPHRDRTRSRSRSRSPLSPRSRRGSARE	56.91
445	Q9NYF8	BCLAF1	389	S	EWEDQEALDYFSDKESGKQKFNDSEGDDTEE	56.65
446	Q9UBB9	TFIP11	210	S	SERTTQSMQDFPVVDSEEEAEEEFQKELSQW	56.41
447	Q9UH62	ARMCX3	61	S	VDDAGDCSGARYNDWSDDDDDSNESKSIVWY	56.34
448	Q9UKD2	MRTO4	235	S	DDLPESASESTEESDSEDDD***********	54.12
449	Q9ULX3	NOB1	184	S	HELQELLIDRGEDVPSEEEEEEENGFEDRKD	52.38
450	Q9UQ35	SRRM2	1103	S	SKSQTSPKGGRSRSSSPVTELASRSPIRQDR	51.42
451	Q9Y5B6	PAXBP1	155	S	KEDLEKSKIKTELNSSAESEQPLDKTGHVKD	51.29
452	Q9UQ35	SRRM2	1550	S	QRSRSGSSQELDVKPSASPQERSESDSSPDS	49.56
453	Q9UQ35	SRRM2	2044	S	RSRSRTPLLPRKRSRSRSPLAIRRRSRSRTP	46.29
454	Q5JSH3	WDR44	403	S	IMRRTKEYVSNDAAQSDDEEKLQSQPTDTDG	46.25

455	Q6NSI8-2	KIAA1841	698	S	RDGTVSKSNRKSGLSSVLLMTVLLC******	42.77
456	Q9NXX6	NSMCE4A	32	S	RDPHRDRTRSRSRSRSPLSPRSRRGSARERR	40.99
457	Q9UQ35	SRRM2	2042	S	RRRSRSRTPLLPRKRSRSRSPLAIRRRSRSR	40.94
458	O94806	PRKD3	30	S	LPTAIPAVLPAASPCSSPKTGLSARLSNGSP	40.93
459	Q5T0N5	FNBP1L	295	S	DFPFEDYSQHIYRTISDGTISASKQESGKMD	40.91
460	P12694	BCKDHA	347	S	RIGHHSTSDDSSAYRSVDEVNYWDKQDHPIS	40.88
461	O15027	SEC16A	1172	S	HRDPYGEEVDRRSVHSEHSARSLHSAHSLAS	40.84
462	P05386	RPLP1	101	S	AAPAEEKKVEAKKEESEESDDDMGFGLFD**	39.85
463	Q9UQ35	SRRM2	2032	S	RSRSRTPPAIRRRSRSRTPLLPRKRSRSRSP	39.83
464	O15027	SEC16A	1169	S	PDPHRDPYGEEVDRRSVHSEHSARSLHSAHS	39.76
465	O43719	HTATSF1	702	S	KEVEDADEKLFEDDDSNEKLFDEEEDSSEKL	39.74
466	P18583	SON	1780	S	RSAASPVVSSMPERASESSSEEKDDYEIFVK	39.72
467	P51532	SMARCA4	655	S	EAWLEMNPGYEVAPRSDSEESGSEEEEEEEE	39.71
468	Q15019	SEPT2	218	S	IEEHNIKIYHLPDAESDEDEDFKEQTRLLKA	39.69
469	Q14498	RBM39	136	S	SRRRSRSKSPFRKDKSPVREPIDNLTPEERD	39.67
470	Q7Z4V5	HDGFRP2	264	S	AMARSASSSSSSSSSDSDVSVKKPPRGRKP	39.65
471	P08238	HSP90AB1	226	S	YPITLYLEKEREKEISDDEAEEEKGEKEEED	39.51
472	Q9Y4P1	ATG4B	383	S	SLDSSDVERLERFFDSEDEDFEILSL*****	39.48
473	O60284	ST18	690	S	THGKTEEEKEKDPVSSLENLEEKKFPGEASI	39.44
474	P07900	HSP90AA1	263	S	EEKESEDKPEIEDVGSDEEEEKKDGDKKKKK	39.40
475	Q9H1E3	NUCKS1	144	S	DSGSDEDFLMEDDDDSDYGSSKKKNKKMVKK	39.39
476	Q9UQ35	SRRM2	2020	S	RSRSRTSPVTRRRSRSRTPPAIRRRSRSRTP	39.35
477	O43719	HTATSF1	713	S	EDDDSNEKLFDEEEDSSEKLFDDSDERGTLG	39.34
478	Q9Y2W1	THRAP3	53	S	SRSRKRRLSSRSRSRSYSPAHNRERNHPRVY	39.33
479	P07900	HSP90AA1	231	S	YPITLFVEKERDKEVSDDEAEEKEDKEEEKE	39.27
480	Q08945	SSRP1	668	S	SRQLSESFKSKEFVSSDESSSGENKSKKKRR	39.22
481	P35269	GTF2F1	217	S	ASELRIHDLEDDLEMSSDASDASGEEGGRVP	39.18
482	Q9UJX6	ANAPC2	218	S	LDSRYARRRYYRLLQSPLCAGCSSDKQQCWC	39.17
483	Q7Z5K2	WAPAL	77	S	PKVEEESTGDPFGFDSDDESLPVSSKNLAQV	39.15
484	Q8N5F7	NKAP	27	S	REASGSGGRRRSSSKSPKPSKSARSPRGRRS	39.13
485	Q16629	SRSF7	202	S	SRYFQSPSRSRSRSRSISRPRSSRSKSRSPS	38.64
486	O75400	PRPF40A	933	S	SPKKKTGKDSGNWDTSGSELSEGELEKRRRT	38.63
487	O75494	SRSF10	133	S	RSRSRSYERRRSRSRSFDYNYRRSYSPRNSR	38.58
488	O60284	ST18	689	S	KTHGKTEEEKEKDPVSSLENLEEKKFPGEAS	38.57
489	O95218	ZRANB2	120	S	GFNERENVEYIEREESDGEYDEFGRKKKKYR	38.53
490	P51532	SMARCA4	657	S	WLEMNPGYEVAPRSDSEESGSEEEEEEEEEE	38.52
491	P24534	EEF1B2	140	S	QYESKKAKKPALVAKSSILLDVKPWDDETDM	37.56
492	Q5H9R7	PPP6R3	617	S	EACCKERIQQFDDGGSDEEDIWEEKHIAFTP	37.38
493	Q5F1R6	DNAJC21	423	S	EGVKVDPEDTNLNQDSAKELEDSPQENVSVT	37.37
494	Q9H7J1	PPP1R3E	81	S	SAPAGGGGARAPRSRSPDTRKRVRFADALGL	37.36
495	Q8IYB3	SRRM1	389	S	PPKRTSSPPRKTRRLSPSASPPRRRHRPSPP	37.33
496	P51531	SMARCA2	1572	S	RPNRGKAKPVVSDFDSDEEQDEREQSEGSGT	36.38
497	Q9BYW2	SETD2	1988	S	PINEETPSQDEEEGVSDVESERSQEQPDKTV	36.35
498	Q9Y2W1	THRAP3	51	S	SLSRSRKRRLSSRSRSRSYSPAHNRERNHPR	36.34
499	Q9UN86	G3BP2	141	S	VHNDMFRYEDEVFGDSEPELDEESEDEVEEE	36.33
500	Q8NDI1	EHBP1	171	S	CIFLREGKATDEDMQSLASLMSMKQADIGNL	35.69
501	O96007	MOCS2	3	S	*************MSSLEISSSCFSLETKLP	35.33
502	P19338	NCL	28	S	DPKKMAPPPKEVEEDSEDEEMSEDEEDDSSG	34.52
503	O75475	PSIP1	275	S	KRKNLAKTGVTSTSDSEEEGDDQEGEKKRKG	31.30
504	P49321	NASP	503	S	DKTEEMPNDSVLENKSLQENEEEEIGNLELA	31.29
505	Q8TDD1	DDX54	75	S	GPGRPLPTFPTSECTSDVEPDTREMVRAQNK	31.28
506	O75400	PRPF40A	935	S	KKKTGKDSGNWDTSGSELSEGELEKRRRTLI	30.33
507	P08238	HSP90AB1	255	S	EDKDDEEKPKIEDVGSDEEDDSGKDKKKKTK	30.32
508	P05386	RPLP1	104	S	AEEKKVEAKKEESEESDDDMGFGLFD*****	30.31
509	O96007	MOCS2	2	S	**************MSSLEISSSCFSLETKL	30.26
510	Q9NW75	GPATCH2	284	S	GLFTNDEGRQGDDEQSDWFYEKESGGACGIT	30.25
511	O94913	PCF11	509	S	SPKRRDRRSPKRRQRSMSPTSTPKAGKIRQS	30.24

512	O15027	SEC16A	1175	S	PYGEEVDRRSVHSEHSARSLHSAHSLASRRS	29.54
513	P35269	GTF2F1	218	S	SELRIHDLEDDLEMSSDASDASGEEGGRVPK	28.51
514	P67809	YBX1	176	S	NYQNSESGEKNEGSESAPEGQAQQRRPYRRR	27.34
515	Q96HR8	NAF1	315	S	SWKNDQEPPPEALDFSDDEKEKEAKQRKKSQ	26.48
516	Q13428	TCOF1	935	S	GPSAAQAGKQDDSGSSSEESDSDGEAPAAVT	26.47
517	Q96B97	SH3KBP1	230	S	FGDIFKDKPIKLRPRSIEVENDFLPVEKTIG	18.00
518	P24534	EEF1B2	141	S	YESKKAKKPALVAKSSILLDVKPWDDETDMA	13.88
519	Q96IZ7	RSRC1	121	S	RRSRSRPRLRSHSRSSERSSHRRTRSRSRDR	13.56
520	Q92934	BAD	118	S	NLWAAQRYGRELRRMSDEFVDSFKKGLPRPK	13.49
521	Q9NRW4	DUSP22	58	S	PMLEGVKYLCIPAADSPSQNLTRHFKESIKF	12.40
522	Q8N108	MIER1	160	S	QEIIRPRRCKYFDTNSEVEEESEEDEDYIPS	11.25
523	Q9H6Z4	RANBP3	96	S	PEAQLPPFPRELAGRSAGGSSPEGGEDSDRE	10.18
524	Q04656	ATP7A	1473	S	NYSRASINSLLSDKRSLNSVVTSEPDKHSLL	9.63
525	Q12872	SFSWAP	866	S	KKKRRSRSRTKSKARSQSVSPSKQAAPRPAA	8.94
526	Q5SW79	CEP170	1112	S	PTRTSLLRRARLGEASDSELADADKASVASE	8.69
527	Q9BRS2	RIOK1	22	S	LMSRVVPGQFDDADSSDSENRDLKTVKEKDD	8.65
528	Q8NC51	SERBP1	394	S	GGRPNRGSRTDKSSASAPDVDDPEAFPALA*	8.63
529	P49792	RANBP2	2900	S	VGTQSAGKVGEDEDGSDEEVVHNEDIHFEPI	8.11
530	P98175	RBM10	60	S	PREYGSQEGKHDYDDSSEEQSAEDSYEASPG	7.71
531	Q5THJ4	VPS13D	1042	S	SFDIPTGSLRDSRAQSPVSGPNVAHLTDGAT	7.49
532	Q96IZ7	RSRC1	120	S	TRRSRSRPRLRSHSRSSERSSHRRTRSRSRD	7.47
533	Q9UKJ3	GPATCH8	352	S	KSSDQGLQKVGDSDGSSNLDGKKEDEDPQDG	7.45
534	P67809	YBX1	174	S	QQNYQNSESGEKNEGSESAPEGQAQQRRPYR	7.42
535	Q9H7J1	PPP1R3E	79	S	ARSAPAGGGGARAPRSRSPDTRKRVRFADAL	7.38
536	Q9UQ35	SRRM2	250	S	KSKRKSKDKKRKRSRSTTPAPKSRRAHRSTS	7.37
537	Q9UQ35	SRRM2	957	S	TSRTTPRRSRSVSPCSNVESRLLPRYSHSGS	7.23
538	Q9Y6X9	MORC2	743	S	KLSPATPSRKRSVAVSDEEEVEEEAERRKER	7.19
539	Q92576	PHF3	1133	S	CKICIGRMAPPVDDLSPKKVKVVVGVARKHS	7.01
540	P06748	NPM1	112	S	PVVLRLKCGSGPVHISGQHLVAVEEDAESED	6.97
541	P51858	HDGF	132	S	EAAEGDGDKKGNAEGSSDEEGKLVIDEPAKE	6.90
542	Q8IXT5	RBM12B	280	S	RKRSHSKSPRRTRSRSPLGFYVHLKNLSLSI	6.74
543	P23588	EIF4B	409	S	PKLERRPRERHPSWRSEETQERERSRTGSES	6.67
544	O15541	RNF113A	47	S	KRPACDPEPGESGSSSDEGCTVVRPEKKRVT	6.54
545	O95382	MAP3K6	916	S	LGDPFLQPGKRSRSPSSPRHAPRPSDAPSAS	6.51
546	Q12789	GTF3C1	1068	S	VRCPRVRKNSSTDQGSDEEGSLQKEQESAMD	6.48
547	Q8IYB3	SRRM1	530	S	GEVGRRRRHSPSRSASPSPRKRQKETSPRGR	6.40
548	Q8N5F7	NKAP	24	S	SPDREASGSGGRRRSSSKSPKPSKSARSPRG	6.39
549	Q9H1E3	NUCKS1	130	S	EEQEEEDEAPFQEKDSGSDEDFLMEDDDDSD	6.36
550	Q96ST2	IWS1	438	S	DAVSDKSGKREKTIASDSEEEAGKELSDKKN	6.34
551	Q13185	CBX3	176	S	PQIVIAFYEERLTWHSCPEDEAQ********	6.32
552	Q8N9T8	KRI1	93	S	PRIYQKDATFYNRTASSSDSEEDPEALEKQK	6.31
553	Q8N9T8	KRI1	94	S	RIYQKDATFYNRTASSSDSEEDPEALEKQKK	6.24
554	Q9UQ35	SRRM2	741	S	SSSERKNKSRTSQRRSRSNSSPEMKKSRISS	6.23
555	Q96ST2	IWS1	400	S	EEEKVAKRKAAVLSDSEDEEKASAKKSRVVS	6.16
556	Q9C073	FAM117A	213	S	PSGSPVLRLSPCLHRSLEGLNQELEEVFVKE	6.14
557	P45973	CBX5	12	S	****MGKKTKRTADSSSSEDEEEYVVEKVLD	6.12
558	P19338	NCL	42	S	DSEDEEMSEDEEDDSSGEEVVIPQKKGKKAA	6.10
559	P45973	CBX5	11	S	*****MGKKTKRTADSSSSEDEEEYVVEKVL	6.03
560	Q96ST2	IWS1	415	S	SEDEEKASAKKSRVVSDADDSDSDAVSDKSG	5.79
561	P26373	RPL13	139	S	LILFPRKPSAPKKGDSSAEELKLATQLTGPV	5.72
562	Q12872	SFSWAP	868	S	KRRSRSRTKSKARSQSVSPSKQAAPRPAAPA	5.57
563	Q5BKY9	FAM133B	196	S	RKMYSEDKPLSSESLSESEYIEEVRAKKKKS	5.54
564	Q5BKY9	FAM133B	194	S	KKRKMYSEDKPLSSESLSESEYIEEVRAKKK	5.44
565	Q6PD62	CTR9	1037	S	DEDKLKIADEGHPRNSNSNSDSDEDEQRKKC	5.43
566	Q8N108	MIER1	166	S	RRCKYFDTNSEVEEESEEDEDYIPSEDWKKE	5.34
567	Q92769	HDAC2	422	S	SIRASDKRIACDEEFSDSEDEGEGGRRNVAD	5.02
568	P98175	RBM10	736	S	RPSPPRGLVAAYSGESDSEEEQERGGPEREE	4.92

569	Q5SNT6	FAM21B	451	S	NLKPSSETKTQKGLFSDEEDSEDLFSSQSAS	4.88
570	Q8IYB3	SRRM1	463	S	RESPSPAPKPRKVELSESEEDKGGKMAAADS	4.74
571	Q13428	TCOF1	349	S	KPEEDSESSSEESSDSEEETPAAKALLQAKA	4.69
572	P46937	YAP1	61	S	QAPPAGHQIVHVRGDSETDLEALFNAVMNPK	4.68
573	Q06265	EXOSC9	306	S	QRITAFKMEKAPIDTSDVEEKAEEIIAEAEP	4.67
574	Q8IYB3	SRRM1	532	S	VGRRRRHSPSRSASPSPRKRQKETSPRGRRR	4.61
575	Q9BW71	HIRIP3	357	S	GEPTAKGSRKMARLGSTSGEESDLEREVSDS	4.56
576	Q9NYF8	BCLAF1	198	S	SQEEPKDTFEHDPSESIDEFNKSSATSGDIW	4.46
577	O15027	SEC16A	164	S	HRPASALVNPLARGDSPENRTHHPLGAGAGS	4.43
578	Q9UQ35	SRRM2	782	S	SKAKSRLSLRRSLSGSSPCPKQKSQTPPRRS	4.42
579	Q15185	PTGES3	148	S	DEDVDLPEVDGADDDSQDSDDEKMPDLE***	4.41
580	Q6ZMG9	CERS6	346	S	SRGKVSKDDRSDIESSSDEEDSEPPGKNPHT	4.35
581	Q96IZ7	RSRC1	118	S	SRTRRSRSRPRLRSHSRSSERSSHRRTRSRS	4.29
582	Q9BW71	HIRIP3	87	S	KLDLTKKGKRPPTPCSDPERKRFRFNSESES	4.28
583	Q9UKJ3	GPATCH8	349	S	PDEKSSDQGLQKVGDSDGSSNLDGKKEDEDP	4.27
584	Q13428	TCOF1	934	S	TGPSAAQAGKQDDSGSSSEESDSDGEAPAAV	4.25
585	Q96ST2	IWS1	440	S	VSDKSGKREKTIASDSEEEAGKELSDKKNEE	4.15
586	Q9BWU0	SLC4A1AP	466	S	RKRKAKNWEDEDFYDSDDDTFLDRTGLIEKK	4.12
587	Q9C0C2	TNKS1BP1	1620	S	HLFQDSTEPRASRVPSSDEEVVEEPQSRRTR	4.01
588	P43487	RANBP1	60	S	EEELFKMRAKLFRFASENDLPEWKERGTGDV	3.92
589	Q8IYB3	SRRM1	528	S	KNGEVGRRRRHSPSRSASPSPRKRQKETSPR	3.92
590	Q13428	TCOF1	346	S	KAGKPEEDSESSSEESSDSEEETPAAKALLQ	3.88
591	Q92769	HDAC2	394	S	GVQMQAIPEDAVHEDSGDEDGEDPDKRISIR	3.84
592	O15042	U2SURP	800	S	HEESEEEENQNQEEESEDEEDTQSSKSEEHH	3.83
593	P49959	MRE11A	689	S	IMSQSQVSKGVDFESSEDDDDDPFMNTSSLR	3.82
594	Q99590	SCAF11	830	S	QSPSPRRETGKESRKSQSPSPKNESARGRKK	3.81
595	Q9UQ35	SRRM2	248	S	TPKSKRKSKDKKRKRSRSTTPAPKSRRAHRS	3.80
596	O95382	MAP3K6	912	S	AQTLLGDPFLQPGKRSRSPSSPRHAPRPSDA	3.78
597	P07910	HNRNPC	260	S	DETNVKMESEGGADDSAEEGDLLDDDDNEDR	3.75
598	Q96ST2	IWS1	398	S	EGEEEKVAKRKAAVLSDSEDEEKASAKKSRV	3.74
599	Q9UQL6	HDAC5	611	S	EEEEDCIQVKDEEGESGAEEGPDLEEPGAGY	3.73
600	Q9UQ35	SRRM2	745	S	RKNKSRTSQRRSRSNSSPEMKKSRISSRRSR	3.71
601	Q9Y5S9	RBM8A	168	S	GPPKGKRRGGRRRSRSPDRRRR*********	3.69
602	P35269	GTF2F1	221	S	RIHDLEDDLEMSSDASDASGEEGGRVPKAKK	3.68
603	P78317	RNF4	95	S	ARRLPQDHADSCVVSSDDEELSRDRDVYVTI	3.67
604	Q92625	ANKS1A	661	S	ESLSNCSIGKKRLEKSPSFASEWDEIEKIMS	3.66
605	Q99590	SCAF11	834	S	PRRETGKESRKSQSPSPKNESARGRKKSRSQ	3.65
606	Q9UKN8	GTF3C4	19	S	ADQARVGPADDGPAPSGEEEGEGGGEAGGKE	3.64
607	Q9UQ35	SRRM2	715	S	GRSRSRSLVRRGRSHSRTPQRRGRSGSSSER	3.63
608	P35579	MYH9	1943	S	FVVPRRMARKGAGDGSDEEVDGKADGAEAKP	3.62
609	P42166	TMPO	67	S	PLPAGTNSKGPPDFSSDEEREPTPVLGSGAA	3.61
610	Q08945	SSRP1	672	S	SESFKSKEFVSSDESSSGENKSKKKRRRSED	3.60
611	Q15459	SF3A1	359	S	SQLDQDTQVQDMDEGSDDEEEGQKVPPPPET	3.60
612	Q66PJ3	ARL6IP4	332	S	QVEALPGPSLDQWHRSAGEEEDGPVLTDEQK	3.59
613	Q86US8	SMG6	1196	S	DVVIEDFEEDSEAEGSGGEDDIRELRAKKLA	3.58
614	Q8TDR0-2	TRAF3IP1	359	S	TSKRRSKNSVEGDSTSDAEGDAGPAGQDKSE	3.57
615	Q96JC9	EAF1	165	S	KPPVGPKTSPLKDNPSPEPQLDDIKRELRAE	3.56
616	Q9H792	PEAK1	730	S	RGQSSPQRSYSSSHSSPAKIQRATQEPVAKI	3.55
617	O15021	MAST4	1831	S	PQASKTELPSPESAQSPSPSGDVRASVPPVL	3.54
618	Q9H0E3	SAP130	300	S	IPPAVVATVSATRAQSPVITTTAAHATDSAL	3.54
619	Q9Y2X3	NOP58	502	S	KKKRGKKKHIKEEPLSEEEPCTSTAIASPEK	3.53
620	P08621	SNRNP70	320	S	RKEELRGGGGDMAEPSEAGDAPPDDGPPGEL	3.53
621	Q9NXX6	NSMCE4A	35	S	HRDRTRSRSRSRSPLSPRSRRGSARERREAP	3.52
622	Q58EX2	SDK2	2026	S	YTRSPPRPSPGSLHYSDEDVTKYNDLIPAES	3.52
623	P18583	SON	1782	S	AASPVVSSMPERASESSSEEKDDYEIFVKVK	3.52
624	P19338	NCL	145	S	PAKGAKNGKNAKKEDSDEEEDDDSEEDEEDD	3.52
625	Q9UQ35	SRRM2	454	S	ESPKPAPAPGSHREISSSPTSKNRSHGRAKR	3.51

626	P19338	NCL	153	S	KNAKKEDSDEEEDDDSEEDEEDDEDEDEDED	3.50
627	Q9UKJ3	GPATCH8	491	S	PGSKAEAKKALGGDVSDQSLESHSQKVSETQ	3.49
628	P85037	FOXK1	249	S	LRSMVSPVPSPTGTISVPNSCPASPRGAGSS	3.48
629	P98175	RBM10	738	S	SPPRGLVAAYSGESDSEEEQERGGPEREEKI	3.47
630	Q9UPP1	PHF8	854	S	DSLGACFKDAEYIYPSLESDDDDPALKSRPK	3.46
631	Q9UQ35	SRRM2	952	S	SPSRVTSRTTPRRSRSVSPCSNVESRLLPRY	3.46
632	Q15154	PCM1	65	S	KKFGVESDKRVTNDISPESSPGVGRRRTKTP	3.45
633	Q13428	TCOF1	583	S	QEKSLGNILQAKPTSSPAKGPPQKAGPVAVQ	3.44
634	Q14677	CLINT1	234	S	FRRKDREDSPERCSDSDEEKKARRGRSPKGE	3.44
635	Q96ST2	IWS1	289	S	SDSESEDPPRNQASDSENEELPKPRVSDSES	3.43
636	Q9UQ35	SRRM2	746	S	KNKSRTSQRRSRSNSSPEMKKSRISSRRSRS	3.42
637	P51003	PAPOLA	24	S	GSQQTQPPQKHYGITSPISLAAPKETDCVLT	3.42
638	P98174	FGD1	48	S	SDPGASEPGLLARRGSGSALGGPLDPQFVGP	3.42
639	O43432-3	EIF4G3	232	S	KQEEKPKPDPVLKSPSPVLRLVLSGEKKEQE	3.41
640	O15541	RNF113A	46	S	RKRPACDPEPGESGSSSDEGCTVVRPEKKRV	3.40
641	Q14684	RRP1B	458	S	VAEPGAEATSSTGEESGSEHPPAVPMHNKRK	3.39
642	P36507	MAP2K2	293	S	IFGRPVVDGEEGEPHSISPRPRPPGRPVSGH	3.37
643	Q9P219	CCDC88C	227	S	DLTQERDYLQAQHPPSPIKSSSADSTPSPTS	3.36
644	Q6AWC2	WWC2	1022	S	PGERNQYICRLNRSDSDSSTLAKKSLFVRNS	3.33
645	Q9BVV6	KIAA0586	376	S	RGNVRLLEQILNNNDSLTRKSESSNTTSLTR	3.29
646	P46937	YAP1	164	S	SGPAATPTAQHLRQSSFEIPDDVPLPAGWEM	3.29
647	P85037	FOXK1	243	S	HIPEPDLRSMVSPVPSPTGTISVPNSCPASP	3.28
648	P46100	ATRX	1352	S	RHRLLRHKLTVSDGESGEEKKTKPKEHKEVK	3.27
649	Q7L4I2	RSRC2	222	S	IEKPRRFSRSLSRTPSPPPFRGRNTAMDAQE	3.26
650	O95218	ZRANB2	153	S	AVGPASILKEVEDKESEGEEEDEDEDLSKYK	3.25
651	Q01831	XPC	884	S	SEAAAPHTDAGGGLSSDEEEGTSSQAEAARI	3.25
652	Q13428	TCOF1	1153	S	KAPESSDDSEDSSDSSSGSEEDGEGPQGAKS	3.25
653	Q8IYB3	SRRM1	874	S	QEEPVAAPEPKKETESEAEDNLDDLEKHLRE	3.25
654	Q9UQ35	SRRM2	743	S	SERKNKSRTSQRRSRSNSSPEMKKSRISSRR	3.25
655	P29692	EEF1D	162	S	TPAEDDEDDDIDLFGSDNEEEDKEAAQLREE	3.25
656	Q86US8	SMG6	1191	S	EDEEEDVVIEDFEEDSEAEGSGGEDDIRELR	3.24
657	Q8N5S9	CAMKK1	458	S	SWTTVILVKSMLRKRSFGNPFEPQARREERS	3.24
658	Q92882	OSTF1	213	S	VRTLSNAEDYLDDEDSD**************	3.23
659	Q5BKY9	FAM133B	191	S	GLSKKRKMYSEDKPLSSESLSESEYIEEVRA	3.22
660	Q6UN15	FIP1L1	89	S	PKPKVTETEDDSDSDSDDDEDDVHVTIGDIK	3.21
661	Q96ST2	IWS1	315	S	SDSESEGPQKGPASDSETEDASRHKQKPESD	3.21
662	O00567	NOP56	520	S	KPKKKKSFSKEELMSSDLEETAGSTSIPKRK	3.20
663	O94842	TOX4	178	S	LPPAQSPEDRLSTTPSPTSSLHEDGVEDFRR	3.20
664	P53999	SUB1	17	S	PKSKELVSSSSSGSDSDSEVDKKLKRKKQVA	3.19
665	P67809	YBX1	314	S	DGKETKAADPPAENSSAPEAEQGGAE*****	3.19
666	P78317	RNF4	94	S	NARRLPQDHADSCVVSSDDEELSRDRDVYVT	3.19
667	Q16629	SRSF7	204	S	YFQSPSRSRSRSRSISRPRSSRSKSRSPSPK	3.19
668	Q5BKY9	FAM133B	82	S	ENWKKELEKHREKLLSGSESSSKKRQRKKKE	3.19
669	Q9UQ35	SRRM2	566	S	RGRSRSARRGRSHSRSPATRGRSRSRTPARR	3.18
670	Q96ST2	IWS1	313	S	RVSDSESEGPQKGPASDSETEDASRHKQKPE	3.18
671	Q9UQ35	SRRM2	573	S	RRGRSHSRSPATRGRSRSRTPARRGRSRSRT	3.18
672	Q5UIP0	RIF1	1454	S	QKKERRKEEEKPLQKSPLHIKDDVLPKQKLI	3.17
673	Q86X95	CIR1	202	S	GRNLTANDPSQEYVASEGEEDPEVEFLKSLT	3.16
674	O14654	IRS4	1231	S	PPRSRRVPRPPEREDSDNDDDTHVRMDFARR	3.15
675	P51825	AFF1	206	S	DSAPERELSPLISLPSPVPPLSPIHSNQQTI	3.15
676	Q13428	TCOF1	967	S	AQVIKPPLIFVDPNRSPAGPAATPAQAQAAS	3.15
677	Q9BW71	HIRIP3	159	S	DEERQRDLPAQRGEESSEEEEKGYKGKTRKK	3.15
678	Q9UDT6	CLIP2	294	S	FAPIHKVIRIGFPSTSPAKAKKTKRMAMGVS	3.15
679	Q9UGP8	SEC63	576	S	AVKEDEEEVSDKGSDSEEEETNRDSQSEKDD	3.15
680	P23588	EIF4B	424	S	SEETQERERSRTGSESSQTGTSTTSSRNARR	3.15
681	Q9UKM9	RALY	135	S	RDDFYDRLFDYRGRLSPVPVPRAVPVKRPRV	3.15
682	P25788	PSMA3	250	S	EAEKYAKESLKEEDESDDDNM**********	3.14

683	Q01130	SRSF2	189	S	SSVSRSRSRSRSRSRSRSPPPVSKRESKSRS	3.14
684	Q08945	SSRP1	671	S	LSESFKSKEFVSSDESSSGENKSKKKRRRSE	3.14
685	Q9C0C2	TNKS1BP1	1666	S	LSPSALKAKLRPRNRSAEEGELAESKSSQKE	3.14
686	Q9UQ35	SRRM2	575	S	GRSHSRSPATRGRSRSRTPARRGRSRSRTPA	3.14
687	O43524	FOXO3	253	S	PDGGKSGKAPRRRAVSMDNSNKYTKSRGRAA	3.14
688	O95831	AIFM1	116	S	GLGLTPEQKQKKAALSASEGEEVPQDKAPSH	3.14
689	P12268	IMPDH2	416	S	FFSDGIRLKKYRGMGSLDAMDKHLSSQNRYF	3.14
690	Q6P6C2	ALKBH5	361	S	ENRRSVLLPTHRRRGSFSSENYWRKSYESSE	3.14
691	Q8IYB3	SRRM1	616	S	QRRYSPSPPPKRRTASPPPPPKRRASPSPPP	3.13
692	Q96ST2	IWS1	287	S	RISDSESEDPPRNQASDSENEELPKPRVSDS	3.12
693	O14654	IRS4	1107	S	FFAAARAAVSAFPTDSLERDLSPSSAPAVAS	3.11
694	P18583	SON	1784	S	SPVVSSMPERASESSSEEKDDYEIFVKVKDT	3.11
695	P21127	CDK11B	283	S	EKMEERDLLSDLQDISDSERKTSSAESSSAE	3.10
696	P51114	FXR1	413	S	GYGTNSELSNPSETESERKDELSDWSLAGED	3.10
697	P53999	SUB1	13	S	***MPKSKELVSSSSSGSDSDSEVDKKLKRK	3.07
698	P85037	FOXK1	257	S	PSPTGTISVPNSCPASPRGAGSSSYRFVQNV	3.05
699	P98175	RBM10	61	S	REYGSQEGKHDYDDSSEEQSAEDSYEASPGS	3.05
700	Q13428	TCOF1	548	S	TPSAQVGKWEEDSESSSEESSDSSDGEVPTA	3.04
701	Q2NKX8	ERCC6L	1028	S	VVVKAKIRSKARRIVSDGEDEDDSFKDTSSI	3.02
702	Q7Z4V5	HDGFRP2	299	S	LPKPRGRKPKPERPPSSSSSDSDSDEVDRIS	3.02
703	Q96ST2	IWS1	511	S	LEEEKGETQVKEAEDSDSDDNIKRGKHMDFL	2.99
704	Q9BW71	HIRIP3	160	S	EERQRDLPAQRGEESSEEEEKGYKGKTRKKP	2.98
705	Q9NR09	BIRC6	480	S	DIPKLEGDSDDLLEDSDSEEHSRSDSVTGHT	2.97
706	Q9UGP8	SEC63	574	S	EAAVKEDEEEVSDKGSDSEEEETNRDSQSEK	2.97
707	Q9UPT8	ZC3H4	92	S	GGPERSRKEKGEKHHSDSDEEKSHRRLKRKR	2.97
708	Q9UQ35	SRRM2	629	S	RSRSRTPARRRSRTRSPVRRRSRSRSPARRS	2.96
709	Q9Y4F5	CEP170B	1135	S	PTRASRLRRARLGDASDTEAADGERGSLGNP	2.95
710	Q96ST2	IWS1	167	S	GKHPASDSEIEELQKSPASDSETEDALKPQI	2.94
711	Q04727	TLE4	269	S	LVVDVSNEDPSSPRGSPAHSPRENGLDKTRI	2.94
712	Q07157	TJP1	277	S	RDERATLLNVPDLSDSIHSANASERDDISEI	2.94
713	Q13428	TCOF1	941	S	AGKQDDSGSSSEESDSDGEAPAAVTSAQVIK	2.94
714	Q53EL6	PDCD4	78	S	LRKNSSRDSGRGDSVSDSGSDALRSGLTVPT	2.93
715	Q6UN15	FIP1L1	87	S	GVPKPKVTETEDDSDSDSDDDEDDVHVTIGD	2.92
716	Q7L1Q6	BZW1	413	S	KKFVEWLKNAEEESESEAEEGD*********	2.92
717	Q7LBC6	KDM3B	822	S	RQDSDSSTNSDLSDLSDSEEQLQAKTGLKGI	2.91
718	Q92974	ARHGEF2	947	S	SPEERLQDSSDPDTGSEEEGSSRLSPPHSPR	2.91
719	Q9HCK8	CHD8	2071	S	RSVPPVKLEDEDDSDSELDLSKLSPSSSSSS	2.91
720	Q9NVU0	POLR3E	162	S	AKHREREAANEAGDSSQDEAEDDVKQITVRF	2.90
721	Q9Y6M1	IGF2BP2	161	S	ENYSFKISYIPDEEVSSPSPPQRAQRGDHSS	2.89
722	O00567	NOP56	569	S	SGSKKKRKFSKEEPVSSGPEEAVGKSSSKKK	2.89
723	P49959	MRE11A	688	S	KIMSQSQVSKGVDFESSEDDDDDPFMNTSSL	2.88
724	Q15345	LRRC41	145	S	EAFFSHVLRGTIDVSSDRRLCDQRFSPLLHS	2.87
725	O75179	ANKRD17	2047	S	PTAKEHYPVSSPSSPSPPAQPGGVSRNSPLD	2.87
726	P51114	FXR1	409	S	NYTSGYGTNSELSNPSETESERKDELSDWSL	2.87
727	Q06587	RING1	229	S	GTGGGGTGGVGGGAGSEDSGDRGGTLGGGTL	2.86
728	Q9NVU0	POLR3E	161	S	DAKHREREAANEAGDSSQDEAEDDVKQITVR	2.85
729	Q13428	TCOF1	342	S	ASQTKAGKPEEDSESSSEESSDSEEETPAAK	2.84
730	Q9UDY2	TJP2	1159	S	KAPSRPYQDTRGSYGSDAEEEEYRQQLSEHS	2.84
731	Q9Y2U5	MAP3K2	163	S	RKKRLSIIGPTSRDRSSPPPGYIPDELHQVA	2.84
732	Q8TF01	PNISR	408	S	SGDSEDERSDRGSESSDTDDEELRHRIRQKQ	2.84
733	O60832	DKC1	453	S	VAEAAKTAKRKRESESESDETPPAAPQLIKK	2.84
734	O95218-2	ZRANB2	310	S	SGDRKKRRTRSRSPESQVIGENTKQP*****	2.84
735	P46100	ATRX	1442	S	RIKVQEDSSSENKSNSEEEEEEKEEEEEEEE	2.83
736	Q6PKG0	LARP1	631	S	MDGRKNTFTAWSDEESDYEIDDRDVNKILIV	2.83
737	Q7Z406	MYH14	1969	S	TRTVRQVFRLEEGVASDEEAEEAQPGSGPSP	2.83
738	Q92974	ARHGEF2	941	S	ERQELGSPEERLQDSSDPDTGSEEEGSSRLS	2.82
739	Q96ST2	IWS1	172	S	SDSEIEELQKSPASDSETEDALKPQISDSES	2.81

740	Q9C0C2	TNKS1BP1	1621	S	LFQDSTEPRASRVPSSDEEVVEEPQSRRTRM	2.81
741	Q9HAS0	C17orf75	18	S	PSLQESMDGDEKELESSEEGGSAEERRLEPP	2.81
742	Q9UQ35	SRRM2	713	S	RRGRSRSRSLVRRGRSHSRTPQRRGRSGSSS	2.81
743	Q9UQ35	SRRM2	455	S	SPKPAPAPGSHREISSSPTSKNRSHGRAKRD	2.80
744	O96007	MOCS2	8	S	********MSSLEISSSCFSLETKLPLSPPL	2.80
745	P18583	SON	1931	S	KTVRARSRTPSRRSRSHTPSRRRRSRSVGRR	2.80
746	Q01130	SRSF2	220	S	RSKSPPKSPEEEGAVSS**************	2.80
747	Q92769	HDAC2	424	S	RASDKRIACDEEFSDSEDEGEGGRRNVADHK	2.80
748	Q9UKJ3	GPATCH8	353	S	SSDQGLQKVGDSDGSSNLDGKKEDEDPQDGG	2.79
749	Q9UPT8	ZC3H4	94	S	PERSRKEKGEKHHSDSDEEKSHRRLKRKRKK	2.78
750	Q9UQ35	SRRM2	954	S	SRVTSRTTPRRSRSVSPCSNVESRLLPRYSH	2.77
751	Q9Y3T9	NOC2L	672	S	KDEDRKQFKDLFDLNSSEEDDTEGFSERGIL	2.76
752	P53999	SUB1	15	S	*MPKSKELVSSSSSGSDSDSEVDKKLKRKKQ	2.76
753	Q9BW61	DDA1	95	S	EGESSAPPRKVARTDSPDMHEDT********	2.75
754	Q9BWU0	SLC4A1AP	82	S	SQDLSGDFKKPALPVSPAARSKAPASSSSNP	2.75
755	Q9P206	KIAA1522	669	S	TLTPLQESPVISKDQSPPPSPPPSYHPPPPP	2.74
756	O15021	MAST4	1825	S	IALLSGPQASKTELPSPESAQSPSPSGDVRA	2.73
757	Q8IYB3	SRRM1	465	S	SPSPAPKPRKVELSESEEDKGGKMAAADSVQ	2.72
758	Q9UGP8	SEC63	570	S	VVGNEAAVKEDEEEVSDKGSDSEEEETNRDS	2.71
759	P55081	MFAP1	52	S	KRYVSGKRPDYAPMESSDEEDEEFQFIKKAK	2.71
760	Q5M9Q1	NKAPL	140	S	RERIGELGAPEVWGPSPKFPQLDSDEHTPVE	2.71
761	Q9UPP1	PHF8	857	S	GACFKDAEYIYPSLESDDDDPALKSRPKKKK	2.71
762	P46783	RPS10	146	S	AVPPGADKKAEAGAGSATEFQFRGGFGRGRG	2.69
763	Q01130	SRSF2	191	S	VSRSRSRSRSRSRSRSPPPVSKRESKSRSRS	2.68
764	Q01831	XPC	883	S	KSEAAAPHTDAGGGLSSDEEEGTSSQAEAAR	2.67
765	Q15345	LRRC41	144	S	MEAFFSHVLRGTIDVSSDRRLCDQRFSPLLH	2.67
766	Q99871-2	HAUS7	391	S	RSGMLLQVVMAVADTSAKAVETVKKQQGEQI	2.67
767	Q8IYB3	SRRM1	560	S	RRRRSPSPPPTRRRRSPSPAPPPRRRRTPTP	2.67
768	Q96DR7	ARHGEF26	222	S	QKSSSEQKLPLQRLPSQENELLENPSVVLST	2.67
769	Q96ST2	IWS1	27	S	DDGGATPVQDERDSGSDGEDDVNEQHSGSDT	2.67
770	P35269	GTF2F1	224	S	DLEDDLEMSSDASDASGEEGGRVPKAKKKAP	2.66
771	P18583	SON	2009	S	TPSRRRRSRSVVRRRSFSISPVRLRRSRTPL	2.65
772	P55081	MFAP1	53	S	RYVSGKRPDYAPMESSDEEDEEFQFIKKAKE	2.65
773	Q7L014	DDX46	804	S	NERKKLQKAALGLQDSDDEDAAVDIDEQIES	2.65
774	Q9H1B7	IRF2BPL	215	S	PKPTPEEGPPELNRQSPNSSSAAASVASRRG	2.65
775	Q01130	SRSF2	208	S	PPVSKRESKSRSRSKSPPKSPEEEGAVSS**	2.65
776	Q13428	TCOF1	341	S	VASQTKAGKPEEDSESSSEESSDSEEETPAA	2.64
777	Q8WUB8	PHF10	331	S	KGTSDSSSGNVSEGESPPDSQEDSFQGRQKS	2.64
778	Q08J23	NSUN2	456	S	KLQGKSAETRESTQLSPADLTEGKPTDPSKL	2.64
779	Q13428	TCOF1	939	S	AQAGKQDDSGSSSEESDSDGEAPAAVTSAQV	2.64
780	Q13523	PRPF4B	580	S	SEPSSPQSSTRTRSPSPDDILERVAADVKEY	2.64
781	Q14191	WRN	1133	S	ISSGSNISKKSIMVQSPEKAYSSSQPVISAQ	2.63
782	Q14498	RBM39	341	S	VTERTDASSASSFLDSDELERTGIDLGTTGR	2.62
783	Q16629	SRSF7	209	S	SRSRSRSRSISRPRSSRSKSRSPSPKRSRSP	2.61
784	Q4VCS5	AMOT	1041	S	EVPASPATGPGPHRLSIPSLTCNPDKTDGPV	2.61
785	Q5BKY9	FAM133B	192	S	LSKKRKMYSEDKPLSSESLSESEYIEEVRAK	2.61
786	Q6PD62	CTR9	1041	S	LKIADEGHPRNSNSNSDSDEDEQRKKCASSE	2.61
787	Q6UN15	FIP1L1	85	S	ENGVPKPKVTETEDDSDSDSDDDEDDVHVTI	2.61
788	Q7L2J0	MEPCE	152	S	QPHRPPGGGGGKRRNSCNVGGGGGGFKHPAF	2.61
789	Q7Z2Z1	TICRR	1750	S	LEDFELEGVCQLPDQSPPRNSMPKAEEASSW	2.61
790	Q7Z6E9	RBBP6	1644	S	TRETDEAAFEPDYNESDSESNVSVKEEESSG	2.61
791	Q86VM9	ZC3H18	32	S	EEQPQGLSDDDILRDSGSDQDLDGAGVRASD	2.60
792	Q96I25	RBM17	155	S	DRHEASGFARRPDPDSDEDEDYERERRKRSM	2.59
793	Q96ST2	IWS1	252	S	SENEELPKPRISDSESEDPPRHQASDSENEE	2.58
794	Q96ST3	SIN3A	832	S	HHFIPDLLFAQRGDLSDVEEEEEEEMDVDEA	2.58
795	Q99590	SCAF11	832	S	PSPRRETGKESRKSQSPSPKNESARGRKKSR	2.58
796	Q9Y5S9	RBM8A	166	S	VRGPPKGKRRGGRRRSRSPDRRRR*******	2.58

797	Q9BW71	HIRIP3	359	S	PTAKGSRKMARLGSTSGEESDLEREVSDSEA	2.57
798	O60832	DKC1	451	S	QVVAEAAKTAKRKRESESESDETPPAAPQLI	2.57
799	Q8N5F7	NKAP	23	S	RSPDREASGSGGRRRSSSKSPKPSKSARSPR	2.57
800	Q01130	SRSF2	206	S	SPPPVSKRESKSRSRSKSPPKSPEEEGAVSS	2.57
801	P55198	MLLT6	258	S	KDKERLKQKHKKRPESPPSILTPPVVPTADK	2.57
802	Q12923	PTPN13	1321	S	KPGISDVTDYSDRGDSDMDEATYSSSQDHQT	2.56
803	Q13428	TCOF1	547	S	ATPSAQVGKWEEDSESSSEESSDSSDGEVPT	2.56
804	Q13428	TCOF1	936	S	PSAAQAGKQDDSGSSSEESDSDGEAPAAVTS	2.56
805	Q9BW71	HIRIP3	363	S	GSRKMARLGSTSGEESDLEREVSDSEAGGGP	2.56
806	Q8IXM2	BAP18	35	S	TKLGELTMQLHPVADSSPAGAKWTETEIEML	2.55
807	Q8TAP9	MPLKIP	133	S	TSTPFGSGRVREKRMSNELENYFKPSMLEDP	2.54
808	Q9NVU7	SDAD1	585	S	AAPGKSQKRKYIEIDSDEEPRGELLSLRDIE	2.54
809	Q8IXT5	RBM12B	278	S	HFRKRSHSKSPRRTRSRSPLGFYVHLKNLSI	2.54
810	Q6PD62	CTR9	1102	S	SDQPSRKRRPSGSEQSDNESVQSGRSHSGVS	2.53
811	Q05519	SRSF11	434	S	KQVTRDYDEEEQGYDSEKEKKEEKKPIETGS	2.53
812	Q8N9T8	KRI1	97	S	QKDATFYNRTASSSDSEEDPEALEKQKKVRP	2.53
813	Q13428	TCOF1	343	S	SQTKAGKPEEDSESSSEESSDSEEETPAAKA	2.52
814	P52948	NUP98	888	S	WVFKVSHFSKYGLQDSDEEEEEHPSKTSTKK	2.51
815	Q96ST2	IWS1	377	S	SSDSEEEEHKKQKMDSDEDEKEGEEEKVAKR	2.51
816	Q9H019	MTFR1L	237	S	CSSSEEDDCVSLSKASSFADMMGILKDFHRM	2.50
817	Q9HCE7	SMURF1	106	S	GAGFLGCVRLLSNAISRLKDTGYQRLDLCKL	2.50
818	P04637	TP53	33	S	TFSDLWKLLPENNVLSPLPSQAMDDLMLSPD	2.50
819	P18583	SON	1935	S	ARSRTPSRRSRSHTPSRRRRSRSVGRRRSFS	2.50
820	P27815-7	PDE4A	6	S	**********MKRSRSALSVAGTGDERSRET	2.49
821	Q9UQ35	SRRM2	495	S	RMGRSRSPATAKRGRSRSRTPTKRGHSRSRS	2.49
822	Q8WUB8	PHF10	327	S	KRKNKGTSDSSSGNVSEGESPPDSQEDSFQG	2.48
823	O00567	NOP56	519	S	SKPKKKKSFSKEELMSSDLEETAGSTSIPKR	2.48
824	Q9HCK8	CHD8	2069	S	VSRSVPPVKLEDEDDSDSELDLSKLSPSSSS	2.47
825	Q9Y2X7	GIT1	388	S	SQSDLDDQHDYDSVASDEDTDQEPLRSTGAT	2.47
826	Q13428	TCOF1	545	S	GPATPSAQVGKWEEDSESSSEESSDSSDGEV	2.47
827	Q13428	TCOF1	932	S	GKTGPSAAQAGKQDDSGSSSEESDSDGEAPA	2.46
828	Q9NUQ3	TXLNG	105	S	TENRNLVSPAYCTQESREEIPGGEARTDPPD	2.46
829	Q14684	RRP1B	245	S	ETMEEQKTKVGDGDLSAEEIPENEVSLRRAV	2.45
830	O60832	DKC1	494	S	AKAGLESGAEPGDGDSDTTKKKKKKKKAKEV	2.45
831	Q01130	SRSF2	204	S	SRSPPPVSKRESKSRSRSKSPPKSPEEEGAV	2.45
832	Q01804	OTUD4	1024	S	ANSVDSRVQRPKEESSEDENEVSNILRSGRS	2.44
833	P35611-3	ADD1	358	S	LLNPEKYKAKSRSPGSPVGEGTGSPPKWQIG	2.44
834	Q01804	OTUD4	1023	S	GANSVDSRVQRPKEESSEDENEVSNILRSGR	2.44
835	P50502	ST13	79	S	KVEEDLKADEPSSEESDLEIDKEGVIEPDTD	2.43
836	Q99759-2	MAP3K3	175	S	PRSRHLSVSSQNPGRSSPPPGYVPERQQHIA	2.42
837	Q16629	SRSF7	208	S	PSRSRSRSRSISRPRSSRSKSRSPSPKRSRS	2.42
838	Q7Z4V5	HDGFRP2	190	S	SDLDQASVSPSEEENSESSSESEKTSDQDFT	2.42
839	Q13523	PRPF4B	578	S	VPSEPSSPQSSTRTRSPSPDDILERVAADVK	2.41
840	Q9Y2W1	THRAP3	55	S	SRKRRLSSRSRSRSYSPAHNRERNHPRVYQN	2.41
841	Q9UQ35	SRRM2	564	S	QRRGRSRSARRGRSHSRSPATRGRSRSRTPA	2.41
842	Q9UQ35	SRRM2	562	S	NTQRRGRSRSARRGRSHSRSPATRGRSRSRT	2.40
843	O15541	RNF113A	43	S	AGRRKRPACDPEPGESGSSSDEGCTVVRPEK	2.40
844	P16989	YBX3	369	S	AGEAPTENPAPPTQQSSAE************	2.39
845	Q9UQ35	SRRM2	456	S	PKPAPAPGSHREISSSPTSKNRSHGRAKRDK	2.38
846	O75494	SRSF10	131	S	YRRSRSRSYERRRSRSRSFDYNYRRSYSPRN	2.38
847	P16383	GCFC2	119	S	HHSSESKDDQGLSSDSSSSLGEKELSSTVKI	2.37
848	Q9NYV4	CDK12	332	S	GSYSGRSPSPYGRRRSSSPFLSKRSLSRSPL	2.37
849	Q9Y2W2	WBP11	237	S	ALDLPPRRRDEDMLYSPELAQRGHDDDVSST	2.37
850	Q9NYV4	CDK12	333	S	SYSGRSPSPYGRRRSSSPFLSKRSLSRSPLP	2.36
851	Q8IZ21	PHACTR4	270	S	AKQPPIPPPKPAHRNSNPVIAELSQAINSGI	2.35
852	Q9UPU7	TBC1D2B	957	S	FLRERDTSPDKGELVSDEEEDT*********	2.35
853	Q14247	CTTN	438	S	DAASFKAELSYRGPVSGTEPEPVYSMEAADY	2.34

854	Q8WX93	PALLD	1121	S	SGHPHVRRPRSRSRDSGDENEPIQERFFRPH	2.34
855	P46100	ATRX	1348	S	KPRYRHRLLRHKLTVSDGESGEEKKTKPKEH	2.27
856	Q13428	TCOF1	549	S	PSAQVGKWEEDSESSSEESSDSSDGEVPTAV	2.24
857	P52701	MSH6	287	S	EGSSDEISSGVGDSESEGLNSPVKVARKRKR	2.24
858	Q8NDI1	EHBP1	174	S	LREGKATDEDMQSLASLMSMKQADIGNLDDF	2.23
859	Q04637	EIF4G1	1596	S	SVTAFFKWLREAEEESDHN************	2.19
860	Q6PD62	CTR9	1043	S	IADEGHPRNSNSNSDSDEDEQRKKCASSESD	2.16
861	Q96CB8	INTS12	378	S	PPPLTLGKTGLSRSVSCDNVSKVGLPSPSSL	2.16
862	Q96ST2	IWS1	25	S	QSDDGGATPVQDERDSGSDGEDDVNEQHSGS	2.16
863	P16383	GCFC2	117	S	KIHHSSESKDDQGLSSDSSSSLGEKELSSTV	2.15
864	P51858	HDGF	133	S	AAEGDGDKKGNAEGSSDEEGKLVIDEPAKEK	2.15
865	O60832	DKC1	485	S	KKKSKKDKKAKAGLESGAEPGDGDSDTTKKK	2.14
866	P18583	SON	1929	S	NRKTVRARSRTPSRRSRSHTPSRRRRSRSVG	2.14
867	Q8TF01	PNISR	286	S	TEDAEGGDGPRLPQRSKFDSDEEEEDTENVE	2.14
868	Q9Y3T9	NOC2L	673	S	DEDRKQFKDLFDLNSSEEDDTEGFSERGILR	2.13
869	O95382	MAP3K6	914	S	TLLGDPFLQPGKRSRSPSSPRHAPRPSDAPS	2.12
870	O00567	NOP56	570	S	GSKKKRKFSKEEPVSSGPEEAVGKSSSKKKK	2.11
871	Q8TF01	PNISR	396	S	SLTGLGGLGGYGSGDSEDERSDRGSESSDTD	2.04
872	P18583	SON	1783	S	ASPVVSSMPERASESSSEEKDDYEIFVKVKD	2.04
873	Q5T3I0	GPATCH4	130	S	LTSGGEKPNKDLESCSDDDNQGSKSPKILTD	2.04
874	Q96IZ7	RSRC1	116	S	SKSRTRRSRSRPRLRSHSRSSERSSHRRTRS	2.04
875	Q9NPQ8	RIC8A	436	S	AARGLMAGGRPEGQYSEDEDTDTDEYKEAKA	2.00
876	Q9Y2W1	THRAP3	743	S	KERDLKRGKSRESVDSRDSSHSRERSAEKTE	2.00
877	Q01433	AMPD2	188	T	LEPDILLRAKQDFLKTDSDSDLQLYKEQGEG	1769.91
878	O75400	PRPF40A	932	T	KSPKKKTGKDSGNWDTSGSELSEGELEKRRR	1140.61
879	Q9UQ35	SRRM2	1927	T	RSRASPVSRRRSRSRTPPVTRRRSRSRTPTT	786.63
880	Q3YEC7	RABL6	599	T	RRADDFPVRDDPSDVTDEDEGPAEPPPPPKL	570.30
881	P25205	MCM3	674	T	EKEKKRKKRSEDESETEDEEEKSQEDQEQKR	550.64
882	Q6QNY0	BLOC1S3	63	T	RGRPTGLRVAGEAAETDSEPEPEPEPTAAPR	452.31
883	Q6PD62	CTR9	925	T	RSKKGGEFDEFVNDDTDDDLPISKKKKRRKG	393.31
884	Q4G0J3	LARP7	338	T	EASEASKENRDIEISTEEEKDTGDLKDSSLI	334.32
885	Q9NU22	MDN1	4898	T	DDLNLDSEDKNGGEDTDNEEGEEENPLEIKE	324.37
886	P55081	MFAP1	267	T	LEENKRSLAALDALNTDDENDEEEYEAWKVR	314.65
887	Q13427	PPIG	358	T	TPSRSRSRDRFRRSETPPHWRQEMQRAQRMR	255.65
888	P25205	MCM3	713	T	DAKDGDSYDPYDFSDTEEEMPQVHTPKTADS	235.99
889	Q8IZL8	PELP1	1090	T	KVQPPPETPAEEEMETETEAEALQEKEQDDT	235.99
890	Q5C9Z4	NOM1	287	T	KAQEAEAQSEDDDEDTEEEQGEEKEKGAQEK	196.66
891	Q8N5F7	NKAP	161	T	WGLSPKNPEPDSDEHTPVEDEEPKKSTTSAS	186.90
892	Q8TF01	PNISR	410	T	DSEDERSDRGSESSDTDDEELRHRIRQKQEA	176.99
893	Q9NYF8	BCLAF1	402	T	KESGKQKFNDSEGDDTEETEDYRQFRKSVLA	174.01
894	Q8IWX8	CHERP	819	T	SYSPGRRRRSRSRSPTPPSSAGLGSNSAPPI	157.33
895	O43583	DENR	86	T	ENSPKQEAGISEGQGTAGEEEEKKKQKRGGR	137.66
896	Q8WVC0	LEO1	188	T	EDKLQNSDDDEKMQNTDDEERPQLSDDERQQ	117.99
897	P51116	FXR2	411	T	GSGRGSGGSDKAGYSTDESSSSSLHATRTYG	108.33
898	Q8N1G4	LRRC47	522	T	KKYTLENKEEGSLSDTEADAVSGQLPDPTTN	105.23
899	Q9H501	ESF1	311	T	SDSGPDLARGKGNIETSSEDEDDTADLFPEE	100.32
900	Q9UQ35	SRRM2	717	T	SRSRSLVRRGRSHSRTPQRRGRSGSSSERKN	98.33
901	P62269	RPS18	60	T	LRKADIDLTKRAGELTEDEVERVITIMQNPR	88.66
902	Q13427	PPIG	748	T	VHEKNKKFDHESSPGTDEDKSG*********	88.06
903	Q13610	PWP1	55	T	KEKLQEEGGGSDEEETGSPSEDGMQSARTQA	84.66
904	Q14684	RRP1B	454	T	LKARVAEPGAEATSSTGEESGSEHPPAVPMH	83.54
905	Q5VZL5	ZMYM4	118	T	SSIHTDDSLEVERRVTQHESDNENEIQIQNK	81.60
906	Q8WVC0	LEO1	302	T	KRKNAIASDSEADSDTEVPKDNSGTMDLFGG	80.61
907	Q9UQ35	SRRM2	1655	T	SSSKGRGPSPEGSSSTESSPEHPPKSRTARR	78.66
908	Q9UQ35	SRRM2	2022	T	RSRTSPVTRRRSRSRTPPAIRRRSRSRTPLL	72.11
909	Q9UQ35	SRRM2	2034	T	RSRTPPAIRRRSRSRTPLLPRKRSRSRSPLA	63.41
910	Q92733	PRCC	261	T	KAAAKSAALQVTKQITQEEDDSDEEVAPENF	59.00

911	Q9NPQ8	RIC8A	441	T	MAGGRPEGQYSEDEDTDTDEYKEAKASINPV	57.64
912	Q9UQ35	SRRM2	2069	T	SRSRTPRTARGKRSLTRSPPAIRRRSASGSS	54.12
913	Q9H501	ESF1	319	T	RGKGNIETSSEDEDDTADLFPEESGFEHAWR	49.85
914	P51116	FXR2	451	T	RRTGGPAYGPSSDVSTASETESEKREEPNRA	49.33
915	Q4G0J3	LARP7	257	T	DTSNTSISKMKRSRPTSEGSDIESTEPQKQC	47.33
916	Q7L590	MCM10	85	T	EKENLATLFGDMEDLTDEEEVPASQSTENRV	47.54
917	Q8IZL8	PELP1	1014	T	PEPEPGLLLEVEEPGTEEERGADTAPTLAPE	46.32
918	Q92541	RTF1	334	T	EEDDKSSEKSDRSSRTSSSDEEEEKEEIPPK	46.22
919	Q96QU1	PCDH15	1863	T	CTTNLMPAEKIKSSMTQLSTTTVCKTDPQRE	42.38
920	O15021	MAST4	1821	T	GTLDIALLSGPQASKTELPSPESAQSPSPSG	42.79
921	Q9H0G5	NSRP1	263	T	SDFDAKSSADDEIEETRVNCRREKVIETPEN	41.33
922	Q9UJV9	DDX41	13	T	***MEESEPERKRARTDEVPAGGSRSEAEDE	39.33
923	Q96ST2	IWS1	317	T	SESEGPQKGPASDSETEDASRHKQKPESDDD	37.33
924	Q8WVC0	LEO1	629	T	SEEDKAQRLLKAKKLTSDEEGEPSGKRKAED	34.52
925	Q96QU1	PCDH15	1867	T	LMPAEKIKSSMTQLSTTTVCKTDPQREPKGI	28.51
926	Q96QU1	PCDH15	1873	T	IKSSMTQLSTTTVCKTDPQREPKGILRHVKN	27.34
927	Q66PJ3	ARL6IP4	343	T	QWHRSAGEEEDGPVLTDEQKSRIQAMKPMTK	7.38
928	Q8NE71	ABCF1	108	T	EEKELMERLKKLSVPTSDEEDEVPAPKPRGG	6.90
929	P27361	MAPK3	202	T	LARIADPEHDHTGFLTEYVATRWYRAPEIML	5.02
930	P27361	MAPK3	207	T	DPEHDHTGFLTEYVATRWYRAPEIMLNSKGY	4.88
931	O75528	TADA3	297	T	IPDMSGKESGADGASTSPRNQNKPFSVPHTK	4.46
932	Q8IYB3	SRRM1	872	T	LAQEEPVAAPEPKKETESEAEDNLDDLEKHL	4.42
933	Q13523	PRPF4B	847	T	LCDFGSASHVADNDITPYLVSRFYRAPEIII	3.60
934	Q9UQ35	SRRM2	577	T	SHSRSPATRGRSRSRTPARRGRSRSRTPARR	3.25
935	Q9UQ35	SRRM2	627	T	RGRSRSRTPARRRSRTRSPVRRRSRSRSPAR	3.19
936	P17544	ATF7	53	T	GPARTDSVIIADQTPTPTRFLKNCEEVGLFN	3.15
937	Q27J81	INF2	1179	T	PAAGPGGDEDEDEEDTAPESALDTSLDKSFS	3.15
938	O15042	U2SURP	931	T	DECTPTRKERKRRHSTSPSPSRSSSGRRVKS	3.05
939	P17544	ATF7	51	T	KFGPARTDSVIIADQTPTPTRFLKNCEEVGL	3.05
940	Q9BVV6	KIAA0586	378	T	NVRLLEQILNNNDSLTRKSESSNTTSLTRSK	2.89
941	P85037	FOXK1	247	T	PDLRSMVSPVPSPTGTISVPNSCPASPRGAG	2.87
942	Q9BW71	HIRIP3	358	T	EPTAKGSRKMARLGSTSGEESDLEREVSDSE	2.81
943	P28482	MAPK1	190	T	DPDHDHTGFLTEYVATRWYRAPEIMLNSKGY	2.75
944	Q9BW71	HIRIP3	84	T	REDKLDLTKKGKRPPTPCSDPERKRFRFNSE	2.67
945	Q13523	PRPF4B	576	T	MSVPSEPSSPQSSTRTRSPSPDDILERVAAD	2.64
946	Q9UQ35	SRRM2	499	T	SRSPATAKRGRSRSRTPTKRGHSRSRSPQWR	2.64
947	P42166	TMPO	74	T	SKGPPDFSSDEEREPTPVLGSGAAAAGRSRA	2.54
948	Q6UN15	FIP1L1	79	T	IEDETAENGVPKPKVTETEDDSDSDSDDDED	2.50
949	P51003	PAPOLA	544	T	SDNSMSVPSPTSATKTSPLNSSGSSQGRNSP	2.45
950	O95218-2	ZRANB2	303	T	SRSRSSSSGDRKKRRTRSRSPESQVIGENTK	2.41
951	P35222	CTNNB1	551	T	LVQLLVRAHQDTQRRTSMGGTQQQFVEGVRM	2.37
952	P06493	CDK1	14	T	**MEDYTKIEKIGEGTYGVVYKGRHKTTGQV	2.27
953	Q13017	ARHGAP5	1217	T	SPVETWKGGIDNPAITSDQELDDKKMKKKTH	2.18
954	Q02156	PRKCE	710	T	VNNFDQDFTREEPVLTLVDEAIVKQINQEEF	2.14
955	P29692	EEF1D	147	T	VSPMRQVEPPAKKPATPAEDDEDDDIDLFGS	2.06
956	Q04759	PRKCQ	536	T	ADFGMCKENMLGDAKTNTFCGTPDYIAPEIL	2.04
957	Q7KZI7	MARK2	208	T	DFGFSNEFTFGNKLDTFCGSPPYAAPELFQG	2.00
958	Q13627	DYRK1A	321	Y	LGQRIYQYIQSRFYR	373.65
959	P25205	MCM3	705	Y	DAKDGDSYDPYDFSD	117.99
960	P45973	CBX5	20	Y	SSEDEEEYVVEKVLD	61.34
961	O60841	EIF5B	134	Y	AKPKVEMYSGSDDDD	59.54
962	O15541	RNF113A	80	Y	SGKQKAAYGDLSSEE	59.14
963		PREDICTED:	113	Y	MVSFIAVYTSHLS--	59.00
964	P98175	KFZp686E245	122	Y	SQEGKHDYDDSSEEQ	57.64
965	O15173	PGRMC2	179	Y	EGEEPTVYSDEEEPK	49.31
966	P51116	FXR2	409	Y	GGSDKAGYSTDESSS	39.33
967	P06493	CDK1	15	Y	EKIGEGTYGVVYKGR	8.63

968	B4DWW4	MCM3	753	Y	DGDSYDPYDFSDTEE	7.42
969	Q9HCE7	SMURF1	113	Y	SRLKDTGYQRLDLCK	7.19
970	P27361	MAPK3	204	Y	HTGFLTEYVATRWYR	4.74
971	Q13523	PRPF4B	849	Y	ADNDITPYLVSRFYR	4.61
972	P49840	GSK3A	279	Y	RGEPNVSYICSRYYR	2.84
973	G3V5T9	CDK2	15	Y	EKIGEGTYGVVYKAR	2.27
974	Q8IZT6	ASPM	2564	Y	SIVIQSTYRMYRQYC	2.11

Table S1: Identified phosphorylation sites are list with acession information, phosphorylation site with positions in the proteins, spanning amino acids sequnces and fold of changes. Protein identifications overlapped with protein array analysis (Keller *et al*., *Mol Cell* 2014) were highlited in blue.

Supplemental Table S2

No.	Sample ID	Gender	Age	Histology (WHO Grade)
1	2014-06517	M	51	Renal Clear Cell Carcinoma (Ⅱ)
2	2014-03303	M	38	Renal Clear Cell Carcinoma (Ⅱ)
3	2014-01577	F	64	Renal Clear Cell Carcinoma (Ⅱ-III)
4	2014-05726	F	78	Renal Clear Cell Carcinoma (Ⅰ-Ⅱ)
5	2014-02433	M	69	Renal Clear Cell Carcinoma (Ⅱ)
6	2013-05583	F	40	Renal Clear Cell Carcinoma (Ⅱ)
7	2013-05739	F	62	Renal Clear Cell Carcinoma (Ⅱ-III)
8	2013-06867	M	32	Renal Clear Cell Carcinoma (Ⅰ-Ⅱ)
9	2014-00532	M	77	Renal Clear Cell Carcinoma (Ⅱ-III)
10	2014-00862	M	58	Renal Clear Cell Carcinoma (Ⅱ)
1	2015-05563	M	38	Breast Infiltrating Ductal Carcinoma (Ⅱ)
2	2015-06795	M	51	Breast Infiltrating Ductal Carcinoma (Ⅱ-III)
3	2015-19494	M	45	Breast Infiltrating Ductal Carcinoma (III)
4	2015-19651	M	39	Breast Infiltrating Ductal Carcinoma (Ⅱ)
5	2015-19745	M	53	Breast Infiltrating Ductal Carcinoma (III)
6	2015-19926	M	49	Breast Infiltrating Apocrine Carcinoma (Ⅱ-III)
7	2015-20046	M	69	Breast Infiltrating Ductal Carcinoma (Ⅱ)
8	2015-20132	M	47	Breast Infiltrating Ductal Carcinoma (Ⅱ)
9	2015-20133	M	62	Breast Infiltrating Ductal Carcinoma (Ⅱ)
10	2015-20135	M	36	Breast Infiltrating Ductal Carcinoma (Ⅱ)

Supplemental Table S2, Information of RCC and breast cancer samples were obtained and listed (related to Figure 7A&B and Figure S6).

Supplementary Experimental Procedures

Antibodies

Ser202, S203 and S202/203 specific phosphorylation antibodies were homemade. AKT1S1 antibody (#2610), PKM2 antibody (#3198), p70 S6 kinase antibody (#9202), Phospho-p70 S6 kinase (Thr389) antibody (#9205), LC3B antibody (#2775), 4E-BP1 (53H11) rabbit mAb (#9644) and Phospho-4E-BP1 (Thr37/46) (236B4) Rabbit mAb (#2855) were purchased from Cell Signaling Technology. Anti-SQSTM1 / p62 antibody (ab56416) was from abcam.

In vitro PKM2 phosphorylation of HEK293T proteome

HEK293T proteome were prepared as following: HEK293T cells were cultured in DMEM medium with 10% newborn bovine serum, 100 units/ml penicillin and 100 μg/ml streptomycin in a humidified atmosphere of 5% CO_2 at 37℃. Harvested cells were collected, washed with PBS (pH7.5) and lysed in 50 mM HEPES buffer (pH 7.5) buffer containing 1% Triton (v/v) , 65 mM DTT , 1 mM PMSF, 2% proteases inhibitors cocktail (Roche) for 30 minutes. After centrifugation at 12000 rpm at 4℃ for 30 minutes, the supernatant was collected and subject to either acetone precipitation followed by PKM2 phosphorylation or immobilization to CNBr-activated Sepharose for dephosphorylation and phosphorylation. For proteome dephosphorylation, 50U alkaline phosphatase (Sigma) was incubated with immobilized proteins in 1mM MgCl2, 1mM ZnCl2, 50 mM HEPES (pH 7.5). The resulted dephosphorylated immobilized proteome were washed with PBS (pH7.5) overnight to remove alkaline phosphatase and any small molecules that may serve as phosphor-donor.

In vitro kinase reaction

The proteins or dephosphorylated immobilized proteome were preincubated in kinase buffer (100mM KCl, 50mM $MgCl_2$, 1mM DTT, 1mM $NaVO_4$, 5% glycerol, 30mM HEPES (pH 7.6)) at 30℃ for 30min. *In vitro* kinase reaction was triggered by adding 50 μg recombinant PKM2 and 0.5mM PEP, while in the control experiment only

contains 0.5 mM PEP. Both reactions were allowed to process at 30℃ for 2 hours before 100 mM ammonium bicarbonate (NH_4HCO_3) was added to stop the reaction.

Trypsin digestion and phosphopeptides enrichment

The washed immobilized proteome were incubated with 20 mM DTT for 2 hours at 37℃, followed by addition of 60 mM iodoacetic acid to the reaction mixture. After incubation in the dark for 1 hour at 25℃, trypsin was added at 50 μg/ mg protein in 100mM NH_4HCO_3 buffer (pH 8.0). After digestion for 18h at 37℃, the released peptides were collected and acidized by 5% trifluoroacetic acid before they were subjected to phosphopeptides enrichment by Ti41-IMAC microspheres.

Mass Spectrometry

The mass spectrometer was set as below: ion transfer capillary 200 ℃, spray voltage 1.8 kV, full MS range 400-2000, and full mass spectra was acquired in the Orbitrap at a resolution of 60,000 with the target ion setting of 10^6. One full MS scan was followed by ten MS/MS scans, and the multistage activation was enabled. The dynamic exclusion function was set as follows: repeat count 2, repeat duration 30 s, and exclusion duration 60 s. Each sample was analyzed five times by the RPLC-MS/MS.

Gene silence

Synthetic oligos were used for *siRNA* silencing of *AKT1S1* and *PKM2*, the sequence used for silencing genes are:

AKT1S1: –GCTGAGTTCTAAGCTCTAAAT-

PKM2: -CTGCAGCGTTGTTAGCAAATAAT-

For *shRNA* silencing of genes, above sequences were cloned into PMKO vector, the plasmids constructed were cotransfected into HEK293T cells together with the *gag* and *vsvg* genes expressing plasmids to produce retrovirus. Retroviral supernatant was harvested 36 hr after initial plasmid transfection and mixed with polybrene (8 mg/ml) to increase the infection efficiency. Cells were infected with retrovirus and selected in puromycin (1 μg/ml) for 2 weeks. Knockdown efficiencies were analyzed by either western blots or Q-PCR.

Bioinformatics analysis

All analysis were performed using published software. The secondary structures and solvent accessibilities of HPSRPs were predicted with NetSurfP 1.1 (Petersen et al., 2009), while the disorder regions and sub-cellular localizations were predicted by ESpritz (Walsh et al., 2012) and WoLF PSORT ({Qi et al., 2007), repectively. The gene ontology annotations were downloaded from QuickGO database (Barrell et al., 2009), while the enrichment analyses were performed with a hypergeometric distribution as previously described (Qi et al., 2014). The enrichment analyses of KEGG pathways were carried out using DAVID (Huang da et al., 2009), which also presented the pathways with annotated HPSPs. All the heatmaps were visualized with the ggplot2 program (http://had.co.nz/ggplot2/) in the R package (http://www.r-project.org/). The amino acid preferences were visualized with WebLogo (Crooks et al., 2004), while the comparisons of amino acid preferences were visualized with Two Sample Logo (Vacic et al., 2006).

Immunohistochemistry

Tissue sections were prepared form the formalin-fixed paraffin embedded specimens. Antigen retrieval of renal cell carcinoma or breast cancer specimens was performed by incubating the slides in Tris-EDTA buffer (pH 8.4) at 99℃ for 60 mins. The endogenous peroxidase activity was inactivated in solution of methanol with 3% H_2O_2. The slides were incubated with primary antibody for 60 mins and secondary antibody for 8 mins, then stained with DAB Chromagen for 8 mins. All procedures were performed using stainer (BenchMark XT, Ventana) and the slides were scaned by scanner (Ventana iScan Coreo). The quantification of IHC results were performed by an experienced pathologist. The intensity was calculated according to positive areas and positive degree. Sections were staining with PKM2 (1:100), P-S202/203-AKT1S1 (1:30), P-S2448-mTOR (1:100), P-T37/46-4EBP1 (1:500), p62 (1:200) and LC3B (1:100) antibody using a Ultraview Detection Kit.

Supplementary References

Qi, H., P., Park, K.J., Obayashi, T., Fujita, N., Harada, H., Adams-Collier, C.J., and Nakai, K. (2007). WoLF PSORT: protein localization predictor. Nucleic acids research *35*, W585-587.

Barrell, D., Dimmer, E., Huntley, R.P., Binns, D., O'Donovan, C., and Apweiler, R. (2009). The GOA database in 2009--an integrated Gene Ontology Annotation resource. Nucleic acids research *37*, D396-403.

Crooks, G.E., Hon, G., Chandonia, J.M., and Brenner, S.E. (2004). WebLogo: a sequence logo generator. Genome research *14*, 1188-1190.

Huang da, W., Sherman, B.T., and Lempicki, R.A. (2009). Systematic and integrative analysis of large gene lists using DAVID bioinformatics resources. Nature protocols *4*, 44-57.

Petersen, B., Petersen, T.N., Andersen, P., Nielsen, M., and Lundegaard, C. (2009). A generic method for assignment of reliability scores applied to solvent accessibility predictions. BMC structural biology *9*, 51.

Qi, L., Liu, Z., Wang, J., Cui, Y., Guo, Y., Zhou, T., Zhou, Z., Guo, X., Xue, Y., and Sha, J. (2014). Systematic Analysis of the Phosphoproteome and Kinase-substrate Networks in the Mouse Testis. Molecular & cellular proteomics : MCP *13*, 3626-3638.

Vacic, V., Iakoucheva, L.M., and Radivojac, P. (2006). Two Sample Logo: a graphical representation of the differences between two sets of sequence alignments. Bioinformatics *22*, 1536-1537.

Walsh, I., Martin, A.J., Di Domenico, T., and Tosatto, S.C. (2012). ESpritz: accurate and fast prediction of protein disorder. Bioinformatics *28*, 503-509.
